# Health and economic impacts of ambient air pollution on hospital admissions for overall and specific cardiovascular diseases in Panzhihua, Southwestern China

**DOI:** 10.7189/jogh.12.11012

**Published:** 2022-12-21

**Authors:** Xianzhi Li, Yajie Li, Bin Yu, Hongwei Zhu, Zonglei Zhou, Yan Yang, Shunjin Liu, Yunyun Tian, Junjie Xiao, Xiangyi Xing, Li Yin

**Affiliations:** 1 Meteorological Medical Research Center, Panzhihua Central Hospital, Panzhihua, China; 2 Tibet Center for Disease Control and Prevention, Lhasa, China; 3 Institute for Disaster Management and Reconstruction, Sichuan University – Hong Kong Polytechnic University, Chengdu, China; 4 Department of dermatology, Panzhihua Central Hospital, Panzhihua, China; 5 Department of Epidemiology, School of Public Health, Fudan University, Shanghai, China; 6 Department of Respiratory and Critical Care Medicine, Panzhihua Central Hospital, Panzhihua, China; 7 Department of Medical Records and Statistics, Panzhihua Central Hospital, Panzhihua, China; 8 Department of Pharmacy, Panzhihua Central Hospital, Panzhihua, China

## Abstract

**Background:**

The associations of ambient air pollution with hospital admissions (HAs) for overall and specific causes of cardiovascular diseases (CVDs), as well as related morbidity and economic burdens remain understudied, especially in low-pollution areas of low- and middle-income countries (LMICs). We evaluated the short-term effects of exposure to PM_2.5_ (particles with an aerodynamic diameter ≤2.5 μm), PM_10_ (particles with an aerodynamic diameter ≤10 μm), and SO_2_ (sulfur dioxide) on HAs for CVDs in Panzhihua, China, during 2016-2020, and calculated corresponding attributable risks and economic burden.

**Methods:**

We used a generalized additive model (GAM) while controlling for time trends, meteorological conditions, holidays, and days of the week to estimate the associations. The cost of illness (COI) method was adopted to further assess corresponding hospitalization costs and productivity losses.

**Results:**

A total of 27 660 HAs for CVDs were included in this study. PM_10_ and SO_2_ were significantly associated with elevated risks of CVDs hospitalizations. Each 10 μg/m^3^ increase in PM_10_ and SO_2_ at lag06 corresponded to an increase of 2.48% (95% confidence interval (CI) = 0.92%-4.06%), and 5.50% (95% CI = 3.09%-7.97%) in risk of HAs for CVDs, respectively. The risk estimates of PM_10_ and SO_2_ on CVD hospitalizations were generally robust after adjustment for other pollutants in two-pollutant models. We found stronger associations between air pollution (PM_10_ and SO_2_) and CVDs in cool seasons than in warm seasons. For specific causes of CVDs, significant associations of PM_10_ and SO_2_ exposure with cerebrovascular disease and ischaemic heart disease were observed. Using 0 μg/m^3^ as the reference concentrations, 11.91% (95%CI = 4.64%-18.56%) and 15.71% (95%CI = 9.30%-21.60%) of HAs for CVDs could be attributable to PM_10_ and SO_2_, respectively. During the study period, PM_10_ and SO_2_ brought 144.34 million Yuan economic losses for overall CVDs, accounting for 0.028% of local GDP.

**Conclusions:**

Our results suggest that PM_10_ and SO_2_ exposure might be an important trigger of HAs for CVDs and accounted for substantial morbidity and economic burden.

Cardiovascular diseases (CVDs) are disorders, abnormalities, or failures in the function of the heart, blood vessels, or circulation [1]. With the growing globalization, urbanization, and population ageing, CVDs have been the leading cause of death globally, bringing a heavy social and economic burden to countries worldwide [2]. The World Health Organization (WHO) estimated that 17.9 million people died of CVDs, accounting for 32% of the world’s nonaccidental deaths, while over three-quarters of CVDs deaths took place in low- and middle-income countries (LMIC) in 2019 [3].

As multi-factorial disorders, CVDs are influenced by heredity, living conditions, and behavioural lifestyle factors [4]. Likewise, air pollution is a major risk factor for CVDs [5]. Numerous epidemiological studies have demonstrated the short effects of particulate matter (PM) concentrations and airborne contaminants on mortality, emergency department visits, and hospital admissions (HAs) for CVDs [6-9], especially in low socioeconomic and elderly populations [10]. However, previous research mainly focused on developed countries in North America and Europe [11-14]. A study from New England found that short-term exposure to an increased ambient PM_2.5_ (particles with an aerodynamic diameter ≤2.5 μm) level could potentially increase the risk of CVDs hospitalization [6]. In London, Poloniecki et al. [15] confirmed the significant effects of nitrogen dioxide (NO_2_), carbon monoxide (CO) and sulfur dioxide (SO_2_) on emergency HAs for circulatory diseases. The association between PM_10_ (particles with an aerodynamic diameter ≤10μm) and CVD mortality was found in a study including 90 American cities [16]. Although the link between air pollution and health is well documented, the effect of air pollutants on cardiovascular health remains controversial. For example, some studies have suggested independent health effects of SO_2_ on cardiovascular health [1,15,17] and others indicated that SO_2_ might be not a predictor for increased CVDs HAs [18]. More research is needed to confirm whether this association between air pollutants and CVDs exists, especially in LMICs.

Few studies focused on the effect of air pollution on CVDs have been conducted in Asian LMICs, including mainland China [9,19-21], Thailand [17], and India [22]. Almost all have focused on large cities with relatively high economic levels, serious air pollution, and low degree of ageing, such as Beijing [19], Shanghai [20], Guangzhou [9], Wuhan [21], Delhi [22], and Bangkok [17]. Bateson et al. [10] found that the effects of air pollution on health may be more pronounced among people with low incomes and the elderly. An earlier study reported that associations between air pollution and CVDs hospitalization were substantially stronger in areas with a lower concentration of air pollutants [23]. Thus, studies focusing on areas with low levels of air pollution and high levels of ageing are needed.

Located in southwest China, Panzhihua has good air quality and has been awarded the title of “Most Liveable City of China” in 2022 [24]. It is also one of the cities with the highest ageing level in Sichuan Province, at more than 14% (the cut-off of a moderately ageing society), making it a good site for studying the health effects of air pollution in areas with low concentrations of air pollutants and high levels of ageing. Located in the upper reaches of the Yangtze River, Panzhihua shows typical dry-hot valley (DHV) climate, characterized by high temperatures (annual mean >20°C) and low humidity (annual mean precipitation <650 mm) [25]. It has been speculated that the difference in climate conditions, such as temperature, may facilitate either the accumulation or dispersal of air pollutants, which may be related to CVDs incidence [17,26]. Unfortunately, no studies exploring the effect of air pollution on CVDs have been conducted in DHV regions.

Moreover, relatively few studies have estimated the attributable risk of HAs for CVDs due to PM_2.5_, PM_10_, and SO_2_ exposure [27-30] and the economic costs of CVDs due to PM_2.5_ and PM_10_ exposure [27,29,30] in mainland China, as well as the economic costs of CVDs due to SO_2_ exposure. Compared with measurements such as relative risk, attributable risk measures (such as attributable fraction (AF) and attributable number (AN)) and economic cost were more suitable and intuitive for researchers and policymakers to evaluate the threat of air pollutants to health.

In this study, we aimed to conduct a time-series analysis to detect the short-term effects of exposure to ambient air pollutants (PM_2.5_, PM_10_, SO_2_) on the risk of HAs for total CVDs and two cause-specific CVDs (cerebrovascular diseases and ischaemic heart disease) within a five-year period in Panzhihua, southwestern China. We further planned to estimate the attributable risk and economic costs of HAs for CVDs due to PM_2.5_, PM_10_, and SO_2_ exposure.

## METHODS

### Study area

Panzhihua (108°08'-102°15' east, 26°05'-27°21' north) is located at the border between southwestern Sichuan Province and northwestern Yunnan Province, and it has a typical DHV climate (characterized by high temperature and low humidity). By the end of 2020, the population of Panzhihua was 1.12 million, of which 1.08 million are registered residents. Panzhihua has three districts and two counties, with a total area of 7414 km^2^. We selected all the districts and counties of Panzhihua as the study area.

### Air pollution and meteorology data

The 24-hour mean concentrations of PM_2.5_, PM_10_, SO_2_, NO_2_, CO, and daily maximum eight-hour average ozone (O_3_) concentration from January 1, 2016, to December 31, 2020, were derived from the Panzhihua Environmental Monitoring Center [31]. During the study period of a total of 1827 days, there were no missing values for the average daily concentrations of the six air pollutants.

The daily meteorological factors, including relative humidity, average temperature, atmospheric pressure, and wind speed from January 1, 2016, to December 31, 2020, were obtained from the Panzhihua Meteorological Bureau [32]. During the study period, about 1.2% of days had missing values. We used the average of the two days before and after the day containing the missing value to fill it in.

### Daily cardiovascular admissions

Daily numbers of HAs due to cardiovascular causes were collected from January 1, 2016, to December 31, 2020, from the Health Information Center of Panzhihua [33], which contained the hospitalization records of all the tertiary and secondary hospitals in the study area. From each hospitalization, we extracted the following details: age, sex, residential address, admission date, length of hospital stays, hospital cost, and principal discharge ICD-10 (International Classification of Diseases, 10th Revision) codes [34]. HAs for the selected CVDs included CVDs: I00-I99; ischaemic heart disease: I10-I25; and cerebrovascular disease: I60-I69.

### Local GDP

We collected the annual GDP data of Panzhihua during 2016-2020 from the Panzhihua Bureau of Statistics [35].

### Statistical methods

#### Descriptive statistics

We described daily CVDs HAs, air pollutants, and meteorological factors using means, standard deviations (SDs), and quartiles. We utilized Spearman rank correlation analysis to estimate the relationships between air pollutants and meteorological factors.

#### Estimating associations

We applied an over-dispersed generalized additive model (GAM) which allowed the quasi-Poisson distribution to explore the short-term effect of air pollutants on HAs for CVDs. We used cubic spline functions to control the potential confounding effect of the day of week (DOW), long-term trends and seasonality, weather conditions (relative humidity, average temperature), and public holidays in the model. Partial autocorrelation function (PACF) was applied to select the degree of freedom for long-term trends and seasonality by minimizing the absolute values of the sum of PACF for lags up to 30. According to previous research and the results of PACF (Figure S1 in the **Online Supplementary Document**), the degree of freedom (*df*) for the long-term trend and seasonality was determined to be seven per year [4,36,37]. The *df* of mean temperature and relative humidity were both set to three [4,26,36]. Holiday and DOW were included as categorical variables. The basic model was as follows:

log[E(Y_t_] = ꞵZ_t_ + ns(time, 7 × 5) + ns(Temp_t_, 3) + ns(RHt,3) + DOW + Holiday + α

Where *t* denoted the day of the observation, *E(Y_t_)* represented expected numbers of HAs due to CVDs on day *t*, β was the log-relative rate of CVDs admissions associated with per unit increment of air pollutants, *Z_t_* indicated the concentration of air pollutants on day *t*, *ns* represented natural smooth splined function, *time* was the days of calendar time on day *t* used to control the long-term trend and seasonality of time, *Temp_t_* refered to the temperature on day *t*, *RH_t_* indicated the relative humidity on day *t*, *DOW* was day of the week, and α denoted the intercept.

We incorporated both single-day lag (from current day to six days before: lag0-lag6) and multi-day moving average (from lag01 to lag06) to estimate lag patterns of ambient air pollutants, fitting separate models with the same parameter settings as in the basic model for each lag day.

We also performed subgroup analyses to explore the effects of modification by gender (male *vs* female), age (<70 vs ≥70), and season (warm *vs* cold). The warm season was defined as a period from May to October and the cool season as November through April [38]. And we also tested the statistical significance of the differences between subgroups using Z-test as the equation listed below:

*Z = (Q1 – Q2) / sqrt(SE_1_^2^ + SE_2_^2^)*

where *Q_1_* and *Q_2_* refer to the effect estimates and SE*_1_* and SE*_2_* refer to the standard error for the subgroups [39].

Sensitivity analyses were conducted to check the robustness of our findings as follows: 1) we built two-pollutant models to test the stability of the effects after adjustment for other pollutants; considering the collinearity between air pollutants, PM_10_ and PM_2.5_ do not appear in the model simultaneously; 2) we used different *df* of long-term trend (5-10 *df* per year) to evaluate the associations between air pollutants and HAs for CVDs; 3) it has been suggested that wind speed and atmospheric pressure were associated with air pollution-related health effects [40], so we added the two variables into our model.

**Estimating the attributable risk due to air pollutants**

AF and AN based on the air pollutants-HAs for CVDs association were used to estimate the attributable risk of HAs for total CVDs and cause-specific CVDs due to exceeding air pollutants exposure:

*AF = Σ {baseline patients ×* [*1 – exp( –β × ΔY*]} */ total patients*

*AF* = Σ{*baseline patients* × [1 *– exp*(*–ꞵ × ΔY*)]}

where baseline patients are the CVD patient counts on a specific day, β is the exposure–response relation coefficient of the HAs effects of air pollutants derived from the GAM, and *∆Y* indicates the difference in daily air pollutants concentration with the threshold level at which no adverse health effects occur. According to the WHO, there is little evidence for a threshold for PM and SO_2_ below which no harmful health effects could be anticipated [41]. Therefore, 0 μg/m^3^ was selected as the threshold concentration of PM and SO_2_.

#### Evaluating the corresponding hospitalization economic loses

The cost of illness method (COI) was adopted to evaluate corresponding hospitalization economic burden of CVDs due to air pollutants from patient perspective, which considered both medical expenses (including medicines, diagnostics, medicinal materials, and medical services) incurred when a person is sick (the direct costs of illness) and loss in productivity when a person is in hospital (indirect costs of illness) [42,43]. Due to the absence of income information for each hospital admission, we used the regional daily GDP per capita to calculate productivity loss [43]. We calculated the economic losses as follows:

*meanC* = *C_h_* + *dGDPp* × *meanT_h_*

Δ*C* = *AN* × *meanC*

where *meanC* is the average total economic costs per hospital admission, *C_h_* refers to the mean treatment cost per hospital admission calculated according to our data in Panzhihua in 2016–2020, *dGDPp* indicates daily GDP per capita, *meanT_h_* represents mean hospitalization days per hospital admission, and *ΔC* indicates the total economic loss due to air pollution during the study period.

All analyses were conducted using R software (version 4.2.1). The results were expressed as excess risk (ER) and 95% confidence interval (CI) in risk of daily HAs per 10μg/m^3^ increase of PM or SO_2_ concentrations. *P* < 0.05 was considered statistically significant.

## RESULTS

### Basic characteristics

A total of 27 660 HAs for CVDs were collected (9782 for cerebrovascular diseases and 7034 for ischaemic heart disease) **(****Table 1****)**. The patients aged ≥70 years accounted for 49.0% of the total sample, which consisted of 15 529 male patients and 12 131 female patients. Hospitalizations for CVDs in the warm season (50.3%) were similar to those in the cool season (49.7%). Daily mean concentrations were 28.2 μg/m^3^ for PM_2.5_, 51.9 μg/m^3^ for PM_10_, 32.0 μg/m^3^ for SO_2_, 33.5 μg/m^3^ for NO_2_, 1.39 mg/m^3^ for CO, 80.8 μg/m^3^ for O_3_. Daily mean temperature and daily mean relative humidity were 21.0°C and 58.7%, respectively. Spearman's correlations between daily levels of air pollutants and meteorological variables suggested the strongest correlation between PM_2.5_ and PM_10_ (Figure S2 in the **Online Supplementary Document**).

**Table 1:** Summary statistics for cardiovascular disorders hospitalizations, air pollutants concentrations, and meteorological variables in Panzhihua, 2016-2020

	N (%)	Daily mean ± SD
**Cardiovascular disorders**	27 660 (100.0)	15 ± 8
Cerebrovascular diseases	9782 (35.4)	5 ± 3
Ischaemic heart disease	7034 (25.4)	4 ± 3
**Age (years)**		
0-9	14 101 (51.0)	6 ± 4
<70	13 559 (49.0)	10 ± 5
**Gender**		
Male	15 529 (56.1)	9 ± 5
Female	12 131 (43.9)	7 ± 4
**Season**		
Warm	13 908 (50.3)	15 ± 8
Cool	13 752 (49.7)	15 ± 8
**Air pollutants**		
PM_2.5_ (μg/m^3^)	-	28.2 ± 11.7
PM_10_ (μg/m^3^)	-	51.9 ± 18.3
SO_2_ (μg/m^3^)	-	32.0 ± 13.3
NO_2_ (μg/m^3^)	-	33.5 ± 10.2
CO (mg/m^3^)	-	1.4 ± 0.5
O_3_ (μg/m^3^)	-	80.8 ± 29.9
**Meteorological variable**s		
Temperature (°C)	-	21.0 ± 5.5
Relative humidity (%)	-	58.7 ± 18.3

SD – standard deviation

### Associations of air pollutants with HAs for CVDs

**Figure 1:**
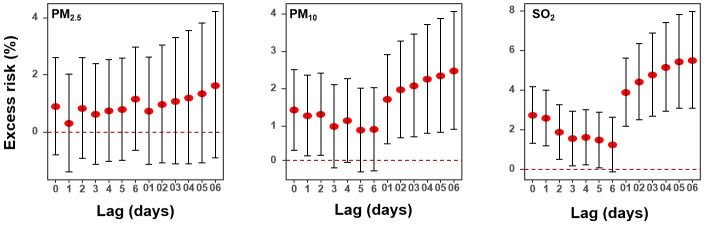
Excess risk (95% confidence interval, CI) in hospital admissions for cardiovascular disorders associated with a 10 μg/m^3^ increase in PM_2.5_, PM_10_, and SO_2_ along different lag structures using single pollutant models.

**Figure 1** shows the associations of air pollutants with HAs for CVDs using single-pollutant models along different lag structures. The effects of PM_2.5_ on total CVDs were not generally significant on all the lag days. The effects of PM_10_ on total CVDs were generally significant on lag0, lag1, lag2, and all the cumulative lag days, with the largest effects on lag06-day. The ER per 10 μg/m^3^ increment in PM_10_ at lag06 was 2.48% (95% CI = 0.92, 4.06). The effects of SO_2_ on total CVDs were generally significant on lag0, lag1, lag2, lag3, lag4, lag5, and all the cumulative lag days, with the largest effects on lag06-day. The ER per 10 μg/m^3^ increment in SO_2_ at lag06 was 5.50% (95% CI = 3.09, 7.97).

### Stratified analyses

**Figure 2:**
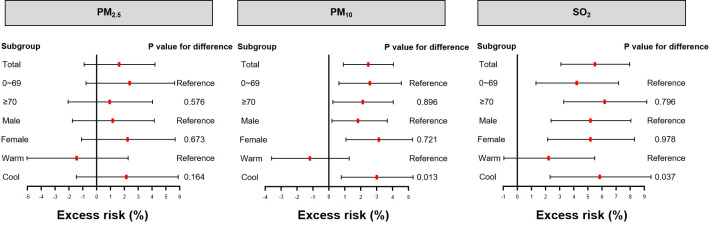
Forest plot of excess risk (95% confidence interval, CI) in hospital admissions for cardiovascular disorders by age, sex, and season associated with a 10 μg/m^3^ increase in PM_2.5_, PM_10_, and SO_2_ at lag06 using single pollutant models.

**Figure 2** illustrates the effect estimates of stratified analyses by age, gender, and season. The effects of PM_2.5_ on HAs for CVDs were non-significant in all subgroups. In age-specific analyses of PM_10_ and SO_2_, the effects were statistically significant. In gender-specific analyses of PM_10_ and SO_2_, the effects were more pronounced in females than in males, but the difference was not statistically significant. Regarding the differences in season groups for PM_10_ and SO_2_, we observed positive and significant associations in cool seasons, while unsignificant associations were found in warm seasons, and the differences were statistically significant.

### Sensitivity analyses

**Table 2** shows the two-pollutant models. Effect estimates of air pollutants on HAs for CVDs were still significant on lag06 after adjustment for other air pollutants. The effect estimates for two-pollutant models along different lag structures were shown in Table S1 in the **Online Supplementary Document**. Additionally, after the adjustment of smoothness of time (5–10 *df* per year), our results remained relatively stable (Table S2 in the **Online Supplementary Document**). After adding wind speed and atmospheric pressure into the single-pollutant models, the results remained robust to these additional controls (Table S3 in the **Online Supplementary Document**).

**Table 2:** Excess risk in hospital admissions for cardiovascular disorders associated with a 10 μg/m^3^ increase in PM_2.5_, PM_10_, and SO_2_ at lag06 using single- and two-pollutant models

	PM_2.5_	PM_10_	SO_2_
**None**	1.62 (95% CI = -0.9, 4.21; *P* = 0.210)	2.48 (95% CI = 0.92, 4.06; *P* = 0.002)	5.50 (95% CI = 3.09, 7.97; *P* < 0.001)
+PM_2.5_	-	-	5.49 (95% CI = 3.02, 8.01; *P* < 0.001)
**+PM**_10_	-	-	5.00 (95% CI = 2.50, 7.55; *P* < 0.001)
**+SO**_2_	0.75 (95% CI = -1.79, 3.37; *P* = 0.565)	1.75 (95% CI = 0.15, 3.38; *P* = 0.032)	-
**+NO**_2_	0.85 (95% CI = -1.89, 3.67; *P* = 0.545)	2.36 (95% CI = 0.60, 4.15; *P* = 0.008)	5.27 (95% CI = 2.81, 7.79; *P* < 0.001)
+CO	1.84 (95% CI = -0.84, 4.59; *P* = 0.180)	2.73 (95% CI = 1.06, 4.44; *P* = 0.001)	6.02 (95% CI = 3.50, 8.61; *P* < 0.001)
**+O**_3_	2.08 (95% CI = -0.52, 4.75; *P* = 0.118)	2.63 (95% CI = 1.06, 4.23) (*P* < 0.001)	5.38 (95% CI = 2.96, 7.86; *P* < 0.001)

CI – confidence interval

### Associations of air pollutants with HAs for specific causes of CVDs

**Figure 3:**
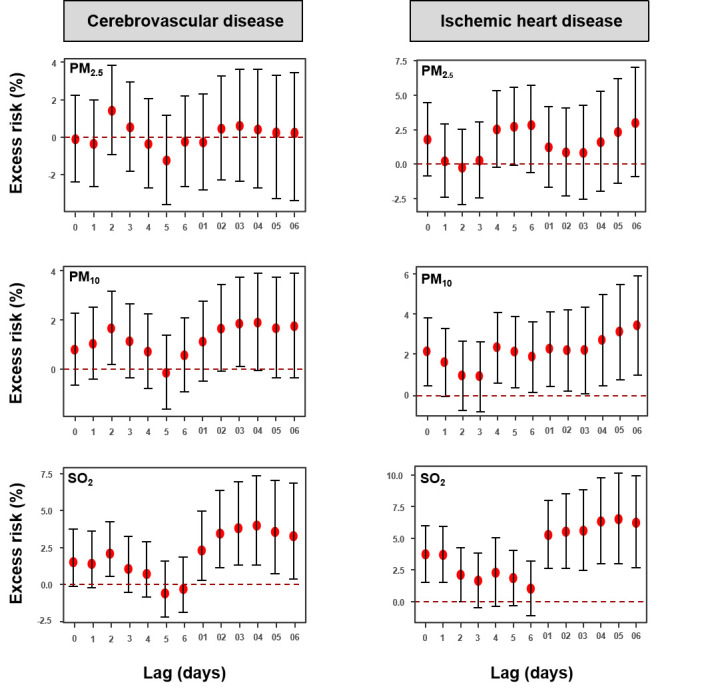
Excess risk (95% confidence intercal, CI) in hospital admissions for specific causes of cardiovascular disorders (cardiovascular disease, and ischemic heart disease) associated with 10 μg/m^3^ increase in PM_2.5_, PM_10_, and SO_2_ along different lag structures using single pollutant models.

**Figure 3** shows the associations of air pollutants with HAs for subcategories of CVDs. Overall, we found insignificant associations of PM_2.5_ with two subcategories of CVDs on all the lag days. Additionally, we found significant associations of PM_10_ with cerebrovascular diseases on lag2 and lag03, respectively, and the lag2-day generally produced the largest estimates (ER = 1.67%, 95% CI = 0.19, 3.17). We also found significant associations of PM_10_ with ischaemic heart disease on lag0, lag4, lag5, and lag6 and all the cumulative lag days, and the lag06-day generally produced the largest estimates (ER = 3.44%, 95% CI = 0.99, 5.94). The effects of SO_2_ on cerebrovascular diseases on lag3 and all the cumulative lag days were significant, and the lag04-day generally produced the largest estimates (ER = 4.36%, 95% CI = 1.39, 7.42). We also found significant associations of SO_2_ with ischaemic heart disease on lag0, lag1, lag2, and all the cumulative lag days, and the lag05-day generally produced the largest estimates (ER = 6.51%, 95% CI = 3.01, 10.13).

### Attributable risk and economic costs due to exceeding air pollutants exposure

**Table 3** summarizes the estimation of AF, AN, and economic costs attributable to exceeding air pollutants exposure using 0 μg/m^3^ as the reference concentrations. Overall, 11.91% (95% CI = 4.64, 18.56), and 15.71% (95% CI = 9.30, 21.60) of HAs for CVDs could be attributable to exceeding PM_10_ and SO_2_, respectively. Additionally, we found that approximately 8.12% (95% CI = 0.95, 14.72) and 12.65% (95% CI = 4.31, 20.16) of cerebrovascular disease HAs to be attributed to PM_10_ and SO_2_, respectively, and approximately 16.10% (95% CI = 9.51, 22.16) and 12.82% (95% CI = 4.37, 20.41) of ischaemic heart disease HAs to be attributed to PM_10_ and SO_2_, respectively. The total economic costs of HAs for overall CVDs, cerebrovascular diseases, and ischaemic heart disease due to air pollution were 144.34 (95% CI = 72.89, 209.80), 42.48 (95% CI = 10.74, 71.32), and 39.55 (95% CI = 18.98, 58.23) million Yuan, respectively. Regarding different pollutants, the greatest economic burden came from SO_2_, which reached 82.11 (95% CI = 48.63, 112.84) million Yuan for overall CVDs, followed by PM_10_.

**Table 3:** The attributable fraction, attributable number, and economic costs of hospital admissions due to ambient air pollutants in Panzhihua, 2016-2021

Cause		PM_2.5_		PM_10_		SO_2_		Average total cost per HAs§		Total costs‖	
	AF (%, 95% CI)*	**AN (No, 95% CI)†**	**Costs (95% CI)‡**	**AF (%, 95% CI)**	**AN (No, 95% CI)**	**Costs (95% CI)**	**AF (%, 95% CI)**	**AN (No, 95%CI)**	**Costs (95% CI)**		
**Cardiovascular disease**	0 (0, 0)	0 (0, 0)	0 (0, 0)	11.91 (4.64, 18.56)	3294 (1284, 5132)	62.23 (24.26, 96.96)	15.71 (9.30, 21.60)	4346 (2574, 5973)	82.11 (48.63, 112.84)	18897.72	144.34 (72.89, 209.80)
**Cerebrovascular disease**	0 (0, 0)	0 (0, 0)	0 (0, 0)	8.12 (0.95, 14.72)	795 (93, 1440)	16.62 (1.94, 30.10)	12.65 (4.31, 20.16)	1237 (421, 1972)	25.86 (8.80, 41.22)	20908.83	42.48 (10.74, 71.32)
**Ischaemic heart disease**	0 (0, 0)	0 (0, 0)	0 (0, 0)	16.10 (9.51, 22.16)	1133 (669, 1559)	22.03 (13.01, 30.31)	12.82 (4.37, 20.41)	901 (307, 1436)	17.52 (5.97, 27.92)	19444.98	39.55 (18.98, 58.23)

AF – attributable fraction, AN – attributable number, HA – hospital admission, CI – confidence interval

*AF was calculated based on the largest effect estimates in single pollutant models; we set AF to zero when there is no significant association between air pollutant and the specific cause.

†AN was calculated based on the largest effect estimates in single pollutant models; we set AN to zero when there is no significant association between air pollutant and the specific cause.

‡The unit of cost is million yuan.

§The unit of average total cost per HAs is yuan.

**‖**The unit of total costs is million yuan.

## DISCUSSION

This is one of the few studies to investigate the attributable risk and economic costs of HAs for CVDs due to PM_2.5_, PM_10_, and SO_2_ exposure in mainland China. The results indicated that PM_10_ and SO_2_ exposure was significantly associated with increased daily HAs for total CVDs, as well as specific causes of CVDs (cerebrovascular disease and ischaemic heart disease). The effects of PM_10_ and SO_2_ pollution on HAs for CVDs were more pronounced in cool seasons. Overall, 11.91% and 15.71% of HAs for CVDs were estimated to be attributed to PM_10_ and SO_2_. Besides, we found that air pollutants (including PM_10_ and SO_2_) entailed heavy economic burdens for CVDs hospitalization to society (144.34 million Yuan), accounting for approximately 0.028% of local GDP during the study period (2016-2020). To our knowledge, this is the first study to estimate the economic costs of HAs for CVDs due to SO_2_ exposure in mainland China, contributing to the limited scientific data on the effects of ambient air pollution on the morbidity of CVDs.

An increasing number of studies have suggested that PM negatively impacts human health, particularly the cardiovascular system [44,45]. Recently, epidemiological studies and clinical research have shown that ambient air pollution is associated with increased cardiovascular morbidity and mortality [46,47]. We found significant associations between PM_10_ and SO_2_ exposure and CVDs, consistent with previous studies. One study conducted in Iran showed a significant increase in cardiovascular HAs in the total population in relation to O_3_, NO_2_, NO, and SO_2_ [1]. Amsalu et al. [48] found that a 10 μg/m^3^ increase in the two-day average concentration (lag0-1) of SO_2_ was associated with an increase of 1.38% (95% CI = 0.99, 1.77%) in HAs for total CVDs. Nevertheless, we did not find significant effects of PM_2.5_ with CVDs admissions, which was different from other research results [29]. The reason may be related to variance in PM_2.5_ composition, toxicity, and concentration across different cities [49].

Several biological mechanisms have been identified which could be responsible for ambient air pollution-dependent adverse cardiovascular outcomes. First, ambient air pollutants may lead to the generation of endogenous pro-inflammatory mediators, oxidative stress, autonomic nervous system imbalance, endothelial dysfunction, and plasma viscosity increases [50-52], resulting in CVDs. Second, evidence on the biological mechanisms also indicates that the brain is a critical target for PM exposure and that PM pollution may contribute to the oxidative stress burden of the brain [53]. Third, ambient air pollution exposure may be associated with abnormal methylation levels of global DNA and specific genes involved in blood pressure regulation, glucose homeostasis, and lipid metabolism pathways [54]. Fourth, by directly affecting the autonomic nervous system, short-term exposure to SO_2_ reduces cardiac vagal control measures [55].

Global climate change has increased concerns about the interactive effects of temperature and air pollution on human health. Findings from previous studies suggests that the short-term PM_2.5_ effect on CVD hospitalizations varies with season and temperature levels, and stronger effects were observed in winter and at low-temperature days [56]. However, we observed that the effects of PM_10_ and SO_2_ pollution on CVDs were more evident in cool seasons than in warm seasons. The results were generally consistent with the heterogeneous effects observed by Duan [57]. One possible reason for the differences is the higher level of concentration of ambient air pollutants in the cool season than in the warm season [58]. However, season and temperature can strongly modify the adverse effects of ambient air pollutants on CVDs [23]. Duan et al. [57] found that in the cold season and on days with low temperatures, the adverse effect of NO_2_ on CVDs was significantly enhanced.

It was reported that ambient air pollution is significantly associated with cerebrovascular disease [59]. A systematic review and meta-analysis showed that increased PM_10_ concentrations were associated with HAs for stroke [60] and provided evidence linking air pollution to cerebrovascular diseases. There is a growing body of evidence that ambient air pollution increases the risk of cerebrovascular diseases through the induction of inflammation and oxidative stress [59]. We found significant effects of PM_10_ and SO_2_ exposure on HAs for ischaemic heart disease. Previous studies have suggested an increased risk of ischaemic heart disease related to air pollution. So et al. [61] investigated the effects of air pollutants on the risk of ischaemic heart disease admissions in South Korea and reported that one-month exposure to SO_2_ was related to 1.36-fold higher odds for ischaemic heart disease and one-year exposure to SO_2_ and PM_10_ was associated with 1.58 and 1.14-fold higher odds for ischaemic heart disease. One study conducted in China [62] also observed significant and positive correlations between admissions for ischaemic heart disease and short-term exposure to air pollutants.

We estimated the burden of HAs for CVDs attributable to ambient air pollutants exposure, which is of great value to policymaking and chronic disease prevention. We observed each 10μg/m3 increase in the PM_10_ and SO_2_ exposure was associated with an average annual increase in CVDs of about 3294 and 4346 HAs, which may form a heavy economic burden to both the city's medical insurance system and the patients' families. This study provided insight into the impact of ambient air pollution on the city and strengthened the necessity to make air pollution-related health intervention part of disease control and prevention.

When it comes to economic losses, previous studies have measured the economic costs of CVDs from air pollution (including PM_2.5_ and PM_10_) [27,29,30] and found that air pollutants entailed a heavy economic burden for CVDs hospitalization to society, which was consistent with our findings. Meanwhile, for the first time, our study provided straightforward information for assessing the economic burden of diseases caused by SO_2_ exposure by estimating economic costs regarding hospitalization expenses attributable to exceeding SO_2_ exposure. Further studies on PM_10_ and SO_2_ exposure with a longer time are needed to describe the time trends of the economic burden due to extra PM_10_ and SO_2_ exposure and to track the PM_10_ and SO_2_-related control benefits.

This study has several strengths. First, to our knowledge, it is the first to estimate the economic costs of HAs for CVDs due to SO_2_ exposure in mainland China. Second, we collected data on HAs for CVDs from all tertiary and secondary hospitals in the study area to avoid selection bias. Third, we conducted subgroup analyses to detect the susceptible population and season-specific effect estimates, which improved our understanding of the adverse effects of air pollutants on cardiovascular health.

Some limitations of our study should be mentioned. First, we only considered ambient air pollutants exposure, but not indoor air pollutants exposure, which may limit our ability to extrapolate our research. Second, there may be measurement errors due to assuming that residents in Panzhihua shared the same levels of ambient air pollutants exposure. Third, personal characteristics such as dietary habits and physical activity were not included in our study, which may affect our results. Fourth, wood stove exposure is an important risk factor. Due to data availability limitations, we cannot explore the exposure-response associations stratified by urban and rural areas to evaluate the effect of wood stove exposure. Fifth, in estimating economic losses, we calculated direct costs only by considering medical expenses incurred while a patient was hospitalized, as information on other direct costs (such as transportation) was not available. Thus, the direct cost of hospitalization for CVDs patients may be underestimated. Sixth, due to the lack of access to related information, we have not considered productivity losses related to time spent by family and friends visiting the admitted patients, which may have caused us to underestimate the associated productivity losses. Seventh, due to a lack of information on prevailing labour-force participation rates, we had to assume a full employment scenario when calculating productivity loss, which may make our estimates of productivity losses less accurate. Eighth, using GDP per capita as a measure of productivity loss per day may not allow for adequate accuracy. Ninth, the additivity of GAM restricts the impact of various important interactions in the model [63], which is a limitation inherent in the GAM. Thus, the interaction between variables in this study has not been fully considered. Finally, the limitations of the COI cannot be ignored. For example, COI assumes that people assume that diseases are exogenous and do not realize that they can take preventive measures, which ignores the cost of preventing disease [64,65]. Further studies should elucidate the effects of ambient air pollution and indoor air pollution on HAs for CVDs, as well as identify the factors which affect the susceptibility to air pollution, such as dietary habits, physical activity, and socioeconomic status, which will help strengthen our findings.

## CONCLUSIONS

We found the short-term effects of ambient air pollutants (PM_10_, SO_2_) on the risk of daily HAs for total CVDs and on specific disorders (cerebrovascular diseases and ischaemic heart disease). Additionally, substantial morbidity and economic burden of CVDs could be attributable to exceeding PM_10_ and SO_2_ exposure. Our findings supplement the limited evidence concerning the morbidity and economic burden attributed to air pollution on cardiovascular health in low-pollution areas of LMICs. More studies are needed to provide important scientific literature for understanding cardiovascular health risks concerning air pollutants exposure and formulating social and environmental policies for CVDs prevention.

## Additional material


Online Supplementary Document
