# Supplementary material for: Health and economic impacts of ambient air pollution on hospital admissions for overall and specific cardiovascular diseases in Panzhihua, Southwestern China
Source: J Glob Health. 2022 Dec 21;12:11012. doi: 10.7189/jogh.12.11012 (PMC9805700; doi:10.7189/jogh.12.11012)
Supplement: Online Supplementary Document [file jogh-12-11012-s001.pdf]

# ONLINE SUPPLEMENTARY DOCUMENT

**Title:** Health and economic impacts of ambient air pollution on hospital admissions for overall and specific cardiovascular diseases in Panzhihua, Southwestern China

**Authors:** Xianzhi Li, Yajie Li, Bin Yu, Hongwei Zhu, Zonglei Zhou, Yan Yang, Shunjin Liu, Yunyun Tian, Junjie Xiao, Xiangyi Xing, Li Yin

**Figure S1:** Selection of degrees of freedom for time trend.

**Figure S2:** Heatmap of Spearman's correlation coefficient between the daily levels of air pollutants and meteorological variables.

**Table S1:** Excess risk (95% CI) in hospital admissions for cardiovascular disorders associated with a 10 μg/m$^3$ in $PM_{2.5}$, $PM_{10}$, and $SO_2$, along different lag days using single-pollutant and two-pollutant models.

**Table S2:** Excess risk (95% CI) in hospital admissions for cardiovascular disorders associated with 10 μg/m$^3$ increase in $PM_{2.5}$, $PM_{10}$, and $SO_2$ at lag06 under varying degrees of freedom for the smooth function of time trend using single-pollutant models.

**Table S3:** Excess risk (95% CI) of air pollution on cardiovascular disorders in the original model and the adjusted model.

**Table S4:** Mean treatment cost per hospital admission ($C_h$), and the mean hospitalization days per hospital admission ($meanT_h$) by cause of study population, and daily GDP per capita (*dGDPp*) during 2016-2020 in Panzhihua.

**Table S5 :**The largest effect estimates ($\beta$) and standard error (*SE*) between air pollutants and overall and specific cardiovascular diseases in single pollutant models.

**Table S6 :**The baseline patients due to ambient air pollutants, and difference in daily air pollutants concentration with the threshold level ($\Delta Y$) in Panzhihua, 2016-2021.

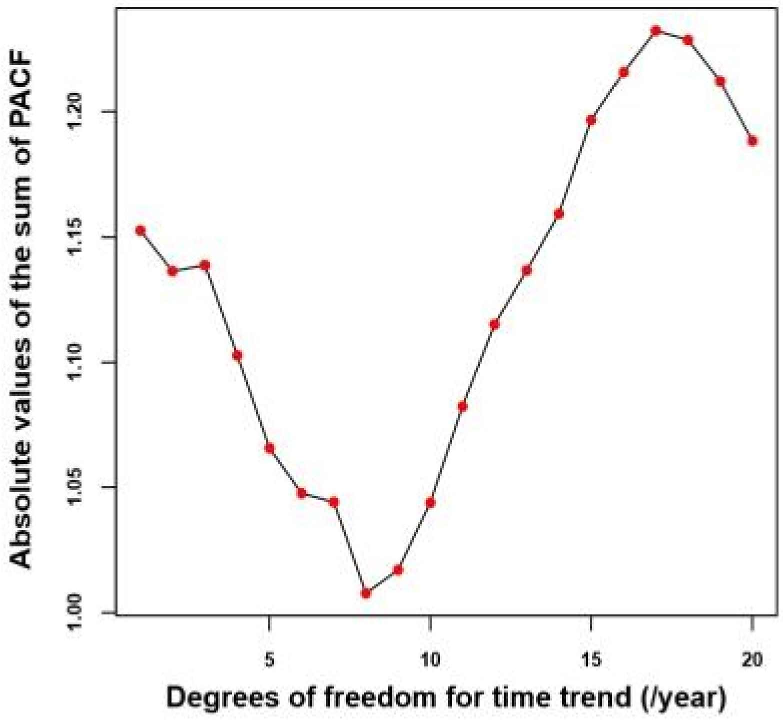


**Figure S1** Selection of degrees of freedom for time trend. ***Notes :*** PACF, partial autocorrelation function

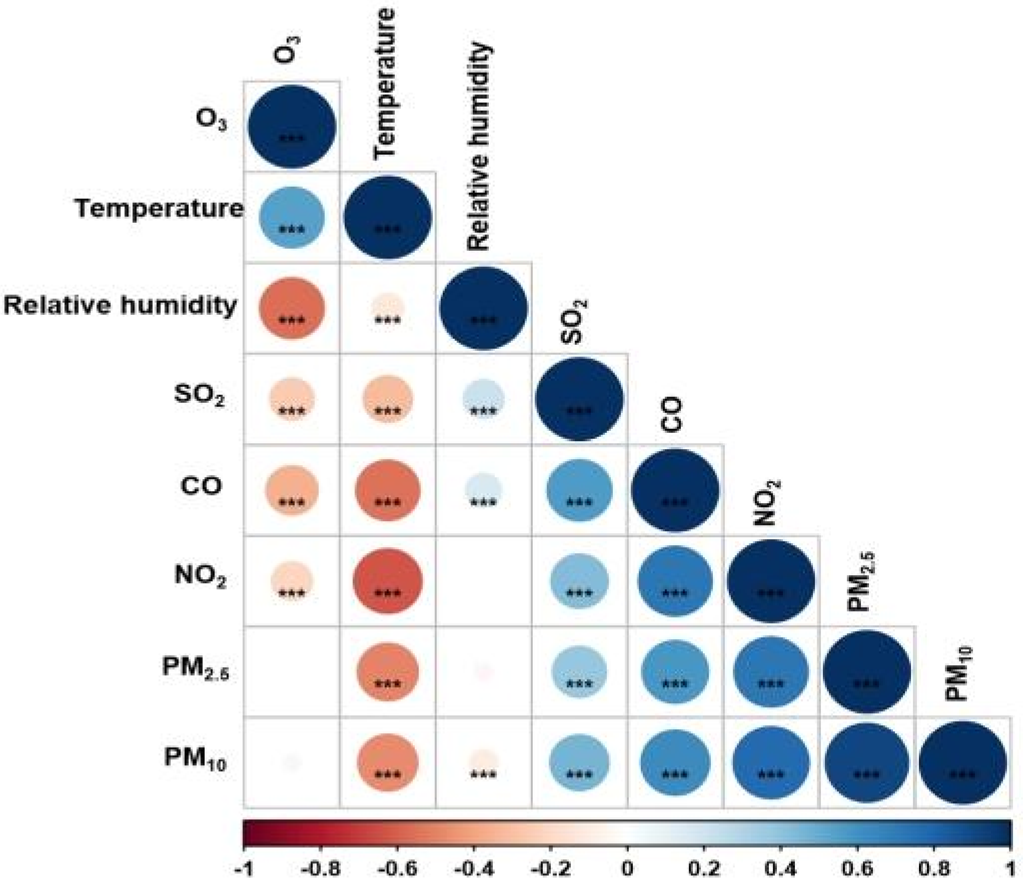


**Figure S2** Heatmap of Spearman's correlation coefficient between the daily levels of air pollutants and meteorological variables in Panzhihua, 2016-2020. ***Notes :*** *** $P < 0.001$; the size of the circle and the depth of the color represent the values of the correlation coefficient.

**Table S1** Excess risk (95% CI) in hospital admissions for cardiovascular disorders associated with a 10 μg/m$^3$ in $PM_{2.5}$, $PM_{10}$, and $SO_2$, along different lag days using single-pollutant and two-pollutant models

| | **Lag0** | **Lag1** | **Lag2** | **Lag3** | **Lag4** | **Lag5** | **Lag6** |
|---|---|---|---|---|---|---|---|
| **$PM_{2.5}$** | 0.89(-0.80,2.61) | 0.30(-1.39,2.03) | 0.83(-0.92,2.60) | 0.62(-1.13,2.40) | 0.74(-1.02,2.53) | 0.79(-0.98,2.59) | 1.15(-0.64,2.97) |
| +$SO_2$ | -0.43(-2.23,1.41) | -0.24(-1.95,1.50) | 0.60(-1.14,2.37) | 0.49(-1.26,2.26) | 0.61(-1.15,2.40) | 0.56(-1.21,2.36) | 0.78(-1.00,2.60) |
| +$NO_2$ | -0.07(-2.20,2.11) | -0.35(-2.19,1.52) | 0.50(-1.28,2.32) | 0.36(-1.41,2.17) | 0.50(-1.29,2.31) | 0.55(-1.25,2.38) | 0.96(-0.85,2.80) |
| +CO | 1.18(-0.74,3.14) | 0.36(-1.43,2.18) | 0.87(-0.91,2.68) | 0.64(-1.12,2.44) | 0.76(-1.02,2.57) | 0.81(-0.98,2.64) | 1.19(-0.62,3.04) |
| +$O_3$ | 1.48(-0.33,3.33) | 0.51(-1.21,2.27) | 0.97(-0.78,2.76) | 0.76(-1.00,2.55) | 0.86(-0.91,2.66) | 0.89(-0.89,2.71) | 1.25(-0.54,3.08) |
| **$PM_{10}$** | **1.43(0.36,2.52)** | **1.28(0.21,2.37)** | **1.32(0.22,2.43)** | 1.00(-0.11,2.11) | 1.15(-0.04,2.28) | 0.90(-0.21,2.02) | 0.92(-0.19,2.04) |
| +$SO_2$ | 0.59(-0.60,1.80) | 0.88(-0.21,1.99) | **1.09(0.00,2.21)** | 0.81(-0.29,1.93) | 0.97(-0.14,2.10) | 0.68(-0.43,1.81) | 0.58(-0.53,1.71) |
| +$NO_2$ | **1.72(0.18,3.28)** | 1.07(-0.14,2.29) | 1.13(-0.01,2.29) | 0.83(-0.30,1.96) | 1.00(-0.14,2.15) | 0.74(-0.40,1.89) | 0.76(-0.36,1.89) |
| +CO | **2.04(0.76,3.34)** | **1.46(0.31,2.62)** | **1.38(0.26,2.52)** | 1.02(-0.09,2.14) | 1.17(-0.05,2.31) | 0.92(-0.20,2.06) | 0.94(-0.18,2.07) |

| | | | | | | | |
|---|---|---|---|---|---|---|---|
| +$O_3$ | **1.76(0.65,2.87)** | **1.35(0.27,2.44)** | **1.36(0.26,2.46)** | 1.04(-0.06,2.15) | 1.20(-0.08,2.33) | 0.94(-0.18,2.06) | 0.96(-0.14,2.08) |
| **$SO_2$** | **2.74(1.32,4.18)** | **2.59(1.19,4.01)** | **1.88(0.52,3.26)** | **1.56(0.19,2.95)** | **1.62(0.23,3.03)** | **1.49(0.09,2.9)** | 1.25(-0.12,2.63) |
| +$PM_{2.5}$ | **2.88(1.34,4.43)** | **2.54(1.12,3.98)** | **1.84(0.48,3.22)** | **1.53(0.16,2.93)** | **1.57(0.18,2.99)** | **1.43(0.04,2.85)** | 1.18(-0.18,2.57) |
| +$PM_{10}$ | **2.37(0.78,3.98)** | **2.28(0.84,3.73)** | **1.73(0.36,3.11)** | **1.44(0.07,2.84)** | **1.45(0.06,2.87)** | 1.30(-0.09,2.72) | 1.04(-0.33,2.42) |
| +$NO_2$ | **2.63(1.09,4.18)** | **2.44(1.01,3.89)** | **1.81(0.44,3.19)** | **1.52(0.15,2.92)** | **1.60(0.21,3.01)** | **1.43(0.03,2.84)** | 1.17(-0.19,2.56) |
| +CO | **3.76(2.10,5.44)** | **2.76(1.32,4.23)** | **1.91(0.55,3.30)** | **1.57(0.20,2.97)** | **1.63(0.24,3.04)** | **1.50(0.11,2.92)** | 1.27(-0.10,2.66) |
| +$O_3$ | **2.71(1.29,4.15)** | **2.51(1.11,3.94)** | **1.81(0.45,3.19)** | **1.51(0.14,2.90)** | **1.56(0.17,2.97)** | **1.43(0.03,2.84)** | 1.18(-0.18,2.56) |

***Notes:*** Bold fonts indicates the effect was statistically significant ($P$<0.05).

**Table S2** Excess risk (95% CI) in hospital admissions for cardiovascular disorders associated with 10 μg/m$^3$ increase in $PM_{2.5}$, $PM_{10}$, and $SO_2$ at lag06 under varying degrees of freedom for the smooth function of time trend using single-pollutant models

| **df** | **$PM_{2.5}$** | **$PM_{10}$** | **$SO_2$** |
|---|---|---|---|
| **6** | 1.86(-0.66,4.44) | **2.52(0.96,4.10)** | **4.27(1.99,6.61)** |
| **7** | 1.62(-0.90,4.21) | **2.48(0.92,4.06)** | **5.50(3.09,7.97)** |
| **8** | 0.66(-1.90,3.29) | **1.84(0.23,3.47)** | **4.81(2.33,7.35)** |
| **9** | -0.47(-3.09,2.22) | 1.29(-0.35,2.95) | **4.52(2.01,7.09)** |
| **10** | -0.76(-3.37,1.91) | 0.76(-0.88,2.43) | **4.19(1.69,6.75)** |

***Notes:*** Bold fonts indicates the effect was statistically significant (*P*<0.05).

**Table S3** Excess risk (95% CI) of air pollution on cardiovascular disorders in the original model and the adjusted model

| | $PM_{2.5}$ | | $PM_{10}$ | | $SO_2$ | |
|---|---|---|---|---|---|---|
| | **Original *** | **Adjusted †** | **Original *** | **Adjusted †** | **Original *** | **Adjusted †** |
| **lag0** | 0.89<br>(-0.80,2.61) | 1.09<br>(-0.70,2.92) | **1.43**<br>**(0.36,2.52)** | **1.73**<br>**(0.59,2.87)** | **2.74**<br>**(1.32,4.18)** | **2.90**<br>**(1.44,4.37)** |
| **lag1** | 0.30<br>(-1.39,2.03) | 0.33<br>(-1.37,2.07) | **1.28**<br>**(0.21,2.37)** | **1.30**<br>**(0.22,2.4)** | **2.59**<br>**(1.19,4.01)** | **2.61**<br>**(1.21,4.04)** |
| **lag2** | 0.83<br>(-0.92,2.60) | 0.84<br>(-0.91,2.61) | **1.32**<br>**(0.22,2.43)** | **1.32**<br>**(0.23,2.43)** | **1.88**<br>**(0.52,3.26)** | **1.89**<br>**(0.53,3.27)** |
| **lag3** | 0.62<br>(-1.13,2.40) | 0.63<br>(-1.12,2.41) | 1.00<br>(-0.11,2.11) | 1.00<br>(-0.11,2.11) | **1.56**<br>**(0.19,2.95)** | **1.56**<br>**(0.19,2.95)** |
| **lag4** | 0.74<br>(-1.02,2.53) | 0.75<br>(-1.01,2.55) | 1.15<br>(-0.04,2.28) | 1.16<br>(-0.04,2.29) | **1.62**<br>**(0.23,3.03)** | **1.62**<br>**(0.23,3.03)** |
| **lag5** | 0.79<br>(-0.98,2.59) | 0.80<br>(-0.98,2.60) | 0.90<br>(-0.21,2.02) | 0.90<br>(-0.21,2.03) | **1.49**<br>**(0.09,2.90)** | **1.50**<br>**(0.10,2.91)** |
| **lag6** | 1.15<br>(-0.64,2.97) | 1.16<br>(-0.63,2.98) | 0.92<br>(-0.19,2.04) | 0.92<br>(-0.19,2.04) | 1.25<br>(-0.12,2.63) | 1.27<br>(-0.10,2.65) |
| **lag01** | 0.73 | 0.84 | **1.72** | **1.86** | **3.88** | **4.03** |

| | | | | | | |
|---|---|---|---|---|---|---|
| | (-1.14,2.63) | (-1.08,2.81) | **(0.54,2.91)** | **(0.64,3.09)** | **(2.17,5.62)** | **(2.29,5.80)** |
| **lag02** | 0.96 | 1.06 | **1.98** | **2.07** | **4.42** | **4.54** |
| | (-1.07,3.04) | (-1.02,3.18) | **(0.69,3.27)** | **(0.76,3.39)** | **(2.51,6.36)** | **(2.61,6.51)** |
| **lag03** | 1.07 | 1.15 | **2.08** | **2.15** | **4.77** | **4.86** |
| | (-1.11,3.30) | (-1.06,3.42) | **(0.71,3.46)** | **(0.77,3.55)** | **(2.69,6.89)** | **(2.77,7.00)** |
| **lag04** | 1.19 | 1.27 | **2.26** | **2.32** | **5.15** | **5.23** |
| | (-1.12,3.56) | (-1.07,3.67) | **(0.82,3.72)** | **(0.87,3.79)** | **(2.93,7.42)** | **(3.00,7.51)** |
| **lag05** | 1.34 | 1.41 | **2.35** | **2.40** | **5.43** | **5.51** |
| | (-1.08,3.82) | (-1.03,3.92) | **(0.85,3.87)** | **(0.89,3.94)** | **(3.10,7.82)** | **(3.16,7.92)** |
| **lag06** | 1.62 | 1.70 | **2.48** | **2.53** | **5.50** | **5.59** |
| | (-0.90,4.21) | (-0.85,4.31) | **(0.92,4.06)** | **(0.95,4.12)** | **(3.09,7.97)** | **(3.16,8.07)** |

***Notes:*** *Single-pollutant models with controlling for time trend, relative humidity, and temperature in the models; ‡adding additional weather conditions (wind speed, and atmospheric pressure ) in the models; Bold fonts indicates the effect was statistically significant ($P$<0.05).

**Table S4:**

Mean treatment cost per hospital admission ($C_h$), and the mean hospitalization days per hospital admission ($meanT_h$) by cause of study population, and daily GDP per capita (*dGDPp*) during 2016-2020 in Panzhihua.

| **Cause** | **$C_h$ (Yuan)** | **$meanT_h$ (Days)** | ***dGDPp* (Yuan)** | **Average total cost per HAs** |
|---|---|---|---|---|
| **Cardiovascular disease** | 16120.80 | 12.05 | 230.45 | 18897.72 |
| **Cerebrovascular disease** | 17518.91 | 14.71 | 230.45 | 20908.83 |
| **Ischemic heart disease** | 17290.27 | 9.35 | 230.45 | 19444.98 |

*Notes:* HAs, hospital admissions; the unit of Average total cost per HAs is yuan.

**Table S5**

The largest effect estimates (*β*) and standard error (*SE*) between air pollutants and overall and specific cardiovascular diseases in single pollutant models.

| Cause | *B (SE)* | | |
|---|---|---|---|
| | **$PM_{2.5}$** | **$PM_{10}$** | **$SO_2$** |
| **Cardiovascular disease** | NS* | 0.0025(0.00078) | 0.0054(0.00118) |
| **Cerebrovascular disease** | NS* | 0.0017(0.00074) | 0.0043(0.00147) |
| **Ischemic heart disease** | NS* | 0.0034(0.00122) | 0.0063(0.00170) |

***Notes:*** *NS, No statistical significance.

**Table S6**

The baseline patients due to ambient air pollutants and difference in daily air pollutants concentration with the threshold level (*ΔY*) in Panzhihua, 2016-2021.

| time | date | *baseline patients (n)* | | | *ΔY (μg/m³)* | | |
|---|---|---|---|---|---|---|---|
| | | **Cardiovascular disease** | **Cerebrovascular disease** | **Ischemic heart disease** | **$PM_{2.5}$** | **$PM_{10}$** | **$SO_2$** |
| 1 | 2016-1-1 | 7 | 3 | 3 | 42 | 79 | 31 |
| 2 | 2016-1-2 | 4 | 1 | 2 | 28 | 57 | 32 |
| 3 | 2016-1-3 | 6 | 1 | 2 | 28 | 63 | 27 |
| 4 | 2016-1-4 | 24 | 6 | 2 | 29 | 75 | 28 |
| 5 | 2016-1-5 | 20 | 4 | 7 | 40 | 86 | 30 |
| 6 | 2016-1-6 | 26 | 9 | 5 | 41 | 86 | 30 |
| 7 | 2016-1-7 | 20 | 6 | 6 | 42 | 88 | 31 |
| 8 | 2016-1-8 | 11 | 3 | 3 | 39 | 80 | 24 |
| 9 | 2016-1-9 | 3 | 0 | 0 | 39 | 89 | 36 |
| 10 | 2016-1-10 | 7 | 3 | 3 | 35 | 76 | 34 |
| 11 | 2016-1-11 | 29 | 4 | 14 | 37 | 81 | 33 |
| 12 | 2016-1-12 | 19 | 3 | 2 | 45 | 91 | 43 |
| 13 | 2016-1-13 | 20 | 7 | 2 | 44 | 87 | 47 |
| 14 | 2016-1-14 | 19 | 5 | 6 | 53 | 104 | 75 |
| 15 | 2016-1-15 | 6 | 4 | 1 | 37 | 73 | 31 |
| 16 | 2016-1-16 | 8 | 3 | 2 | 42 | 84 | 69 |
| 17 | 2016-1-17 | 9 | 2 | 4 | 39 | 77 | 33 |
| 18 | 2016-1-18 | 33 | 8 | 11 | 51 | 94 | 41 |
| 19 | 2016-1-19 | 18 | 6 | 5 | 49 | 94 | 64 |
| 20 | 2016-1-20 | 18 | 7 | 8 | 41 | 89 | 55 |
| 21 | 2016-1-21 | 17 | 7 | 1 | 27 | 60 | 18 |
| 22 | 2016-1-22 | 9 | 4 | 0 | 21 | 40 | 15 |
| 23 | 2016-1-23 | 7 | 2 | 2 | 22 | 48 | 32 |
| 24 | 2016-1-24 | 7 | 4 | 1 | 20 | 44 | 20 |

| | | | | | | | |
|---|---|---|---|---|---|---|---|
| 25 | 2016-1-25 | 21 | 8 | 2 | 38 | 52 | 12 |
| 26 | 2016-1-26 | 11 | 4 | 5 | 47 | 69 | 33 |
| 27 | 2016-1-27 | 12 | 4 | 3 | 49 | 78 | 46 |
| 28 | 2016-1-28 | 16 | 5 | 3 | 22 | 41 | 25 |
| 29 | 2016-1-29 | 14 | 4 | 1 | 28 | 55 | 23 |
| 30 | 2016-1-30 | 11 | 4 | 1 | 34 | 69 | 39 |
| 31 | 2016-1-31 | 8 | 4 | 2 | 36 | 65 | 42 |
| 32 | 2016-2-1 | 17 | 0 | 6 | 23 | 46 | 41 |
| 33 | 2016-2-2 | 10 | 3 | 3 | 33 | 55 | 64 |
| 34 | 2016-2-3 | 3 | 2 | 0 | 49 | 84 | 37 |
| 35 | 2016-2-4 | 7 | 5 | 0 | 35 | 63 | 23 |
| 36 | 2016-2-5 | 6 | 3 | 0 | 35 | 57 | 32 |
| 37 | 2016-2-6 | 4 | 2 | 1 | 25 | 39 | 16 |
| 38 | 2016-2-7 | 7 | 3 | 1 | 33 | 54 | 27 |
| 39 | 2016-2-8 | 6 | 2 | 1 | 60 | 87 | 37 |
| 40 | 2016-2-9 | 5 | 1 | 1 | 29 | 46 | 25 |
| 41 | 2016-2-10 | 8 | 1 | 1 | 16 | 35 | 24 |
| 42 | 2016-2-11 | 10 | 4 | 3 | 19 | 37 | 23 |
| 43 | 2016-2-12 | 7 | 3 | 3 | 17 | 36 | 21 |
| 44 | 2016-2-13 | 16 | 5 | 2 | 21 | 37 | 14 |
| 45 | 2016-2-14 | 25 | 7 | 6 | 17 | 34 | 16 |
| 46 | 2016-2-15 | 35 | 7 | 8 | 20 | 54 | 21 |
| 47 | 2016-2-16 | 23 | 11 | 7 | 31 | 63 | 32 |
| 48 | 2016-2-17 | 19 | 4 | 5 | 30 | 60 | 23 |
| 49 | 2016-2-18 | 18 | 3 | 5 | 35 | 70 | 23 |
| 50 | 2016-2-19 | 14 | 6 | 3 | 35 | 68 | 19 |
| 51 | 2016-2-20 | 7 | 4 | 1 | 28 | 51 | 16 |
| 52 | 2016-2-21 | 6 | 3 | 2 | 36 | 66 | 54 |
| 53 | 2016-2-22 | 20 | 9 | 3 | 24 | 53 | 35 |
| 54 | 2016-2-23 | 20 | 4 | 4 | 33 | 77 | 42 |
| 55 | 2016-2-24 | 16 | 7 | 1 | 15 | 34 | 25 |
| 56 | 2016-2-25 | 16 | 4 | 6 | 24 | 39 | 41 |

| | | | | | | | |
|---|---|---|---|---|---|---|---|
| 57 | 2016-2-26 | 18 | 4 | 2 | 19 | 33 | 26 |
| 58 | 2016-2-27 | 13 | 6 | 3 | 29 | 56 | 47 |
| 59 | 2016-2-28 | 12 | 4 | 2 | 28 | 54 | 46 |
| 60 | 2016-2-29 | 28 | 10 | 9 | 31 | 63 | 35 |
| 61 | 2016-3-1 | 20 | 3 | 5 | 22 | 38 | 25 |
| 62 | 2016-3-2 | 21 | 7 | 2 | 22 | 47 | 20 |
| 63 | 2016-3-3 | 14 | 6 | 2 | 24 | 55 | 28 |
| 64 | 2016-3-4 | 13 | 1 | 1 | 32 | 65 | 33 |
| 65 | 2016-3-5 | 6 | 3 | 0 | 31 | 62 | 26 |
| 66 | 2016-3-6 | 9 | 1 | 3 | 29 | 59 | 37 |
| 67 | 2016-3-7 | 25 | 5 | 7 | 29 | 62 | 26 |
| 68 | 2016-3-8 | 24 | 9 | 8 | 19 | 44 | 18 |
| 69 | 2016-3-9 | 20 | 6 | 4 | 25 | 57 | 29 |
| 70 | 2016-3-10 | 21 | 9 | 4 | 23 | 52 | 36 |
| 71 | 2016-3-11 | 19 | 5 | 2 | 27 | 62 | 33 |
| 72 | 2016-3-12 | 9 | 6 | 1 | 26 | 58 | 27 |
| 73 | 2016-3-13 | 7 | 5 | 1 | 26 | 52 | 31 |
| 74 | 2016-3-14 | 24 | 3 | 6 | 26 | 55 | 22 |
| 75 | 2016-3-15 | 19 | 4 | 5 | 19 | 42 | 25 |
| 76 | 2016-3-16 | 18 | 6 | 3 | 22 | 44 | 18 |
| 77 | 2016-3-17 | 22 | 7 | 7 | 23 | 43 | 16 |
| 78 | 2016-3-18 | 24 | 7 | 8 | 26 | 55 | 26 |
| 79 | 2016-3-19 | 5 | 0 | 0 | 37 | 75 | 26 |
| 80 | 2016-3-20 | 7 | 2 | 4 | 28 | 50 | 16 |
| 81 | 2016-3-21 | 29 | 8 | 8 | 26 | 49 | 16 |
| 82 | 2016-3-22 | 12 | 2 | 1 | 32 | 59 | 33 |
| 83 | 2016-3-23 | 16 | 7 | 4 | 24 | 58 | 24 |
| 84 | 2016-3-24 | 11 | 4 | 1 | 10 | 23 | 18 |
| 85 | 2016-3-25 | 10 | 3 | 3 | 22 | 44 | 32 |
| 86 | 2016-3-26 | 15 | 5 | 3 | 29 | 61 | 41 |
| 87 | 2016-3-27 | 8 | 1 | 3 | 37 | 68 | 36 |
| 88 | 2016-3-28 | 26 | 6 | 10 | 56 | 95 | 45 |

| | | | | | | | |
|---|---|---|---|---|---|---|---|
| 89 | 2016-3-29 | 9 | 3 | 3 | 44 | 75 | 22 |
| 90 | 2016-3-30 | 14 | 5 | 2 | 19 | 39 | 29 |
| 91 | 2016-3-31 | 20 | 7 | 2 | 23 | 49 | 22 |
| 92 | 2016-4-1 | 13 | 4 | 5 | 14 | 30 | 19 |
| 93 | 2016-4-2 | 4 | 2 | 0 | 18 | 42 | 16 |
| 94 | 2016-4-3 | 6 | 3 | 2 | 19 | 43 | 28 |
| 95 | 2016-4-4 | 17 | 5 | 6 | 21 | 53 | 25 |
| 96 | 2016-4-5 | 34 | 11 | 9 | 22 | 45 | 14 |
| 97 | 2016-4-6 | 14 | 5 | 2 | 19 | 38 | 26 |
| 98 | 2016-4-7 | 29 | 9 | 9 | 20 | 46 | 41 |
| 99 | 2016-4-8 | 11 | 2 | 4 | 27 | 55 | 36 |
| 100 | 2016-4-9 | 12 | 4 | 2 | 25 | 69 | 38 |
| 101 | 2016-4-10 | 12 | 4 | 6 | 20 | 51 | 25 |
| 102 | 2016-4-11 | 33 | 13 | 7 | 27 | 58 | 39 |
| 103 | 2016-4-12 | 20 | 9 | 2 | 30 | 66 | 24 |
| 104 | 2016-4-13 | 30 | 7 | 8 | 29 | 73 | 45 |
| 105 | 2016-4-14 | 23 | 8 | 4 | 22 | 52 | 38 |
| 106 | 2016-4-15 | 11 | 3 | 3 | 18 | 44 | 26 |
| 107 | 2016-4-16 | 10 | 5 | 3 | 28 | 62 | 45 |
| 108 | 2016-4-17 | 5 | 1 | 2 | 28 | 68 | 29 |
| 109 | 2016-4-18 | 23 | 8 | 7 | 22 | 50 | 42 |
| 110 | 2016-4-19 | 14 | 3 | 1 | 25 | 52 | 35 |
| 111 | 2016-4-20 | 23 | 10 | 7 | 20 | 42 | 31 |
| 112 | 2016-4-21 | 18 | 7 | 6 | 30 | 49 | 28 |
| 113 | 2016-4-22 | 13 | 2 | 4 | 24 | 49 | 34 |
| 114 | 2016-4-23 | 10 | 5 | 2 | 20 | 44 | 35 |
| 115 | 2016-4-24 | 10 | 2 | 4 | 25 | 49 | 42 |
| 116 | 2016-4-25 | 23 | 7 | 5 | 24 | 57 | 45 |
| 117 | 2016-4-26 | 12 | 2 | 1 | 21 | 52 | 27 |
| 118 | 2016-4-27 | 23 | 10 | 5 | 25 | 54 | 26 |
| 119 | 2016-4-28 | 9 | 3 | 2 | 33 | 70 | 27 |
| 120 | 2016-4-29 | 16 | 1 | 8 | 26 | 52 | 35 |

| | | | | | | | |
|---|---|---|---|---|---|---|---|
| 121 | 2016-4-30 | 8 | 4 | 2 | 32 | 71 | 37 |
| 122 | 2016-5-1 | 3 | 0 | 2 | 27 | 57 | 39 |
| 123 | 2016-5-2 | 11 | 2 | 6 | 36 | 63 | 38 |
| 124 | 2016-5-3 | 20 | 6 | 2 | 38 | 77 | 42 |
| 125 | 2016-5-4 | 21 | 8 | 3 | 19 | 36 | 21 |
| 126 | 2016-5-5 | 13 | 3 | 2 | 26 | 50 | 27 |
| 127 | 2016-5-6 | 13 | 6 | 3 | 30 | 56 | 20 |
| 128 | 2016-5-7 | 6 | 2 | 4 | 20 | 35 | 19 |
| 129 | 2016-5-8 | 14 | 8 | 1 | 19 | 41 | 15 |
| 130 | 2016-5-9 | 17 | 3 | 6 | 18 | 41 | 23 |
| 131 | 2016-5-10 | 15 | 6 | 1 | 24 | 45 | 24 |
| 132 | 2016-5-11 | 17 | 8 | 2 | 28 | 51 | 30 |
| 133 | 2016-5-12 | 16 | 6 | 3 | 32 | 62 | 47 |
| 134 | 2016-5-13 | 18 | 9 | 3 | 26 | 49 | 32 |
| 135 | 2016-5-14 | 10 | 3 | 2 | 20 | 39 | 22 |
| 136 | 2016-5-15 | 8 | 5 | 3 | 14 | 31 | 23 |
| 137 | 2016-5-16 | 24 | 7 | 6 | 23 | 42 | 33 |
| 138 | 2016-5-17 | 27 | 6 | 9 | 26 | 51 | 40 |
| 139 | 2016-5-18 | 15 | 2 | 6 | 18 | 40 | 18 |
| 140 | 2016-5-19 | 12 | 2 | 3 | 13 | 28 | 19 |
| 141 | 2016-5-20 | 18 | 6 | 7 | 15 | 32 | 36 |
| 142 | 2016-5-21 | 3 | 2 | 0 | 25 | 49 | 51 |
| 143 | 2016-5-22 | 4 | 0 | 3 | 21 | 32 | 40 |
| 144 | 2016-5-23 | 27 | 10 | 2 | 12 | 21 | 44 |
| 145 | 2016-5-24 | 24 | 7 | 4 | 18 | 28 | 32 |
| 146 | 2016-5-25 | 17 | 4 | 4 | 18 | 34 | 31 |
| 147 | 2016-5-26 | 12 | 3 | 5 | 13 | 29 | 23 |
| 148 | 2016-5-27 | 11 | 1 | 3 | 17 | 38 | 22 |
| 149 | 2016-5-28 | 9 | 2 | 5 | 18 | 40 | 15 |
| 150 | 2016-5-29 | 9 | 2 | 2 | 19 | 43 | 24 |
| 151 | 2016-5-30 | 32 | 9 | 7 | 24 | 53 | 19 |
| 152 | 2016-5-31 | 11 | 7 | 1 | 15 | 33 | 9 |

| | | | | | | | |
|---|---|---|---|---|---|---|---|
| 153 | 2016-6-1 | 14 | 4 | 1 | 14 | 31 | 14 |
| 154 | 2016-6-2 | 16 | 6 | 2 | 10 | 24 | 18 |
| 155 | 2016-6-3 | 8 | 3 | 2 | 9 | 24 | 20 |
| 156 | 2016-6-4 | 3 | 3 | 0 | 16 | 37 | 31 |
| 157 | 2016-6-5 | 6 | 2 | 0 | 17 | 32 | 28 |
| 158 | 2016-6-6 | 25 | 6 | 9 | 23 | 43 | 39 |
| 159 | 2016-6-7 | 15 | 4 | 3 | 16 | 32 | 27 |
| 160 | 2016-6-8 | 17 | 7 | 5 | 16 | 32 | 44 |
| 161 | 2016-6-9 | 7 | 3 | 2 | 16 | 30 | 28 |
| 162 | 2016-6-10 | 7 | 0 | 3 | 13 | 27 | 28 |
| 163 | 2016-6-11 | 8 | 5 | 2 | 16 | 25 | 26 |
| 164 | 2016-6-12 | 14 | 6 | 2 | 18 | 34 | 31 |
| 165 | 2016-6-13 | 20 | 6 | 6 | 13 | 23 | 25 |
| 166 | 2016-6-14 | 26 | 8 | 6 | 13 | 27 | 20 |
| 167 | 2016-6-15 | 13 | 3 | 3 | 12 | 27 | 17 |
| 168 | 2016-6-16 | 18 | 4 | 6 | 11 | 19 | 24 |
| 169 | 2016-6-17 | 13 | 6 | 2 | 15 | 33 | 50 |
| 170 | 2016-6-18 | 10 | 3 | 5 | 10 | 25 | 27 |
| 171 | 2016-6-19 | 3 | 1 | 1 | 9 | 22 | 27 |
| 172 | 2016-6-20 | 22 | 7 | 4 | 11 | 23 | 43 |
| 173 | 2016-6-21 | 22 | 6 | 7 | 16 | 34 | 31 |
| 174 | 2016-6-22 | 23 | 11 | 3 | 17 | 29 | 23 |
| 175 | 2016-6-23 | 17 | 5 | 3 | 12 | 27 | 16 |
| 176 | 2016-6-24 | 16 | 8 | 3 | 9 | 24 | 17 |
| 177 | 2016-6-25 | 6 | 4 | 0 | 14 | 34 | 52 |
| 178 | 2016-6-26 | 7 | 5 | 1 | 15 | 36 | 32 |
| 179 | 2016-6-27 | 24 | 5 | 3 | 10 | 25 | 15 |
| 180 | 2016-6-28 | 26 | 9 | 3 | 15 | 36 | 25 |
| 181 | 2016-6-29 | 13 | 5 | 3 | 18 | 31 | 36 |
| 182 | 2016-6-30 | 15 | 8 | 2 | 22 | 45 | 35 |
| 183 | 2016-7-1 | 11 | 4 | 1 | 10 | 21 | 21 |
| 184 | 2016-7-2 | 6 | 3 | 0 | 27 | 54 | 38 |

| | | | | | | | |
|---|---|---|---|---|---|---|---|
| 185 | 2016-7-3 | 5 | 0 | 2 | 26 | 45 | 36 |
| 186 | 2016-7-4 | 22 | 5 | 9 | 12 | 30 | 24 |
| 187 | 2016-7-5 | 21 | 9 | 5 | 13 | 25 | 20 |
| 188 | 2016-7-6 | 9 | 4 | 3 | 16 | 28 | 37 |
| 189 | 2016-7-7 | 22 | 1 | 9 | 18 | 30 | 28 |
| 190 | 2016-7-8 | 16 | 2 | 2 | 20 | 41 | 35 |
| 191 | 2016-7-9 | 11 | 2 | 5 | 16 | 46 | 22 |
| 192 | 2016-7-10 | 6 | 0 | 2 | 16 | 41 | 32 |
| 193 | 2016-7-11 | 28 | 13 | 1 | 16 | 34 | 34 |
| 194 | 2016-7-12 | 25 | 12 | 1 | 18 | 36 | 24 |
| 195 | 2016-7-13 | 13 | 3 | 2 | 19 | 43 | 28 |
| 196 | 2016-7-14 | 17 | 5 | 6 | 19 | 38 | 24 |
| 197 | 2016-7-15 | 9 | 2 | 4 | 7 | 14 | 15 |
| 198 | 2016-7-16 | 11 | 6 | 1 | 19 | 40 | 37 |
| 199 | 2016-7-17 | 7 | 4 | 2 | 13 | 32 | 33 |
| 200 | 2016-7-18 | 26 | 7 | 5 | 13 | 36 | 30 |
| 201 | 2016-7-19 | 20 | 3 | 9 | 16 | 33 | 63 |
| 202 | 2016-7-20 | 18 | 6 | 6 | 18 | 39 | 84 |
| 203 | 2016-7-21 | 13 | 4 | 1 | 12 | 30 | 34 |
| 204 | 2016-7-22 | 12 | 4 | 3 | 8 | 24 | 31 |
| 205 | 2016-7-23 | 11 | 6 | 1 | 11 | 30 | 42 |
| 206 | 2016-7-24 | 11 | 5 | 2 | 11 | 26 | 39 |
| 207 | 2016-7-25 | 34 | 8 | 6 | 12 | 31 | 68 |
| 208 | 2016-7-26 | 23 | 4 | 4 | 20 | 42 | 31 |
| 209 | 2016-7-27 | 22 | 6 | 6 | 24 | 53 | 61 |
| 210 | 2016-7-28 | 10 | 2 | 2 | 24 | 39 | 38 |
| 211 | 2016-7-29 | 10 | 1 | 3 | 20 | 33 | 52 |
| 212 | 2016-7-30 | 7 | 3 | 1 | 22 | 45 | 37 |
| 213 | 2016-7-31 | 6 | 4 | 0 | 23 | 46 | 54 |
| 214 | 2016-8-1 | 23 | 7 | 4 | 15 | 29 | 29 |
| 215 | 2016-8-2 | 23 | 7 | 6 | 21 | 41 | 34 |
| 216 | 2016-8-3 | 15 | 6 | 3 | 19 | 36 | 47 |

| | | | | | | | |
|---|---|---|---|---|---|---|---|
| 217 | 2016-8-4 | 21 | 8 | 4 | 25 | 52 | 58 |
| 218 | 2016-8-5 | 12 | 5 | 3 | 18 | 34 | 51 |
| 219 | 2016-8-6 | 2 | 2 | 0 | 18 | 41 | 64 |
| 220 | 2016-8-7 | 6 | 2 | 2 | 17 | 38 | 44 |
| 221 | 2016-8-8 | 29 | 9 | 7 | 21 | 47 | 26 |
| 222 | 2016-8-9 | 21 | 7 | 8 | 14 | 28 | 19 |
| 223 | 2016-8-10 | 18 | 5 | 3 | 24 | 47 | 29 |
| 224 | 2016-8-11 | 16 | 5 | 2 | 16 | 31 | 20 |
| 225 | 2016-8-12 | 11 | 5 | 2 | 24 | 52 | 52 |
| 226 | 2016-8-13 | 7 | 5 | 1 | 28 | 59 | 103 |
| 227 | 2016-8-14 | 10 | 4 | 1 | 27 | 54 | 36 |
| 228 | 2016-8-15 | 18 | 4 | 5 | 19 | 38 | 28 |
| 229 | 2016-8-16 | 24 | 10 | 4 | 15 | 33 | 45 |
| 230 | 2016-8-17 | 19 | 13 | 4 | 21 | 44 | 77 |
| 231 | 2016-8-18 | 14 | 1 | 5 | 27 | 57 | 54 |
| 232 | 2016-8-19 | 16 | 4 | 4 | 23 | 46 | 24 |
| 233 | 2016-8-20 | 7 | 1 | 1 | 20 | 47 | 33 |
| 234 | 2016-8-21 | 9 | 3 | 5 | 16 | 36 | 29 |
| 235 | 2016-8-22 | 25 | 6 | 4 | 24 | 48 | 40 |
| 236 | 2016-8-23 | 18 | 6 | 8 | 22 | 39 | 40 |
| 237 | 2016-8-24 | 17 | 4 | 3 | 25 | 46 | 27 |
| 238 | 2016-8-25 | 12 | 4 | 5 | 25 | 50 | 45 |
| 239 | 2016-8-26 | 9 | 2 | 4 | 30 | 57 | 48 |
| 240 | 2016-8-27 | 7 | 2 | 2 | 22 | 43 | 35 |
| 241 | 2016-8-28 | 3 | 2 | 0 | 18 | 31 | 16 |
| 242 | 2016-8-29 | 23 | 8 | 4 | 21 | 47 | 48 |
| 243 | 2016-8-30 | 18 | 5 | 5 | 16 | 42 | 37 |
| 244 | 2016-8-31 | 17 | 4 | 2 | 20 | 42 | 36 |
| 245 | 2016-9-1 | 16 | 5 | 4 | 15 | 32 | 29 |
| 246 | 2016-9-2 | 14 | 7 | 1 | 26 | 51 | 71 |
| 247 | 2016-9-3 | 8 | 8 | 0 | 29 | 56 | 35 |
| 248 | 2016-9-4 | 7 | 3 | 4 | 16 | 35 | 26 |

| | | | | | | | |
|---|---|---|---|---|---|---|---|
| 249 | 2016-9-5 | 25 | 6 | 6 | 18 | 40 | 59 |
| 250 | 2016-9-6 | 22 | 4 | 5 | 21 | 46 | 63 |
| 251 | 2016-9-7 | 23 | 7 | 7 | 14 | 30 | 35 |
| 252 | 2016-9-8 | 20 | 8 | 3 | 27 | 56 | 60 |
| 253 | 2016-9-9 | 12 | 5 | 2 | 19 | 47 | 41 |
| 254 | 2016-9-10 | 6 | 5 | 0 | 15 | 31 | 20 |
| 255 | 2016-9-11 | 6 | 3 | 2 | 20 | 44 | 29 |
| 256 | 2016-9-12 | 17 | 1 | 7 | 30 | 63 | 34 |
| 257 | 2016-9-13 | 19 | 7 | 3 | 42 | 81 | 46 |
| 258 | 2016-9-14 | 14 | 3 | 3 | 34 | 72 | 49 |
| 259 | 2016-9-15 | 7 | 1 | 2 | 19 | 42 | 26 |
| 260 | 2016-9-16 | 7 | 5 | 1 | 18 | 34 | 28 |
| 261 | 2016-9-17 | 2 | 1 | 1 | 25 | 50 | 60 |
| 262 | 2016-9-18 | 37 | 11 | 7 | 23 | 44 | 32 |
| 263 | 2016-9-19 | 29 | 10 | 6 | 14 | 23 | 19 |
| 264 | 2016-9-20 | 19 | 4 | 4 | 9 | 16 | 15 |
| 265 | 2016-9-21 | 19 | 8 | 8 | 17 | 30 | 26 |
| 266 | 2016-9-22 | 14 | 6 | 3 | 36 | 71 | 37 |
| 267 | 2016-9-23 | 11 | 5 | 1 | 36 | 71 | 43 |
| 268 | 2016-9-24 | 13 | 5 | 1 | 31 | 64 | 28 |
| 269 | 2016-9-25 | 8 | 6 | 1 | 29 | 65 | 36 |
| 270 | 2016-9-26 | 13 | 6 | 1 | 25 | 59 | 30 |
| 271 | 2016-9-27 | 20 | 2 | 7 | 24 | 59 | 34 |
| 272 | 2016-9-28 | 10 | 4 | 4 | 18 | 46 | 27 |
| 273 | 2016-9-29 | 4 | 1 | 1 | 29 | 68 | 34 |
| 274 | 2016-9-30 | 12 | 5 | 3 | 32 | 66 | 40 |
| 275 | 2016-10-1 | 5 | 2 | 1 | 27 | 51 | 50 |
| 276 | 2016-10-2 | 12 | 2 | 6 | 28 | 52 | 38 |
| 277 | 2016-10-3 | 7 | 2 | 1 | 41 | 81 | 36 |
| 278 | 2016-10-4 | 7 | 2 | 0 | 39 | 81 | 41 |
| 279 | 2016-10-5 | 8 | 5 | 0 | 47 | 98 | 41 |
| 280 | 2016-10-6 | 15 | 8 | 3 | 47 | 92 | 63 |

| | | | | | | | |
|---|---|---|---|---|---|---|---|
| 281 | 2016-10-7 | 21 | 8 | 6 | 28 | 67 | 42 |
| 282 | 2016-10-8 | 24 | 8 | 6 | 22 | 52 | 53 |
| 283 | 2016-10-9 | 22 | 6 | 9 | 31 | 63 | 39 |
| 284 | 2016-10-10 | 29 | 10 | 8 | 26 | 61 | 30 |
| 285 | 2016-10-11 | 20 | 4 | 2 | 19 | 54 | 28 |
| 286 | 2016-10-12 | 24 | 10 | 6 | 25 | 63 | 32 |
| 287 | 2016-10-13 | 21 | 7 | 6 | 27 | 52 | 54 |
| 288 | 2016-10-14 | 12 | 3 | 2 | 22 | 45 | 26 |
| 289 | 2016-10-15 | 10 | 3 | 2 | 17 | 40 | 23 |
| 290 | 2016-10-16 | 4 | 1 | 0 | 22 | 53 | 48 |
| 291 | 2016-10-17 | 24 | 8 | 5 | 18 | 41 | 47 |
| 292 | 2016-10-18 | 26 | 6 | 12 | 24 | 52 | 64 |
| 293 | 2016-10-19 | 22 | 9 | 4 | 36 | 68 | 43 |
| 294 | 2016-10-20 | 16 | 8 | 3 | 37 | 72 | 47 |
| 295 | 2016-10-21 | 13 | 3 | 6 | 34 | 70 | 41 |
| 296 | 2016-10-22 | 7 | 2 | 1 | 35 | 73 | 45 |
| 297 | 2016-10-23 | 6 | 2 | 2 | 30 | 67 | 47 |
| 298 | 2016-10-24 | 21 | 6 | 4 | 33 | 73 | 47 |
| 299 | 2016-10-25 | 21 | 3 | 7 | 32 | 72 | 46 |
| 300 | 2016-10-26 | 24 | 10 | 3 | 22 | 53 | 30 |
| 301 | 2016-10-27 | 13 | 4 | 2 | 17 | 44 | 28 |
| 302 | 2016-10-28 | 11 | 5 | 2 | 21 | 59 | 37 |
| 303 | 2016-10-29 | 6 | 4 | 2 | 36 | 58 | 56 |
| 304 | 2016-10-30 | 12 | 4 | 2 | 25 | 47 | 59 |
| 305 | 2016-10-31 | 23 | 10 | 5 | 24 | 50 | 39 |
| 306 | 2016-11-1 | 19 | 8 | 2 | 23 | 50 | 29 |
| 307 | 2016-11-2 | 21 | 11 | 4 | 17 | 42 | 39 |
| 308 | 2016-11-3 | 17 | 6 | 2 | 18 | 47 | 41 |
| 309 | 2016-11-4 | 14 | 7 | 1 | 22 | 56 | 33 |
| 310 | 2016-11-5 | 11 | 5 | 3 | 26 | 66 | 36 |
| 311 | 2016-11-6 | 15 | 2 | 7 | 32 | 79 | 39 |
| 312 | 2016-11-7 | 35 | 9 | 7 | 31 | 73 | 55 |

| | | | | | | | |
|---|---|---|---|---|---|---|---|
| 313 | 2016-11-8 | 14 | 1 | 6 | 11 | 37 | 29 |
| 314 | 2016-11-9 | 20 | 7 | 4 | 16 | 31 | 51 |
| 315 | 2016-11-10 | 13 | 6 | 2 | 29 | 47 | 42 |
| 316 | 2016-11-11 | 19 | 6 | 5 | 35 | 60 | 54 |
| 317 | 2016-11-12 | 16 | 11 | 0 | 34 | 69 | 39 |
| 318 | 2016-11-13 | 8 | 2 | 3 | 25 | 59 | 30 |
| 319 | 2016-11-14 | 35 | 13 | 6 | 29 | 69 | 40 |
| 320 | 2016-11-15 | 26 | 9 | 3 | 28 | 62 | 36 |
| 321 | 2016-11-16 | 18 | 9 | 7 | 35 | 75 | 50 |
| 322 | 2016-11-17 | 17 | 4 | 3 | 37 | 79 | 62 |
| 323 | 2016-11-18 | 21 | 7 | 8 | 40 | 89 | 76 |
| 324 | 2016-11-19 | 15 | 6 | 5 | 35 | 77 | 45 |
| 325 | 2016-11-20 | 10 | 6 | 1 | 34 | 75 | 44 |
| 326 | 2016-11-21 | 13 | 2 | 4 | 34 | 77 | 39 |
| 327 | 2016-11-22 | 22 | 8 | 3 | 39 | 80 | 35 |
| 328 | 2016-11-23 | 20 | 6 | 3 | 36 | 75 | 34 |
| 329 | 2016-11-24 | 21 | 8 | 4 | 36 | 79 | 40 |
| 330 | 2016-11-25 | 20 | 8 | 1 | 35 | 84 | 41 |
| 331 | 2016-11-26 | 10 | 7 | 1 | 25 | 59 | 35 |
| 332 | 2016-11-27 | 9 | 3 | 2 | 35 | 77 | 84 |
| 333 | 2016-11-28 | 26 | 9 | 2 | 43 | 82 | 42 |
| 334 | 2016-11-29 | 20 | 9 | 4 | 30 | 52 | 37 |
| 335 | 2016-11-30 | 16 | 8 | 3 | 18 | 31 | 37 |
| 336 | 2016-12-1 | 24 | 11 | 4 | 29 | 49 | 54 |
| 337 | 2016-12-2 | 13 | 6 | 1 | 32 | 56 | 46 |
| 338 | 2016-12-3 | 7 | 4 | 2 | 32 | 64 | 50 |
| 339 | 2016-12-4 | 9 | 4 | 1 | 30 | 59 | 29 |
| 340 | 2016-12-5 | 28 | 4 | 6 | 37 | 70 | 26 |
| 341 | 2016-12-6 | 12 | 3 | 3 | 41 | 70 | 21 |
| 342 | 2016-12-7 | 22 | 10 | 2 | 41 | 73 | 26 |
| 343 | 2016-12-8 | 14 | 6 | 2 | 43 | 84 | 31 |
| 344 | 2016-12-9 | 22 | 7 | 7 | 43 | 82 | 24 |

| | | | | | | | |
|---|---|---|---|---|---|---|---|
| 345 | 2016-12-10 | 8 | 3 | 0 | 46 | 82 | 37 |
| 346 | 2016-12-11 | 12 | 4 | 1 | 39 | 72 | 27 |
| 347 | 2016-12-12 | 32 | 11 | 6 | 28 | 63 | 29 |
| 348 | 2016-12-13 | 26 | 9 | 8 | 27 | 63 | 31 |
| 349 | 2016-12-14 | 13 | 4 | 2 | 32 | 69 | 31 |
| 350 | 2016-12-15 | 14 | 8 | 0 | 27 | 53 | 36 |
| 351 | 2016-12-16 | 16 | 3 | 5 | 21 | 47 | 23 |
| 352 | 2016-12-17 | 8 | 4 | 0 | 33 | 67 | 30 |
| 353 | 2016-12-18 | 8 | 1 | 3 | 31 | 73 | 34 |
| 354 | 2016-12-19 | 25 | 9 | 4 | 25 | 66 | 36 |
| 355 | 2016-12-20 | 17 | 5 | 3 | 30 | 70 | 43 |
| 356 | 2016-12-21 | 17 | 4 | 3 | 30 | 66 | 33 |
| 357 | 2016-12-22 | 20 | 4 | 5 | 34 | 73 | 31 |
| 358 | 2016-12-23 | 19 | 5 | 6 | 32 | 70 | 27 |
| 359 | 2016-12-24 | 12 | 4 | 1 | 31 | 79 | 39 |
| 360 | 2016-12-25 | 6 | 2 | 2 | 60 | 81 | 62 |
| 361 | 2016-12-26 | 21 | 4 | 4 | 37 | 68 | 35 |
| 362 | 2016-12-27 | 11 | 2 | 3 | 18 | 47 | 22 |
| 363 | 2016-12-28 | 32 | 11 | 5 | 34 | 50 | 42 |
| 364 | 2016-12-29 | 10 | 2 | 4 | 34 | 65 | 59 |
| 365 | 2016-12-30 | 9 | 4 | 1 | 36 | 70 | 38 |
| 366 | 2016-12-31 | 8 | 4 | 3 | 42 | 84 | 41 |
| 367 | 2017-1-1 | 6 | 5 | 1 | 35 | 73 | 53 |
| 368 | 2017-1-2 | 18 | 6 | 7 | 29 | 66 | 37 |
| 369 | 2017-1-3 | 22 | 6 | 6 | 41 | 88 | 68 |
| 370 | 2017-1-4 | 30 | 9 | 5 | 17 | 36 | 35 |
| 371 | 2017-1-5 | 22 | 5 | 8 | 23 | 40 | 32 |
| 372 | 2017-1-6 | 13 | 3 | 4 | 29 | 55 | 49 |
| 373 | 2017-1-7 | 8 | 3 | 3 | 24 | 52 | 50 |
| 374 | 2017-1-8 | 6 | 3 | 0 | 35 | 74 | 64 |
| 375 | 2017-1-9 | 21 | 8 | 5 | 35 | 71 | 62 |
| 376 | 2017-1-10 | 17 | 6 | 3 | 31 | 65 | 85 |

| | | | | | | | |
|---|---|---|---|---|---|---|---|
| 377 | 2017-1-11 | 19 | 8 | 4 | 38 | 72 | 50 |
| 378 | 2017-1-12 | 12 | 4 | 5 | 38 | 77 | 52 |
| 379 | 2017-1-13 | 20 | 2 | 5 | 43 | 83 | 42 |
| 380 | 2017-1-14 | 14 | 6 | 2 | 39 | 77 | 77 |
| 381 | 2017-1-15 | 6 | 2 | 3 | 42 | 87 | 63 |
| 382 | 2017-1-16 | 23 | 5 | 3 | 50 | 95 | 61 |
| 383 | 2017-1-17 | 24 | 6 | 7 | 48 | 86 | 65 |
| 384 | 2017-1-18 | 9 | 2 | 0 | 48 | 82 | 85 |
| 385 | 2017-1-19 | 12 | 4 | 1 | 48 | 66 | 59 |
| 386 | 2017-1-20 | 9 | 3 | 1 | 43 | 80 | 59 |
| 387 | 2017-1-21 | 12 | 2 | 5 | 40 | 71 | 31 |
| 388 | 2017-1-22 | 6 | 1 | 1 | 60 | 82 | 35 |
| 389 | 2017-1-23 | 17 | 5 | 3 | 54 | 82 | 35 |
| 390 | 2017-1-24 | 12 | 5 | 2 | 43 | 73 | 56 |
| 391 | 2017-1-25 | 10 | 2 | 3 | 44 | 71 | 42 |
| 392 | 2017-1-26 | 7 | 4 | 1 | 46 | 69 | 34 |
| 393 | 2017-1-27 | 7 | 2 | 2 | 42 | 70 | 30 |
| 394 | 2017-1-28 | 5 | 4 | 0 | 54 | 95 | 48 |
| 395 | 2017-1-29 | 11 | 6 | 2 | 21 | 37 | 19 |
| 396 | 2017-1-30 | 11 | 4 | 1 | 32 | 53 | 38 |
| 397 | 2017-1-31 | 13 | 5 | 3 | 31 | 56 | 33 |
| 398 | 2017-2-1 | 12 | 4 | 2 | 35 | 61 | 53 |
| 399 | 2017-2-2 | 24 | 2 | 7 | 36 | 76 | 65 |
| 400 | 2017-2-3 | 14 | 2 | 3 | 21 | 58 | 38 |
| 401 | 2017-2-4 | 28 | 13 | 5 | 29 | 68 | 47 |
| 402 | 2017-2-5 | 11 | 4 | 3 | 41 | 80 | 51 |
| 403 | 2017-2-6 | 31 | 5 | 7 | 49 | 95 | 53 |
| 404 | 2017-2-7 | 27 | 7 | 10 | 52 | 105 | 78 |
| 405 | 2017-2-8 | 17 | 6 | 5 | 26 | 61 | 31 |
| 406 | 2017-2-9 | 17 | 1 | 7 | 38 | 80 | 39 |
| 407 | 2017-2-10 | 10 | 3 | 0 | 40 | 83 | 35 |
| 408 | 2017-2-11 | 8 | 2 | 1 | 31 | 76 | 35 |

| | | | | | | | |
|---|---|---|---|---|---|---|---|
| 409 | 2017-2-12 | 13 | 3 | 5 | 39 | 74 | 47 |
| 410 | 2017-2-13 | 32 | 9 | 7 | 37 | 82 | 35 |
| 411 | 2017-2-14 | 28 | 11 | 8 | 33 | 75 | 29 |
| 412 | 2017-2-15 | 16 | 8 | 2 | 37 | 92 | 50 |
| 413 | 2017-2-16 | 19 | 8 | 5 | 28 | 62 | 46 |
| 414 | 2017-2-17 | 10 | 2 | 4 | 38 | 85 | 58 |
| 415 | 2017-2-18 | 10 | 3 | 4 | 33 | 71 | 48 |
| 416 | 2017-2-19 | 7 | 0 | 2 | 31 | 68 | 39 |
| 417 | 2017-2-20 | 29 | 11 | 5 | 29 | 68 | 30 |
| 418 | 2017-2-21 | 29 | 13 | 9 | 26 | 48 | 21 |
| 419 | 2017-2-22 | 15 | 3 | 3 | 45 | 75 | 41 |
| 420 | 2017-2-23 | 16 | 3 | 4 | 48 | 79 | 38 |
| 421 | 2017-2-24 | 16 | 4 | 5 | 39 | 68 | 47 |
| 422 | 2017-2-25 | 11 | 3 | 3 | 27 | 61 | 32 |
| 423 | 2017-2-26 | 10 | 4 | 2 | 32 | 64 | 29 |
| 424 | 2017-2-27 | 23 | 5 | 9 | 33 | 60 | 35 |
| 425 | 2017-2-28 | 21 | 11 | 4 | 35 | 64 | 46 |
| 426 | 2017-3-1 | 20 | 9 | 4 | 38 | 80 | 35 |
| 427 | 2017-3-2 | 23 | 10 | 6 | 40 | 68 | 40 |
| 428 | 2017-3-3 | 22 | 9 | 6 | 39 | 83 | 36 |
| 429 | 2017-3-4 | 8 | 3 | 2 | 29 | 56 | 29 |
| 430 | 2017-3-5 | 10 | 4 | 4 | 28 | 50 | 29 |
| 431 | 2017-3-6 | 32 | 10 | 9 | 27 | 48 | 21 |
| 432 | 2017-3-7 | 19 | 3 | 5 | 27 | 49 | 27 |
| 433 | 2017-3-8 | 15 | 4 | 4 | 21 | 41 | 27 |
| 434 | 2017-3-9 | 22 | 8 | 6 | 19 | 38 | 24 |
| 435 | 2017-3-10 | 27 | 8 | 9 | 22 | 44 | 28 |
| 436 | 2017-3-11 | 12 | 5 | 4 | 26 | 43 | 22 |
| 437 | 2017-3-12 | 6 | 4 | 0 | 15 | 27 | 16 |
| 438 | 2017-3-13 | 35 | 7 | 6 | 14 | 33 | 17 |
| 439 | 2017-3-14 | 30 | 10 | 9 | 17 | 33 | 17 |
| 440 | 2017-3-15 | 25 | 7 | 5 | 17 | 38 | 22 |

| | | | | | | | |
|---|---|---|---|---|---|---|---|
| 441 | 2017-3-16 | 18 | 9 | 2 | 22 | 44 | 24 |
| 442 | 2017-3-17 | 16 | 5 | 5 | 28 | 52 | 25 |
| 443 | 2017-3-18 | 12 | 8 | 0 | 37 | 65 | 37 |
| 444 | 2017-3-19 | 11 | 5 | 3 | 28 | 51 | 25 |
| 445 | 2017-3-20 | 36 | 9 | 12 | 29 | 57 | 22 |
| 446 | 2017-3-21 | 25 | 5 | 7 | 29 | 44 | 24 |
| 447 | 2017-3-22 | 13 | 4 | 2 | 30 | 62 | 33 |
| 448 | 2017-3-23 | 19 | 7 | 4 | 33 | 77 | 32 |
| 449 | 2017-3-24 | 20 | 9 | 6 | 32 | 57 | 23 |
| 450 | 2017-3-25 | 16 | 6 | 2 | 40 | 73 | 27 |
| 451 | 2017-3-26 | 7 | 3 | 0 | 30 | 48 | 30 |
| 452 | 2017-3-27 | 26 | 7 | 5 | 46 | 66 | 23 |
| 453 | 2017-3-28 | 15 | 5 | 3 | 34 | 59 | 33 |
| 454 | 2017-3-29 | 27 | 14 | 3 | 37 | 76 | 32 |
| 455 | 2017-3-30 | 13 | 1 | 4 | 43 | 71 | 24 |
| 456 | 2017-3-31 | 12 | 5 | 2 | 14 | 23 | 22 |
| 457 | 2017-4-1 | 7 | 4 | 1 | 29 | 52 | 41 |
| 458 | 2017-4-2 | 6 | 2 | 1 | 26 | 39 | 37 |
| 459 | 2017-4-3 | 10 | 4 | 1 | 21 | 36 | 19 |
| 460 | 2017-4-4 | 4 | 1 | 0 | 19 | 38 | 26 |
| 461 | 2017-4-5 | 34 | 13 | 7 | 15 | 29 | 19 |
| 462 | 2017-4-6 | 27 | 7 | 7 | 18 | 41 | 35 |
| 463 | 2017-4-7 | 14 | 5 | 3 | 27 | 59 | 24 |
| 464 | 2017-4-8 | 7 | 2 | 1 | 21 | 44 | 28 |
| 465 | 2017-4-9 | 4 | 3 | 0 | 20 | 41 | 27 |
| 466 | 2017-4-10 | 42 | 13 | 7 | 20 | 47 | 31 |
| 467 | 2017-4-11 | 22 | 9 | 4 | 28 | 53 | 23 |
| 468 | 2017-4-12 | 12 | 6 | 4 | 29 | 66 | 24 |
| 469 | 2017-4-13 | 16 | 8 | 1 | 43 | 82 | 26 |
| 470 | 2017-4-14 | 27 | 8 | 7 | 69 | 120 | 43 |
| 471 | 2017-4-15 | 5 | 2 | 0 | 63 | 102 | 21 |
| 472 | 2017-4-16 | 6 | 4 | 0 | 64 | 109 | 23 |

| | | | | | | | |
|---|---|---|---|---|---|---|---|
| 473 | 2017-4-17 | 31 | 7 | 7 | 16 | 24 | 21 |
| 474 | 2017-4-18 | 25 | 7 | 6 | 33 | 53 | 25 |
| 475 | 2017-4-19 | 12 | 3 | 6 | 40 | 72 | 33 |
| 476 | 2017-4-20 | 24 | 6 | 5 | 34 | 68 | 36 |
| 477 | 2017-4-21 | 20 | 6 | 4 | 16 | 39 | 24 |
| 478 | 2017-4-22 | 12 | 4 | 2 | 22 | 37 | 34 |
| 479 | 2017-4-23 | 8 | 4 | 1 | 34 | 59 | 32 |
| 480 | 2017-4-24 | 29 | 8 | 7 | 24 | 40 | 37 |
| 481 | 2017-4-25 | 23 | 7 | 6 | 23 | 47 | 32 |
| 482 | 2017-4-26 | 17 | 6 | 4 | 13 | 26 | 23 |
| 483 | 2017-4-27 | 16 | 7 | 3 | 17 | 37 | 25 |
| 484 | 2017-4-28 | 14 | 4 | 2 | 21 | 43 | 30 |
| 485 | 2017-4-29 | 4 | 2 | 0 | 34 | 64 | 35 |
| 486 | 2017-4-30 | 7 | 3 | 2 | 37 | 77 | 37 |
| 487 | 2017-5-1 | 8 | 4 | 0 | 27 | 51 | 29 |
| 488 | 2017-5-2 | 27 | 6 | 10 | 23 | 52 | 32 |
| 489 | 2017-5-3 | 21 | 8 | 6 | 22 | 46 | 33 |
| 490 | 2017-5-4 | 25 | 8 | 4 | 23 | 51 | 19 |
| 491 | 2017-5-5 | 12 | 6 | 0 | 31 | 62 | 23 |
| 492 | 2017-5-6 | 7 | 3 | 3 | 29 | 59 | 25 |
| 493 | 2017-5-7 | 8 | 4 | 2 | 31 | 61 | 23 |
| 494 | 2017-5-8 | 24 | 7 | 6 | 28 | 57 | 33 |
| 495 | 2017-5-9 | 21 | 5 | 4 | 26 | 55 | 29 |
| 496 | 2017-5-10 | 14 | 7 | 6 | 28 | 55 | 23 |
| 497 | 2017-5-11 | 21 | 8 | 6 | 18 | 34 | 20 |
| 498 | 2017-5-12 | 8 | 2 | 4 | 18 | 36 | 19 |
| 499 | 2017-5-13 | 4 | 3 | 0 | 31 | 68 | 45 |
| 500 | 2017-5-14 | 7 | 3 | 1 | 30 | 62 | 32 |
| 501 | 2017-5-15 | 28 | 7 | 8 | 17 | 33 | 19 |
| 502 | 2017-5-16 | 21 | 11 | 1 | 21 | 35 | 32 |
| 503 | 2017-5-17 | 18 | 10 | 4 | 20 | 33 | 21 |
| 504 | 2017-5-18 | 17 | 1 | 9 | 26 | 55 | 52 |

| | | | | | | | |
|---|---|---|---|---|---|---|---|
| 505 | 2017-5-19 | 18 | 6 | 7 | 24 | 50 | 39 |
| 506 | 2017-5-20 | 9 | 5 | 3 | 26 | 66 | 24 |
| 507 | 2017-5-21 | 11 | 5 | 2 | 28 | 66 | 23 |
| 508 | 2017-5-22 | 22 | 8 | 6 | 26 | 64 | 19 |
| 509 | 2017-5-23 | 21 | 5 | 7 | 23 | 57 | 26 |
| 510 | 2017-5-24 | 21 | 9 | 3 | 16 | 41 | 20 |
| 511 | 2017-5-25 | 14 | 7 | 2 | 16 | 34 | 19 |
| 512 | 2017-5-26 | 13 | 8 | 1 | 17 | 33 | 33 |
| 513 | 2017-5-27 | 5 | 2 | 2 | 32 | 75 | 31 |
| 514 | 2017-5-28 | 8 | 3 | 2 | 39 | 66 | 27 |
| 515 | 2017-5-29 | 5 | 3 | 1 | 37 | 60 | 21 |
| 516 | 2017-5-30 | 14 | 6 | 5 | 32 | 63 | 32 |
| 517 | 2017-5-31 | 29 | 11 | 3 | 22 | 43 | 20 |
| 518 | 2017-6-1 | 17 | 4 | 5 | 18 | 36 | 12 |
| 519 | 2017-6-2 | 5 | 1 | 0 | 19 | 38 | 20 |
| 520 | 2017-6-3 | 6 | 3 | 2 | 15 | 33 | 14 |
| 521 | 2017-6-4 | 3 | 3 | 0 | 14 | 28 | 15 |
| 522 | 2017-6-5 | 27 | 9 | 5 | 19 | 40 | 17 |
| 523 | 2017-6-6 | 15 | 7 | 1 | 11 | 30 | 14 |
| 524 | 2017-6-7 | 24 | 10 | 6 | 25 | 43 | 24 |
| 525 | 2017-6-8 | 14 | 4 | 3 | 22 | 42 | 15 |
| 526 | 2017-6-9 | 22 | 11 | 3 | 14 | 25 | 12 |
| 527 | 2017-6-10 | 6 | 6 | 0 | 15 | 30 | 14 |
| 528 | 2017-6-11 | 6 | 1 | 0 | 13 | 32 | 23 |
| 529 | 2017-6-12 | 17 | 5 | 4 | 9 | 26 | 18 |
| 530 | 2017-6-13 | 12 | 4 | 1 | 15 | 33 | 27 |
| 531 | 2017-6-14 | 16 | 7 | 2 | 13 | 31 | 22 |
| 532 | 2017-6-15 | 9 | 4 | 1 | 14 | 33 | 22 |
| 533 | 2017-6-16 | 13 | 5 | 3 | 12 | 26 | 17 |
| 534 | 2017-6-17 | 10 | 5 | 1 | 21 | 37 | 32 |
| 535 | 2017-6-18 | 12 | 3 | 4 | 16 | 33 | 24 |
| 536 | 2017-6-19 | 20 | 7 | 4 | 19 | 34 | 25 |

| | | | | | | | |
|---|---|---|---|---|---|---|---|
| 537 | 2017-6-20 | 18 | 7 | 2 | 26 | 48 | 40 |
| 538 | 2017-6-21 | 19 | 10 | 3 | 31 | 56 | 59 |
| 539 | 2017-6-22 | 18 | 6 | 7 | 14 | 36 | 24 |
| 540 | 2017-6-23 | 12 | 4 | 3 | 15 | 36 | 25 |
| 541 | 2017-6-24 | 4 | 2 | 0 | 19 | 46 | 26 |
| 542 | 2017-6-25 | 4 | 4 | 0 | 13 | 31 | 22 |
| 543 | 2017-6-26 | 24 | 9 | 7 | 18 | 33 | 19 |
| 544 | 2017-6-27 | 22 | 5 | 2 | 29 | 43 | 28 |
| 545 | 2017-6-28 | 24 | 8 | 4 | 21 | 47 | 43 |
| 546 | 2017-6-29 | 21 | 6 | 5 | 18 | 30 | 15 |
| 547 | 2017-6-30 | 19 | 6 | 5 | 19 | 35 | 31 |
| 548 | 2017-7-1 | 4 | 1 | 1 | 24 | 44 | 39 |
| 549 | 2017-7-2 | 10 | 4 | 4 | 22 | 39 | 31 |
| 550 | 2017-7-3 | 32 | 9 | 6 | 17 | 30 | 27 |
| 551 | 2017-7-4 | 16 | 5 | 3 | 29 | 54 | 24 |
| 552 | 2017-7-5 | 13 | 5 | 3 | 30 | 66 | 31 |
| 553 | 2017-7-6 | 15 | 5 | 4 | 20 | 44 | 26 |
| 554 | 2017-7-7 | 15 | 6 | 2 | 10 | 22 | 18 |
| 555 | 2017-7-8 | 10 | 3 | 5 | 17 | 35 | 36 |
| 556 | 2017-7-9 | 4 | 3 | 0 | 19 | 42 | 19 |
| 557 | 2017-7-10 | 20 | 3 | 7 | 30 | 60 | 28 |
| 558 | 2017-7-11 | 17 | 6 | 7 | 50 | 102 | 36 |
| 559 | 2017-7-12 | 27 | 8 | 10 | 40 | 83 | 27 |
| 560 | 2017-7-13 | 17 | 7 | 3 | 29 | 48 | 24 |
| 561 | 2017-7-14 | 16 | 3 | 7 | 15 | 33 | 37 |
| 562 | 2017-7-15 | 9 | 4 | 2 | 13 | 27 | 31 |
| 563 | 2017-7-16 | 11 | 5 | 3 | 17 | 38 | 39 |
| 564 | 2017-7-17 | 33 | 11 | 7 | 23 | 43 | 39 |
| 565 | 2017-7-18 | 23 | 12 | 6 | 17 | 34 | 50 |
| 566 | 2017-7-19 | 21 | 7 | 4 | 17 | 34 | 29 |
| 567 | 2017-7-20 | 16 | 8 | 3 | 22 | 41 | 28 |
| 568 | 2017-7-21 | 17 | 4 | 6 | 18 | 29 | 24 |

| | | | | | | | |
|---|---|---|---|---|---|---|---|
| 569 | 2017-7-22 | 4 | 1 | 2 | 14 | 24 | 29 |
| 570 | 2017-7-23 | 14 | 7 | 3 | 15 | 29 | 39 |
| 571 | 2017-7-24 | 28 | 13 | 7 | 21 | 37 | 28 |
| 572 | 2017-7-25 | 33 | 14 | 8 | 25 | 45 | 20 |
| 573 | 2017-7-26 | 16 | 9 | 3 | 20 | 33 | 20 |
| 574 | 2017-7-27 | 10 | 3 | 1 | 21 | 40 | 21 |
| 575 | 2017-7-28 | 15 | 7 | 8 | 16 | 34 | 12 |
| 576 | 2017-7-29 | 7 | 0 | 3 | 24 | 44 | 26 |
| 577 | 2017-7-30 | 8 | 3 | 1 | 31 | 53 | 23 |
| 578 | 2017-7-31 | 28 | 11 | 8 | 12 | 23 | 19 |
| 579 | 2017-8-1 | 21 | 12 | 3 | 16 | 34 | 22 |
| 580 | 2017-8-2 | 14 | 7 | 3 | 23 | 39 | 25 |
| 581 | 2017-8-3 | 12 | 4 | 5 | 15 | 30 | 19 |
| 582 | 2017-8-4 | 15 | 7 | 2 | 17 | 35 | 26 |
| 583 | 2017-8-5 | 7 | 3 | 2 | 22 | 40 | 23 |
| 584 | 2017-8-6 | 11 | 5 | 2 | 18 | 32 | 26 |
| 585 | 2017-8-7 | 20 | 6 | 7 | 17 | 33 | 13 |
| 586 | 2017-8-8 | 21 | 8 | 0 | 10 | 23 | 14 |
| 587 | 2017-8-9 | 17 | 8 | 4 | 12 | 28 | 24 |
| 588 | 2017-8-10 | 16 | 7 | 3 | 19 | 38 | 14 |
| 589 | 2017-8-11 | 7 | 3 | 1 | 17 | 35 | 17 |
| 590 | 2017-8-12 | 9 | 3 | 2 | 23 | 45 | 13 |
| 591 | 2017-8-13 | 6 | 3 | 2 | 18 | 39 | 12 |
| 592 | 2017-8-14 | 36 | 8 | 8 | 9 | 19 | 13 |
| 593 | 2017-8-15 | 19 | 9 | 4 | 13 | 25 | 14 |
| 594 | 2017-8-16 | 18 | 7 | 4 | 22 | 39 | 20 |
| 595 | 2017-8-17 | 17 | 6 | 2 | 25 | 48 | 15 |
| 596 | 2017-8-18 | 8 | 2 | 0 | 11 | 28 | 11 |
| 597 | 2017-8-19 | 7 | 4 | 0 | 13 | 31 | 14 |
| 598 | 2017-8-20 | 5 | 2 | 1 | 13 | 29 | 14 |
| 599 | 2017-8-21 | 32 | 12 | 8 | 14 | 32 | 14 |
| 600 | 2017-8-22 | 19 | 7 | 7 | 16 | 34 | 15 |

| | | | | | | | |
|---|---|---|---|---|---|---|---|
| 601 | 2017-8-23 | 13 | 6 | 4 | 21 | 42 | 20 |
| 602 | 2017-8-24 | 11 | 1 | 3 | 21 | 40 | 29 |
| 603 | 2017-8-25 | 12 | 4 | 4 | 16 | 29 | 12 |
| 604 | 2017-8-26 | 11 | 6 | 2 | 14 | 31 | 10 |
| 605 | 2017-8-27 | 7 | 1 | 3 | 17 | 33 | 13 |
| 606 | 2017-8-28 | 21 | 7 | 5 | 20 | 38 | 15 |
| 607 | 2017-8-29 | 22 | 9 | 2 | 18 | 36 | 14 |
| 608 | 2017-8-30 | 9 | 2 | 3 | 13 | 27 | 13 |
| 609 | 2017-8-31 | 16 | 2 | 5 | 16 | 32 | 15 |
| 610 | 2017-9-1 | 13 | 4 | 3 | 16 | 33 | 14 |
| 611 | 2017-9-2 | 6 | 2 | 1 | 11 | 24 | 14 |
| 612 | 2017-9-3 | 4 | 2 | 1 | 12 | 25 | 18 |
| 613 | 2017-9-4 | 24 | 7 | 7 | 17 | 30 | 18 |
| 614 | 2017-9-5 | 22 | 5 | 4 | 14 | 29 | 14 |
| 615 | 2017-9-6 | 25 | 7 | 6 | 8 | 17 | 10 |
| 616 | 2017-9-7 | 17 | 6 | 2 | 14 | 32 | 14 |
| 617 | 2017-9-8 | 9 | 4 | 2 | 16 | 35 | 17 |
| 618 | 2017-9-9 | 11 | 5 | 2 | 14 | 27 | 17 |
| 619 | 2017-9-10 | 6 | 3 | 2 | 8 | 17 | 14 |
| 620 | 2017-9-11 | 39 | 10 | 12 | 16 | 34 | 21 |
| 621 | 2017-9-12 | 24 | 7 | 9 | 28 | 50 | 25 |
| 622 | 2017-9-13 | 26 | 9 | 5 | 38 | 60 | 21 |
| 623 | 2017-9-14 | 13 | 4 | 4 | 25 | 40 | 26 |
| 624 | 2017-9-15 | 15 | 6 | 2 | 29 | 52 | 27 |
| 625 | 2017-9-16 | 5 | 3 | 2 | 28 | 44 | 23 |
| 626 | 2017-9-17 | 5 | 1 | 1 | 19 | 36 | 34 |
| 627 | 2017-9-18 | 27 | 9 | 6 | 17 | 32 | 20 |
| 628 | 2017-9-19 | 28 | 7 | 4 | 15 | 36 | 18 |
| 629 | 2017-9-20 | 16 | 6 | 7 | 15 | 47 | 22 |
| 630 | 2017-9-21 | 26 | 8 | 5 | 18 | 33 | 25 |
| 631 | 2017-9-22 | 13 | 2 | 4 | 19 | 32 | 35 |
| 632 | 2017-9-23 | 12 | 7 | 2 | 29 | 53 | 61 |

| | | | | | | | |
|---|---|---|---|---|---|---|---|
| 633 | 2017-9-24 | 12 | 7 | 4 | 17 | 39 | 26 |
| 634 | 2017-9-25 | 25 | 8 | 6 | 22 | 44 | 23 |
| 635 | 2017-9-26 | 16 | 6 | 2 | 26 | 50 | 44 |
| 636 | 2017-9-27 | 19 | 9 | 7 | 21 | 44 | 21 |
| 637 | 2017-9-28 | 9 | 2 | 5 | 11 | 30 | 26 |
| 638 | 2017-9-29 | 8 | 2 | 2 | 26 | 54 | 86 |
| 639 | 2017-9-30 | 6 | 5 | 0 | 23 | 52 | 34 |
| 640 | 2017-10-1 | 5 | 2 | 2 | 17 | 42 | 27 |
| 641 | 2017-10-2 | 11 | 3 | 2 | 22 | 46 | 37 |
| 642 | 2017-10-3 | 6 | 3 | 1 | 18 | 38 | 30 |
| 643 | 2017-10-4 | 6 | 2 | 2 | 17 | 36 | 27 |
| 644 | 2017-10-5 | 10 | 3 | 4 | 17 | 36 | 27 |
| 645 | 2017-10-6 | 5 | 4 | 1 | 19 | 42 | 28 |
| 646 | 2017-10-7 | 14 | 7 | 2 | 21 | 42 | 26 |
| 647 | 2017-10-8 | 18 | 3 | 3 | 20 | 39 | 17 |
| 648 | 2017-10-9 | 30 | 8 | 6 | 20 | 46 | 30 |
| 649 | 2017-10-10 | 23 | 7 | 4 | 22 | 45 | 36 |
| 650 | 2017-10-11 | 24 | 7 | 8 | 28 | 45 | 37 |
| 651 | 2017-10-12 | 22 | 9 | 6 | 22 | 38 | 26 |
| 652 | 2017-10-13 | 14 | 8 | 3 | 15 | 30 | 44 |
| 653 | 2017-10-14 | 10 | 4 | 1 | 21 | 43 | 44 |
| 654 | 2017-10-15 | 8 | 1 | 2 | 16 | 30 | 51 |
| 655 | 2017-10-16 | 33 | 15 | 5 | 20 | 35 | 28 |
| 656 | 2017-10-17 | 23 | 10 | 8 | 28 | 53 | 56 |
| 657 | 2017-10-18 | 13 | 8 | 2 | 27 | 48 | 51 |
| 658 | 2017-10-19 | 14 | 6 | 1 | 23 | 45 | 73 |
| 659 | 2017-10-20 | 12 | 1 | 2 | 23 | 47 | 51 |
| 660 | 2017-10-21 | 8 | 6 | 0 | 28 | 55 | 44 |
| 661 | 2017-10-22 | 5 | 1 | 0 | 15 | 28 | 50 |
| 662 | 2017-10-23 | 23 | 4 | 11 | 12 | 23 | 35 |
| 663 | 2017-10-24 | 23 | 8 | 4 | 21 | 44 | 73 |
| 664 | 2017-10-25 | 19 | 4 | 6 | 25 | 47 | 43 |

| | | | | | | | |
|---|---|---|---|---|---|---|---|
| 665 | 2017-10-26 | 19 | 7 | 4 | 28 | 54 | 34 |
| 666 | 2017-10-27 | 24 | 11 | 6 | 27 | 52 | 37 |
| 667 | 2017-10-28 | 9 | 4 | 2 | 34 | 61 | 41 |
| 668 | 2017-10-29 | 7 | 1 | 3 | 33 | 69 | 48 |
| 669 | 2017-10-30 | 24 | 9 | 6 | 46 | 78 | 51 |
| 670 | 2017-10-31 | 29 | 6 | 10 | 33 | 62 | 50 |
| 671 | 2017-11-1 | 29 | 11 | 5 | 35 | 63 | 40 |
| 672 | 2017-11-2 | 17 | 5 | 5 | 26 | 47 | 23 |
| 673 | 2017-11-3 | 15 | 6 | 3 | 16 | 33 | 25 |
| 674 | 2017-11-4 | 5 | 2 | 1 | 22 | 38 | 22 |
| 675 | 2017-11-5 | 6 | 3 | 2 | 38 | 67 | 46 |
| 676 | 2017-11-6 | 30 | 10 | 8 | 55 | 95 | 70 |
| 677 | 2017-11-7 | 16 | 4 | 2 | 57 | 93 | 48 |
| 678 | 2017-11-8 | 22 | 10 | 8 | 56 | 92 | 40 |
| 679 | 2017-11-9 | 20 | 5 | 4 | 48 | 80 | 38 |
| 680 | 2017-11-10 | 13 | 4 | 2 | 38 | 73 | 38 |
| 681 | 2017-11-11 | 4 | 1 | 1 | 33 | 61 | 39 |
| 682 | 2017-11-12 | 13 | 5 | 4 | 34 | 68 | 45 |
| 683 | 2017-11-13 | 36 | 9 | 10 | 37 | 72 | 51 |
| 684 | 2017-11-14 | 20 | 7 | 5 | 35 | 67 | 46 |
| 685 | 2017-11-15 | 22 | 8 | 6 | 34 | 67 | 39 |
| 686 | 2017-11-16 | 17 | 6 | 4 | 41 | 78 | 51 |
| 687 | 2017-11-17 | 19 | 3 | 5 | 47 | 97 | 72 |
| 688 | 2017-11-18 | 6 | 2 | 2 | 28 | 57 | 38 |
| 689 | 2017-11-19 | 6 | 0 | 2 | 31 | 66 | 40 |
| 690 | 2017-11-20 | 36 | 11 | 9 | 29 | 71 | 47 |
| 691 | 2017-11-21 | 23 | 7 | 6 | 30 | 70 | 44 |
| 692 | 2017-11-22 | 16 | 6 | 7 | 31 | 72 | 39 |
| 693 | 2017-11-23 | 16 | 6 | 4 | 23 | 53 | 30 |
| 694 | 2017-11-24 | 18 | 3 | 8 | 22 | 41 | 28 |
| 695 | 2017-11-25 | 4 | 1 | 1 | 36 | 65 | 38 |
| 696 | 2017-11-26 | 6 | 2 | 1 | 36 | 69 | 39 |

| | | | | | | | |
|---|---|---|---|---|---|---|---|
| 697 | 2017-11-27 | 25 | 8 | 6 | 32 | 65 | 45 |
| 698 | 2017-11-28 | 13 | 3 | 2 | 30 | 71 | 47 |
| 699 | 2017-11-29 | 25 | 7 | 7 | 32 | 72 | 63 |
| 700 | 2017-11-30 | 26 | 7 | 4 | 35 | 74 | 49 |
| 701 | 2017-12-1 | 13 | 1 | 4 | 31 | 67 | 42 |
| 702 | 2017-12-2 | 7 | 5 | 1 | 28 | 60 | 41 |
| 703 | 2017-12-3 | 4 | 1 | 2 | 30 | 60 | 41 |
| 704 | 2017-12-4 | 33 | 10 | 3 | 30 | 62 | 34 |
| 705 | 2017-12-5 | 17 | 4 | 4 | 33 | 63 | 42 |
| 706 | 2017-12-6 | 17 | 5 | 3 | 33 | 65 | 33 |
| 707 | 2017-12-7 | 22 | 6 | 6 | 34 | 67 | 33 |
| 708 | 2017-12-8 | 10 | 1 | 2 | 31 | 58 | 29 |
| 709 | 2017-12-9 | 6 | 1 | 3 | 46 | 76 | 36 |
| 710 | 2017-12-10 | 9 | 2 | 5 | 59 | 104 | 52 |
| 711 | 2017-12-11 | 35 | 11 | 7 | 70 | 119 | 49 |
| 712 | 2017-12-12 | 29 | 13 | 7 | 55 | 95 | 31 |
| 713 | 2017-12-13 | 20 | 9 | 4 | 38 | 80 | 32 |
| 714 | 2017-12-14 | 16 | 5 | 4 | 32 | 68 | 30 |
| 715 | 2017-12-15 | 19 | 6 | 4 | 37 | 81 | 31 |
| 716 | 2017-12-16 | 11 | 3 | 6 | 25 | 56 | 32 |
| 717 | 2017-12-17 | 7 | 1 | 4 | 30 | 64 | 48 |
| 718 | 2017-12-18 | 23 | 3 | 7 | 31 | 68 | 52 |
| 719 | 2017-12-19 | 16 | 4 | 6 | 22 | 47 | 25 |
| 720 | 2017-12-20 | 8 | 3 | 2 | 30 | 70 | 26 |
| 721 | 2017-12-21 | 18 | 5 | 7 | 39 | 78 | 35 |
| 722 | 2017-12-22 | 8 | 4 | 1 | 48 | 93 | 50 |
| 723 | 2017-12-23 | 11 | 2 | 2 | 53 | 99 | 47 |
| 724 | 2017-12-24 | 12 | 5 | 3 | 58 | 101 | 27 |
| 725 | 2017-12-25 | 18 | 4 | 3 | 66 | 119 | 39 |
| 726 | 2017-12-26 | 22 | 6 | 8 | 65 | 124 | 41 |
| 727 | 2017-12-27 | 11 | 5 | 1 | 58 | 123 | 51 |
| 728 | 2017-12-28 | 24 | 12 | 2 | 39 | 85 | 26 |

| | | | | | | | |
|---|---|---|---|---|---|---|---|
| 729 | 2017-12-29 | 18 | 8 | 4 | 34 | 73 | 26 |
| 730 | 2017-12-30 | 10 | 2 | 5 | 31 | 74 | 23 |
| 731 | 2017-12-31 | 8 | 5 | 2 | 35 | 84 | 49 |
| 732 | 2018-1-1 | 13 | 5 | 5 | 46 | 105 | 66 |
| 733 | 2018-1-2 | 27 | 7 | 8 | 57 | 137 | 137 |
| 734 | 2018-1-3 | 31 | 9 | 8 | 12 | 30 | 35 |
| 735 | 2018-1-4 | 15 | 7 | 5 | 16 | 35 | 32 |
| 736 | 2018-1-5 | 20 | 7 | 4 | 27 | 54 | 49 |
| 737 | 2018-1-6 | 10 | 4 | 1 | 33 | 72 | 108 |
| 738 | 2018-1-7 | 9 | 3 | 2 | 21 | 51 | 63 |
| 739 | 2018-1-8 | 30 | 11 | 6 | 25 | 54 | 55 |
| 740 | 2018-1-9 | 30 | 10 | 8 | 30 | 68 | 42 |
| 741 | 2018-1-10 | 20 | 12 | 6 | 36 | 62 | 34 |
| 742 | 2018-1-11 | 23 | 4 | 7 | 42 | 69 | 45 |
| 743 | 2018-1-12 | 17 | 6 | 5 | 45 | 73 | 48 |
| 744 | 2018-1-13 | 8 | 2 | 2 | 29 | 50 | 38 |
| 745 | 2018-1-14 | 4 | 1 | 1 | 40 | 63 | 45 |
| 746 | 2018-1-15 | 22 | 3 | 6 | 44 | 78 | 38 |
| 747 | 2018-1-16 | 13 | 4 | 2 | 40 | 75 | 40 |
| 748 | 2018-1-17 | 16 | 6 | 3 | 48 | 86 | 47 |
| 749 | 2018-1-18 | 15 | 3 | 5 | 55 | 95 | 46 |
| 750 | 2018-1-19 | 20 | 7 | 2 | 47 | 88 | 46 |
| 751 | 2018-1-20 | 11 | 7 | 3 | 51 | 92 | 44 |
| 752 | 2018-1-21 | 15 | 7 | 3 | 46 | 82 | 41 |
| 753 | 2018-1-22 | 37 | 14 | 8 | 58 | 103 | 51 |
| 754 | 2018-1-23 | 23 | 9 | 4 | 57 | 98 | 53 |
| 755 | 2018-1-24 | 14 | 6 | 5 | 49 | 94 | 58 |
| 756 | 2018-1-25 | 13 | 4 | 4 | 42 | 86 | 59 |
| 757 | 2018-1-26 | 13 | 4 | 3 | 39 | 70 | 36 |
| 758 | 2018-1-27 | 7 | 3 | 2 | 29 | 56 | 23 |
| 759 | 2018-1-28 | 7 | 1 | 1 | 34 | 66 | 42 |
| 760 | 2018-1-29 | 35 | 9 | 8 | 55 | 103 | 51 |

| | | | | | | | |
|---|---|---|---|---|---|---|---|
| 761 | 2018-1-30 | 24 | 6 | 4 | 54 | 98 | 54 |
| 762 | 2018-1-31 | 17 | 8 | 5 | 38 | 73 | 34 |
| 763 | 2018-2-1 | 12 | 3 | 3 | 48 | 79 | 48 |
| 764 | 2018-2-2 | 12 | 4 | 4 | 25 | 46 | 22 |
| 765 | 2018-2-3 | 10 | 5 | 1 | 35 | 63 | 54 |
| 766 | 2018-2-4 | 7 | 1 | 1 | 37 | 61 | 33 |
| 767 | 2018-2-5 | 22 | 7 | 4 | 33 | 51 | 29 |
| 768 | 2018-2-6 | 11 | 2 | 2 | 49 | 79 | 50 |
| 769 | 2018-2-7 | 18 | 6 | 5 | 50 | 75 | 26 |
| 770 | 2018-2-8 | 14 | 3 | 2 | 37 | 63 | 34 |
| 771 | 2018-2-9 | 14 | 5 | 5 | 30 | 62 | 38 |
| 772 | 2018-2-10 | 7 | 4 | 1 | 29 | 48 | 21 |
| 773 | 2018-2-11 | 12 | 3 | 3 | 33 | 52 | 29 |
| 774 | 2018-2-12 | 14 | 3 | 7 | 41 | 70 | 36 |
| 775 | 2018-2-13 | 4 | 0 | 2 | 38 | 62 | 43 |
| 776 | 2018-2-14 | 6 | 1 | 2 | 31 | 66 | 38 |
| 777 | 2018-2-15 | 7 | 4 | 1 | 21 | 35 | 25 |
| 778 | 2018-2-16 | 5 | 1 | 1 | 26 | 38 | 40 |
| 779 | 2018-2-17 | 8 | 3 | 4 | 29 | 45 | 50 |
| 780 | 2018-2-18 | 6 | 2 | 3 | 22 | 35 | 24 |
| 781 | 2018-2-19 | 6 | 3 | 1 | 22 | 32 | 18 |
| 782 | 2018-2-20 | 6 | 2 | 1 | 25 | 37 | 24 |
| 783 | 2018-2-21 | 25 | 11 | 4 | 30 | 51 | 40 |
| 784 | 2018-2-22 | 21 | 6 | 7 | 24 | 42 | 21 |
| 785 | 2018-2-23 | 21 | 7 | 7 | 26 | 52 | 38 |
| 786 | 2018-2-24 | 9 | 4 | 1 | 29 | 50 | 28 |
| 787 | 2018-2-25 | 7 | 2 | 2 | 37 | 62 | 38 |
| 788 | 2018-2-26 | 28 | 7 | 8 | 46 | 80 | 65 |
| 789 | 2018-2-27 | 22 | 3 | 6 | 26 | 44 | 21 |
| 790 | 2018-2-28 | 26 | 10 | 4 | 31 | 58 | 56 |
| 791 | 2018-3-1 | 18 | 4 | 3 | 27 | 52 | 34 |
| 792 | 2018-3-2 | 13 | 5 | 3 | 26 | 47 | 26 |

| | | | | | | | |
|---|---|---|---|---|---|---|---|
| 793 | 2018-3-3 | 16 | 7 | 2 | 28 | 51 | 27 |
| 794 | 2018-3-4 | 13 | 3 | 4 | 23 | 46 | 40 |
| 795 | 2018-3-5 | 32 | 6 | 12 | 31 | 54 | 23 |
| 796 | 2018-3-6 | 23 | 5 | 2 | 32 | 62 | 29 |
| 797 | 2018-3-7 | 34 | 10 | 4 | 28 | 45 | 36 |
| 798 | 2018-3-8 | 17 | 4 | 5 | 15 | 33 | 26 |
| 799 | 2018-3-9 | 13 | 5 | 2 | 41 | 66 | 34 |
| 800 | 2018-3-10 | 9 | 1 | 2 | 49 | 72 | 31 |
| 801 | 2018-3-11 | 6 | 2 | 1 | 43 | 70 | 37 |
| 802 | 2018-3-12 | 34 | 12 | 7 | 40 | 67 | 54 |
| 803 | 2018-3-13 | 31 | 11 | 3 | 36 | 65 | 46 |
| 804 | 2018-3-14 | 22 | 9 | 4 | 31 | 65 | 45 |
| 805 | 2018-3-15 | 23 | 4 | 7 | 26 | 44 | 31 |
| 806 | 2018-3-16 | 14 | 4 | 2 | 42 | 70 | 52 |
| 807 | 2018-3-17 | 10 | 1 | 5 | 39 | 62 | 78 |
| 808 | 2018-3-18 | 11 | 3 | 4 | 29 | 49 | 36 |
| 809 | 2018-3-19 | 38 | 13 | 10 | 22 | 39 | 29 |
| 810 | 2018-3-20 | 19 | 6 | 8 | 29 | 54 | 45 |
| 811 | 2018-3-21 | 17 | 4 | 4 | 39 | 68 | 29 |
| 812 | 2018-3-22 | 16 | 6 | 4 | 39 | 67 | 30 |
| 813 | 2018-3-23 | 12 | 4 | 1 | 49 | 77 | 34 |
| 814 | 2018-3-24 | 14 | 5 | 5 | 40 | 60 | 38 |
| 815 | 2018-3-25 | 7 | 5 | 1 | 41 | 63 | 33 |
| 816 | 2018-3-26 | 25 | 7 | 3 | 47 | 78 | 32 |
| 817 | 2018-3-27 | 22 | 11 | 5 | 33 | 50 | 17 |
| 818 | 2018-3-28 | 19 | 5 | 6 | 24 | 38 | 21 |
| 819 | 2018-3-29 | 18 | 4 | 4 | 32 | 52 | 32 |
| 820 | 2018-3-30 | 12 | 1 | 3 | 40 | 62 | 30 |
| 821 | 2018-3-31 | 10 | 7 | 0 | 38 | 67 | 32 |
| 822 | 2018-4-1 | 4 | 4 | 0 | 33 | 64 | 42 |
| 823 | 2018-4-2 | 24 | 9 | 6 | 41 | 78 | 38 |
| 824 | 2018-4-3 | 14 | 6 | 2 | 39 | 71 | 35 |

| | | | | | | | |
|---|---|---|---|---|---|---|---|
| 825 | 2018-4-4 | 7 | 4 | 0 | 43 | 80 | 40 |
| 826 | 2018-4-5 | 5 | 2 | 0 | 30 | 51 | 24 |
| 827 | 2018-4-6 | 9 | 4 | 3 | 19 | 40 | 16 |
| 828 | 2018-4-7 | 6 | 1 | 3 | 19 | 38 | 38 |
| 829 | 2018-4-8 | 23 | 4 | 5 | 34 | 64 | 47 |
| 830 | 2018-4-9 | 26 | 10 | 7 | 42 | 72 | 51 |
| 831 | 2018-4-10 | 17 | 6 | 4 | 23 | 45 | 31 |
| 832 | 2018-4-11 | 17 | 6 | 4 | 19 | 36 | 26 |
| 833 | 2018-4-12 | 16 | 8 | 4 | 20 | 38 | 24 |
| 834 | 2018-4-13 | 23 | 11 | 2 | 21 | 37 | 32 |
| 835 | 2018-4-14 | 5 | 2 | 1 | 23 | 39 | 22 |
| 836 | 2018-4-15 | 5 | 3 | 0 | 26 | 51 | 38 |
| 837 | 2018-4-16 | 34 | 12 | 12 | 20 | 37 | 26 |
| 838 | 2018-4-17 | 29 | 6 | 9 | 28 | 51 | 31 |
| 839 | 2018-4-18 | 20 | 10 | 6 | 30 | 51 | 32 |
| 840 | 2018-4-19 | 24 | 8 | 8 | 29 | 44 | 26 |
| 841 | 2018-4-20 | 19 | 3 | 5 | 25 | 46 | 24 |
| 842 | 2018-4-21 | 3 | 2 | 1 | 20 | 36 | 25 |
| 843 | 2018-4-22 | 5 | 3 | 1 | 24 | 46 | 27 |
| 844 | 2018-4-23 | 27 | 8 | 6 | 31 | 57 | 32 |
| 845 | 2018-4-24 | 24 | 11 | 6 | 35 | 69 | 35 |
| 846 | 2018-4-25 | 22 | 8 | 7 | 30 | 57 | 23 |
| 847 | 2018-4-26 | 11 | 4 | 4 | 33 | 60 | 24 |
| 848 | 2018-4-27 | 10 | 5 | 1 | 31 | 54 | 24 |
| 849 | 2018-4-28 | 12 | 8 | 1 | 37 | 55 | 30 |
| 850 | 2018-4-29 | 8 | 5 | 3 | 39 | 55 | 27 |
| 851 | 2018-4-30 | 8 | 0 | 4 | 35 | 52 | 24 |
| 852 | 2018-5-1 | 6 | 4 | 0 | 29 | 49 | 27 |
| 853 | 2018-5-2 | 31 | 12 | 6 | 30 | 51 | 32 |
| 854 | 2018-5-3 | 29 | 10 | 8 | 40 | 61 | 36 |
| 855 | 2018-5-4 | 14 | 5 | 3 | 46 | 69 | 49 |
| 856 | 2018-5-5 | 6 | 4 | 0 | 15 | 35 | 23 |

| | | | | | | | |
|---|---|---|---|---|---|---|---|
| 857 | 2018-5-6 | 11 | 7 | 3 | 16 | 36 | 24 |
| 858 | 2018-5-7 | 30 | 10 | 10 | 15 | 29 | 22 |
| 859 | 2018-5-8 | 23 | 11 | 7 | 18 | 34 | 22 |
| 860 | 2018-5-9 | 19 | 9 | 6 | 23 | 46 | 26 |
| 861 | 2018-5-10 | 28 | 7 | 10 | 21 | 42 | 27 |
| 862 | 2018-5-11 | 11 | 3 | 2 | 22 | 45 | 18 |
| 863 | 2018-5-12 | 4 | 2 | 1 | 25 | 50 | 24 |
| 864 | 2018-5-13 | 9 | 3 | 4 | 24 | 39 | 21 |
| 865 | 2018-5-14 | 37 | 19 | 8 | 17 | 32 | 30 |
| 866 | 2018-5-15 | 29 | 11 | 6 | 20 | 40 | 25 |
| 867 | 2018-5-16 | 19 | 10 | 4 | 19 | 35 | 23 |
| 868 | 2018-5-17 | 13 | 3 | 1 | 23 | 45 | 20 |
| 869 | 2018-5-18 | 8 | 3 | 2 | 25 | 47 | 21 |
| 870 | 2018-5-19 | 10 | 3 | 5 | 27 | 50 | 26 |
| 871 | 2018-5-20 | 7 | 3 | 3 | 25 | 44 | 20 |
| 872 | 2018-5-21 | 17 | 5 | 6 | 23 | 39 | 20 |
| 873 | 2018-5-22 | 13 | 5 | 0 | 18 | 33 | 23 |
| 874 | 2018-5-23 | 15 | 4 | 4 | 21 | 34 | 33 |
| 875 | 2018-5-24 | 14 | 5 | 2 | 19 | 36 | 58 |
| 876 | 2018-5-25 | 10 | 4 | 5 | 14 | 27 | 15 |
| 877 | 2018-5-26 | 11 | 9 | 1 | 17 | 30 | 19 |
| 878 | 2018-5-27 | 6 | 3 | 1 | 14 | 26 | 20 |
| 879 | 2018-5-28 | 17 | 8 | 3 | 30 | 55 | 38 |
| 880 | 2018-5-29 | 18 | 3 | 3 | 35 | 59 | 44 |
| 881 | 2018-5-30 | 25 | 12 | 2 | 15 | 31 | 32 |
| 882 | 2018-5-31 | 19 | 6 | 6 | 14 | 28 | 31 |
| 883 | 2018-6-1 | 12 | 6 | 1 | 28 | 42 | 60 |
| 884 | 2018-6-2 | 10 | 3 | 4 | 16 | 36 | 35 |
| 885 | 2018-6-3 | 2 | 1 | 1 | 23 | 43 | 59 |
| 886 | 2018-6-4 | 18 | 6 | 6 | 14 | 30 | 38 |
| 887 | 2018-6-5 | 15 | 1 | 5 | 22 | 36 | 36 |
| 888 | 2018-6-6 | 23 | 7 | 7 | 27 | 50 | 37 |

| | | | | | | | |
|---|---|---|---|---|---|---|---|
| 889 | 2018-6-7 | 22 | 3 | 5 | 30 | 54 | 28 |
| 890 | 2018-6-8 | 9 | 2 | 4 | 32 | 55 | 27 |
| 891 | 2018-6-9 | 6 | 3 | 1 | 20 | 39 | 37 |
| 892 | 2018-6-10 | 6 | 1 | 2 | 22 | 40 | 33 |
| 893 | 2018-6-11 | 26 | 9 | 11 | 13 | 28 | 32 |
| 894 | 2018-6-12 | 29 | 13 | 5 | 10 | 29 | 48 |
| 895 | 2018-6-13 | 18 | 8 | 2 | 15 | 30 | 34 |
| 896 | 2018-6-14 | 18 | 8 | 4 | 12 | 24 | 26 |
| 897 | 2018-6-15 | 12 | 8 | 3 | 17 | 36 | 36 |
| 898 | 2018-6-16 | 2 | 0 | 1 | 38 | 71 | 75 |
| 899 | 2018-6-17 | 10 | 6 | 0 | 28 | 60 | 34 |
| 900 | 2018-6-18 | 7 | 4 | 1 | 23 | 52 | 22 |
| 901 | 2018-6-19 | 24 | 5 | 4 | 29 | 65 | 30 |
| 902 | 2018-6-20 | 24 | 8 | 8 | 27 | 56 | 37 |
| 903 | 2018-6-21 | 18 | 7 | 3 | 25 | 48 | 26 |
| 904 | 2018-6-22 | 14 | 5 | 4 | 13 | 30 | 41 |
| 905 | 2018-6-23 | 8 | 3 | 0 | 26 | 50 | 96 |
| 906 | 2018-6-24 | 14 | 6 | 4 | 18 | 38 | 32 |
| 907 | 2018-6-25 | 32 | 13 | 11 | 19 | 36 | 34 |
| 908 | 2018-6-26 | 17 | 5 | 9 | 15 | 37 | 50 |
| 909 | 2018-6-27 | 15 | 4 | 5 | 16 | 35 | 26 |
| 910 | 2018-6-28 | 14 | 3 | 3 | 19 | 42 | 27 |
| 911 | 2018-6-29 | 8 | 0 | 5 | 17 | 36 | 28 |
| 912 | 2018-6-30 | 9 | 5 | 0 | 23 | 48 | 55 |
| 913 | 2018-7-1 | 16 | 7 | 8 | 25 | 45 | 36 |
| 914 | 2018-7-2 | 30 | 12 | 5 | 18 | 38 | 28 |
| 915 | 2018-7-3 | 25 | 6 | 8 | 14 | 30 | 26 |
| 916 | 2018-7-4 | 13 | 4 | 3 | 14 | 31 | 28 |
| 917 | 2018-7-5 | 17 | 3 | 4 | 13 | 27 | 27 |
| 918 | 2018-7-6 | 8 | 1 | 4 | 17 | 33 | 25 |
| 919 | 2018-7-7 | 10 | 3 | 2 | 20 | 34 | 37 |
| 920 | 2018-7-8 | 4 | 2 | 1 | 26 | 43 | 48 |

| | | | | | | | |
|---|---|---|---|---|---|---|---|
| 921 | 2018-7-9 | 29 | 11 | 7 | 28 | 47 | 57 |
| 922 | 2018-7-10 | 20 | 9 | 4 | 26 | 48 | 56 |
| 923 | 2018-7-11 | 23 | 9 | 4 | 22 | 45 | 50 |
| 924 | 2018-7-12 | 21 | 5 | 6 | 17 | 30 | 29 |
| 925 | 2018-7-13 | 18 | 8 | 2 | 18 | 36 | 26 |
| 926 | 2018-7-14 | 9 | 6 | 3 | 21 | 42 | 28 |
| 927 | 2018-7-15 | 5 | 4 | 0 | 19 | 37 | 35 |
| 928 | 2018-7-16 | 37 | 12 | 7 | 26 | 44 | 44 |
| 929 | 2018-7-17 | 20 | 8 | 3 | 25 | 43 | 33 |
| 930 | 2018-7-18 | 11 | 2 | 3 | 27 | 46 | 46 |
| 931 | 2018-7-19 | 13 | 6 | 3 | 28 | 49 | 35 |
| 932 | 2018-7-20 | 13 | 8 | 1 | 27 | 47 | 53 |
| 933 | 2018-7-21 | 10 | 5 | 1 | 28 | 49 | 50 |
| 934 | 2018-7-22 | 16 | 7 | 5 | 27 | 46 | 32 |
| 935 | 2018-7-23 | 29 | 10 | 4 | 32 | 52 | 39 |
| 936 | 2018-7-24 | 32 | 6 | 7 | 28 | 47 | 44 |
| 937 | 2018-7-25 | 13 | 6 | 1 | 40 | 66 | 55 |
| 938 | 2018-7-26 | 19 | 6 | 4 | 34 | 51 | 41 |
| 939 | 2018-7-27 | 16 | 5 | 3 | 27 | 45 | 52 |
| 940 | 2018-7-28 | 9 | 4 | 3 | 26 | 45 | 39 |
| 941 | 2018-7-29 | 10 | 5 | 2 | 18 | 32 | 28 |
| 942 | 2018-7-30 | 28 | 6 | 6 | 21 | 40 | 36 |
| 943 | 2018-7-31 | 16 | 7 | 7 | 20 | 38 | 34 |
| 944 | 2018-8-1 | 25 | 9 | 4 | 21 | 38 | 40 |
| 945 | 2018-8-2 | 14 | 7 | 2 | 18 | 39 | 28 |
| 946 | 2018-8-3 | 18 | 5 | 5 | 12 | 27 | 26 |
| 947 | 2018-8-4 | 6 | 1 | 2 | 22 | 46 | 58 |
| 948 | 2018-8-5 | 7 | 2 | 3 | 20 | 46 | 39 |
| 949 | 2018-8-6 | 34 | 10 | 11 | 27 | 49 | 54 |
| 950 | 2018-8-7 | 22 | 11 | 5 | 23 | 43 | 31 |
| 951 | 2018-8-8 | 24 | 8 | 6 | 27 | 50 | 56 |
| 952 | 2018-8-9 | 25 | 7 | 8 | 18 | 35 | 30 |

| | | | | | | | |
|---|---|---|---|---|---|---|---|
| 953 | 2018-8-10 | 15 | 10 | 1 | 25 | 43 | 38 |
| 954 | 2018-8-11 | 10 | 5 | 1 | 20 | 35 | 22 |
| 955 | 2018-8-12 | 17 | 5 | 3 | 24 | 42 | 31 |
| 956 | 2018-8-13 | 27 | 8 | 7 | 26 | 42 | 32 |
| 957 | 2018-8-14 | 24 | 5 | 5 | 29 | 46 | 49 |
| 958 | 2018-8-15 | 17 | 4 | 4 | 27 | 47 | 39 |
| 959 | 2018-8-16 | 20 | 9 | 2 | 31 | 57 | 45 |
| 960 | 2018-8-17 | 23 | 10 | 8 | 29 | 52 | 41 |
| 961 | 2018-8-18 | 7 | 5 | 1 | 28 | 48 | 36 |
| 962 | 2018-8-19 | 6 | 1 | 1 | 34 | 58 | 43 |
| 963 | 2018-8-20 | 29 | 9 | 10 | 25 | 42 | 30 |
| 964 | 2018-8-21 | 20 | 8 | 7 | 31 | 50 | 42 |
| 965 | 2018-8-22 | 22 | 10 | 2 | 24 | 41 | 36 |
| 966 | 2018-8-23 | 22 | 12 | 5 | 22 | 37 | 33 |
| 967 | 2018-8-24 | 15 | 3 | 0 | 38 | 65 | 51 |
| 968 | 2018-8-25 | 8 | 2 | 2 | 38 | 58 | 35 |
| 969 | 2018-8-26 | 5 | 2 | 1 | 35 | 55 | 38 |
| 970 | 2018-8-27 | 26 | 6 | 9 | 41 | 60 | 29 |
| 971 | 2018-8-28 | 12 | 5 | 3 | 48 | 68 | 31 |
| 972 | 2018-8-29 | 20 | 8 | 4 | 50 | 71 | 43 |
| 973 | 2018-8-30 | 12 | 7 | 2 | 34 | 53 | 27 |
| 974 | 2018-8-31 | 12 | 2 | 4 | 29 | 48 | 23 |
| 975 | 2018-9-1 | 9 | 7 | 1 | 28 | 47 | 44 |
| 976 | 2018-9-2 | 6 | 2 | 1 | 29 | 48 | 29 |
| 977 | 2018-9-3 | 25 | 5 | 9 | 33 | 50 | 40 |
| 978 | 2018-9-4 | 21 | 9 | 6 | 27 | 40 | 51 |
| 979 | 2018-9-5 | 15 | 6 | 4 | 30 | 47 | 30 |
| 980 | 2018-9-6 | 9 | 4 | 3 | 26 | 41 | 28 |
| 981 | 2018-9-7 | 11 | 1 | 3 | 15 | 29 | 17 |
| 982 | 2018-9-8 | 9 | 5 | 1 | 19 | 34 | 14 |
| 983 | 2018-9-9 | 4 | 3 | 1 | 32 | 49 | 23 |
| 984 | 2018-9-10 | 24 | 9 | 7 | 41 | 64 | 46 |

| | | | | | | | |
|---|---|---|---|---|---|---|---|
| 985 | 2018-9-11 | 26 | 5 | 12 | 21 | 38 | 38 |
| 986 | 2018-9-12 | 14 | 4 | 4 | 19 | 32 | 28 |
| 987 | 2018-9-13 | 18 | 6 | 5 | 22 | 39 | 52 |
| 988 | 2018-9-14 | 14 | 3 | 6 | 26 | 47 | 57 |
| 989 | 2018-9-15 | 3 | 3 | 0 | 43 | 65 | 32 |
| 990 | 2018-9-16 | 7 | 3 | 3 | 34 | 53 | 29 |
| 991 | 2018-9-17 | 33 | 12 | 11 | 29 | 48 | 24 |
| 992 | 2018-9-18 | 33 | 13 | 5 | 22 | 42 | 50 |
| 993 | 2018-9-19 | 15 | 7 | 5 | 32 | 51 | 54 |
| 994 | 2018-9-20 | 17 | 9 | 4 | 26 | 45 | 43 |
| 995 | 2018-9-21 | 11 | 4 | 1 | 19 | 38 | 30 |
| 996 | 2018-9-22 | 4 | 1 | 0 | 23 | 41 | 39 |
| 997 | 2018-9-23 | 5 | 2 | 3 | 41 | 63 | 95 |
| 998 | 2018-9-24 | 8 | 4 | 2 | 27 | 46 | 54 |
| 999 | 2018-9-25 | 22 | 10 | 3 | 21 | 38 | 34 |
| 1000 | 2018-9-26 | 23 | 7 | 8 | 22 | 41 | 32 |
| 1001 | 2018-9-27 | 14 | 7 | 3 | 25 | 39 | 24 |
| 1002 | 2018-9-28 | 11 | 3 | 5 | 15 | 23 | 14 |
| 1003 | 2018-9-29 | 11 | 2 | 2 | 28 | 46 | 27 |
| 1004 | 2018-9-30 | 14 | 7 | 2 | 23 | 37 | 30 |
| 1005 | 2018-10-1 | 5 | 1 | 2 | 10 | 24 | 23 |
| 1006 | 2018-10-2 | 10 | 6 | 1 | 22 | 39 | 47 |
| 1007 | 2018-10-3 | 5 | 1 | 2 | 21 | 34 | 32 |
| 1008 | 2018-10-4 | 10 | 5 | 3 | 21 | 35 | 57 |
| 1009 | 2018-10-5 | 8 | 1 | 2 | 34 | 56 | 44 |
| 1010 | 2018-10-6 | 5 | 1 | 0 | 22 | 39 | 42 |
| 1011 | 2018-10-7 | 17 | 5 | 3 | 25 | 50 | 45 |
| 1012 | 2018-10-8 | 27 | 10 | 6 | 24 | 43 | 40 |
| 1013 | 2018-10-9 | 31 | 16 | 8 | 20 | 36 | 44 |
| 1014 | 2018-10-10 | 25 | 10 | 5 | 22 | 36 | 60 |
| 1015 | 2018-10-11 | 21 | 6 | 3 | 52 | 78 | 92 |
| 1016 | 2018-10-12 | 12 | 2 | 6 | 47 | 77 | 50 |

| | | | | | | | |
|---|---|---|---|---|---|---|---|
| 1017 | 2018-10-13 | 8 | 5 | 0 | 41 | 66 | 46 |
| 1018 | 2018-10-14 | 13 | 4 | 4 | 34 | 63 | 53 |
| 1019 | 2018-10-15 | 23 | 5 | 7 | 29 | 50 | 47 |
| 1020 | 2018-10-16 | 32 | 11 | 6 | 19 | 38 | 30 |
| 1021 | 2018-10-17 | 17 | 7 | 6 | 27 | 48 | 36 |
| 1022 | 2018-10-18 | 21 | 9 | 3 | 28 | 52 | 21 |
| 1023 | 2018-10-19 | 18 | 5 | 3 | 27 | 52 | 29 |
| 1024 | 2018-10-20 | 6 | 3 | 1 | 29 | 53 | 60 |
| 1025 | 2018-10-21 | 10 | 5 | 1 | 30 | 57 | 47 |
| 1026 | 2018-10-22 | 28 | 8 | 6 | 31 | 56 | 48 |
| 1027 | 2018-10-23 | 22 | 7 | 9 | 33 | 59 | 46 |
| 1028 | 2018-10-24 | 12 | 3 | 2 | 26 | 46 | 66 |
| 1029 | 2018-10-25 | 17 | 6 | 8 | 29 | 56 | 56 |
| 1030 | 2018-10-26 | 9 | 3 | 2 | 29 | 60 | 44 |
| 1031 | 2018-10-27 | 5 | 2 | 3 | 36 | 61 | 38 |
| 1032 | 2018-10-28 | 15 | 7 | 6 | 34 | 60 | 32 |
| 1033 | 2018-10-29 | 38 | 16 | 10 | 37 | 58 | 27 |
| 1034 | 2018-10-30 | 22 | 4 | 2 | 24 | 40 | 30 |
| 1035 | 2018-10-31 | 19 | 8 | 6 | 20 | 39 | 23 |
| 1036 | 2018-11-1 | 15 | 1 | 6 | 30 | 56 | 39 |
| 1037 | 2018-11-2 | 21 | 11 | 3 | 34 | 57 | 32 |
| 1038 | 2018-11-3 | 11 | 5 | 2 | 38 | 62 | 35 |
| 1039 | 2018-11-4 | 8 | 3 | 1 | 29 | 52 | 29 |
| 1040 | 2018-11-5 | 21 | 6 | 5 | 30 | 54 | 34 |
| 1041 | 2018-11-6 | 21 | 6 | 8 | 31 | 53 | 35 |
| 1042 | 2018-11-7 | 11 | 7 | 1 | 30 | 52 | 32 |
| 1043 | 2018-11-8 | 18 | 6 | 4 | 26 | 44 | 21 |
| 1044 | 2018-11-9 | 16 | 6 | 5 | 36 | 58 | 32 |
| 1045 | 2018-11-10 | 1 | 0 | 1 | 37 | 61 | 42 |
| 1046 | 2018-11-11 | 5 | 2 | 0 | 27 | 49 | 38 |
| 1047 | 2018-11-12 | 20 | 4 | 6 | 26 | 48 | 38 |
| 1048 | 2018-11-13 | 21 | 1 | 5 | 26 | 47 | 33 |

| | | | | | | | |
|---|---|---|---|---|---|---|---|
| 1049 | 2018-11-14 | 14 | 4 | 7 | 24 | 47 | 37 |
| 1050 | 2018-11-15 | 19 | 3 | 6 | 29 | 51 | 43 |
| 1051 | 2018-11-16 | 6 | 1 | 2 | 33 | 61 | 35 |
| 1052 | 2018-11-17 | 5 | 2 | 2 | 34 | 62 | 33 |
| 1053 | 2018-11-18 | 6 | 3 | 3 | 40 | 65 | 28 |
| 1054 | 2018-11-19 | 21 | 3 | 7 | 44 | 70 | 26 |
| 1055 | 2018-11-20 | 13 | 7 | 3 | 37 | 63 | 29 |
| 1056 | 2018-11-21 | 10 | 3 | 2 | 35 | 63 | 30 |
| 1057 | 2018-11-22 | 7 | 3 | 0 | 35 | 57 | 23 |
| 1058 | 2018-11-23 | 6 | 3 | 2 | 36 | 58 | 23 |
| 1059 | 2018-11-24 | 5 | 3 | 0 | 31 | 55 | 20 |
| 1060 | 2018-11-25 | 2 | 0 | 2 | 31 | 53 | 25 |
| 1061 | 2018-11-26 | 11 | 5 | 4 | 39 | 64 | 27 |
| 1062 | 2018-11-27 | 11 | 2 | 3 | 36 | 60 | 26 |
| 1063 | 2018-11-28 | 8 | 2 | 2 | 34 | 56 | 24 |
| 1064 | 2018-11-29 | 6 | 2 | 3 | 40 | 63 | 24 |
| 1065 | 2018-11-30 | 3 | 1 | 2 | 33 | 57 | 23 |
| 1066 | 2018-12-1 | 6 | 3 | 2 | 25 | 47 | 25 |
| 1067 | 2018-12-2 | 3 | 1 | 0 | 21 | 45 | 27 |
| 1068 | 2018-12-3 | 6 | 0 | 2 | 22 | 46 | 31 |
| 1069 | 2018-12-4 | 14 | 3 | 4 | 26 | 50 | 35 |
| 1070 | 2018-12-5 | 7 | 2 | 2 | 30 | 64 | 24 |
| 1071 | 2018-12-6 | 9 | 2 | 4 | 29 | 58 | 29 |
| 1072 | 2018-12-7 | 4 | 2 | 1 | 26 | 52 | 38 |
| 1073 | 2018-12-8 | 4 | 2 | 2 | 26 | 50 | 43 |
| 1074 | 2018-12-9 | 0 | 0 | 0 | 23 | 45 | 40 |
| 1075 | 2018-12-10 | 2 | 0 | 1 | 23 | 45 | 35 |
| 1076 | 2018-12-11 | 8 | 3 | 2 | 25 | 49 | 35 |
| 1077 | 2018-12-12 | 2 | 2 | 0 | 28 | 51 | 30 |
| 1078 | 2018-12-13 | 1 | 1 | 0 | 30 | 53 | 30 |
| 1079 | 2018-12-14 | 2 | 1 | 1 | 32 | 64 | 38 |
| 1080 | 2018-12-15 | 2 | 2 | 0 | 31 | 59 | 40 |

| | | | | | | | |
|---|---|---|---|---|---|---|---|
| 1081 | 2018-12-16 | 1 | 1 | 0 | 31 | 63 | 38 |
| 1082 | 2018-12-17 | 5 | 2 | 0 | 38 | 67 | 41 |
| 1083 | 2018-12-18 | 4 | 2 | 0 | 49 | 81 | 38 |
| 1084 | 2018-12-19 | 4 | 2 | 2 | 50 | 78 | 34 |
| 1085 | 2018-12-20 | 3 | 1 | 1 | 20 | 36 | 22 |
| 1086 | 2018-12-21 | 7 | 5 | 0 | 23 | 44 | 27 |
| 1087 | 2018-12-22 | 3 | 2 | 0 | 27 | 51 | 37 |
| 1088 | 2018-12-23 | 4 | 2 | 1 | 24 | 43 | 25 |
| 1089 | 2018-12-24 | 9 | 1 | 3 | 25 | 45 | 26 |
| 1090 | 2018-12-25 | 9 | 5 | 1 | 26 | 50 | 30 |
| 1091 | 2018-12-26 | 16 | 4 | 5 | 27 | 50 | 36 |
| 1092 | 2018-12-27 | 12 | 4 | 5 | 28 | 52 | 35 |
| 1093 | 2018-12-28 | 15 | 8 | 3 | 36 | 60 | 37 |
| 1094 | 2018-12-29 | 11 | 4 | 1 | 33 | 55 | 59 |
| 1095 | 2018-12-30 | 2 | 1 | 1 | 30 | 44 | 26 |
| 1096 | 2018-12-31 | 3 | 0 | 2 | 33 | 51 | 28 |
| 1097 | 2019-1-1 | 6 | 1 | 3 | 37 | 59 | 36 |
| 1098 | 2019-1-2 | 21 | 9 | 3 | 36 | 56 | 32 |
| 1099 | 2019-1-3 | 24 | 8 | 5 | 40 | 57 | 39 |
| 1100 | 2019-1-4 | 23 | 9 | 7 | 40 | 54 | 43 |
| 1101 | 2019-1-5 | 7 | 2 | 3 | 50 | 78 | 52 |
| 1102 | 2019-1-6 | 11 | 3 | 3 | 84 | 105 | 53 |
| 1103 | 2019-1-7 | 31 | 10 | 8 | 80 | 109 | 60 |
| 1104 | 2019-1-8 | 24 | 5 | 9 | 36 | 57 | 36 |
| 1105 | 2019-1-9 | 19 | 7 | 6 | 21 | 32 | 21 |
| 1106 | 2019-1-10 | 16 | 6 | 4 | 23 | 40 | 19 |
| 1107 | 2019-1-11 | 19 | 6 | 8 | 46 | 70 | 25 |
| 1108 | 2019-1-12 | 10 | 3 | 4 | 61 | 94 | 41 |
| 1109 | 2019-1-13 | 6 | 3 | 1 | 54 | 86 | 46 |
| 1110 | 2019-1-14 | 38 | 8 | 13 | 50 | 86 | 58 |
| 1111 | 2019-1-15 | 20 | 6 | 5 | 40 | 65 | 46 |
| 1112 | 2019-1-16 | 13 | 3 | 3 | 37 | 61 | 37 |

| | | | | | | | |
|---|---|---|---|---|---|---|---|
| 1113 | 2019-1-17 | 21 | 4 | 5 | 58 | 86 | 40 |
| 1114 | 2019-1-18 | 15 | 5 | 5 | 53 | 88 | 49 |
| 1115 | 2019-1-19 | 11 | 5 | 1 | 29 | 55 | 40 |
| 1116 | 2019-1-20 | 10 | 2 | 2 | 40 | 75 | 54 |
| 1117 | 2019-1-21 | 24 | 6 | 7 | 42 | 77 | 38 |
| 1118 | 2019-1-22 | 19 | 7 | 5 | 46 | 80 | 41 |
| 1119 | 2019-1-23 | 16 | 7 | 5 | 46 | 83 | 46 |
| 1120 | 2019-1-24 | 11 | 2 | 5 | 33 | 68 | 44 |
| 1121 | 2019-1-25 | 11 | 4 | 3 | 36 | 68 | 42 |
| 1122 | 2019-1-26 | 8 | 3 | 3 | 39 | 71 | 38 |
| 1123 | 2019-1-27 | 7 | 5 | 0 | 33 | 62 | 31 |
| 1124 | 2019-1-28 | 18 | 7 | 2 | 28 | 55 | 30 |
| 1125 | 2019-1-29 | 15 | 5 | 7 | 28 | 47 | 16 |
| 1126 | 2019-1-30 | 13 | 4 | 5 | 14 | 28 | 16 |
| 1127 | 2019-1-31 | 9 | 2 | 2 | 24 | 39 | 30 |
| 1128 | 2019-2-1 | 4 | 1 | 2 | 36 | 56 | 38 |
| 1129 | 2019-2-2 | 6 | 3 | 2 | 28 | 52 | 46 |
| 1130 | 2019-2-3 | 7 | 1 | 2 | 17 | 30 | 24 |
| 1131 | 2019-2-4 | 4 | 1 | 1 | 17 | 28 | 27 |
| 1132 | 2019-2-5 | 6 | 3 | 2 | 25 | 35 | 35 |
| 1133 | 2019-2-6 | 4 | 1 | 2 | 28 | 48 | 71 |
| 1134 | 2019-2-7 | 13 | 3 | 3 | 19 | 32 | 29 |
| 1135 | 2019-2-8 | 12 | 5 | 2 | 29 | 51 | 54 |
| 1136 | 2019-2-9 | 8 | 5 | 1 | 18 | 34 | 20 |
| 1137 | 2019-2-10 | 27 | 6 | 8 | 18 | 32 | 23 |
| 1138 | 2019-2-11 | 30 | 6 | 10 | 17 | 26 | 21 |
| 1139 | 2019-2-12 | 38 | 5 | 15 | 22 | 38 | 41 |
| 1140 | 2019-2-13 | 12 | 5 | 3 | 23 | 42 | 47 |
| 1141 | 2019-2-14 | 20 | 9 | 6 | 27 | 51 | 59 |
| 1142 | 2019-2-15 | 25 | 8 | 6 | 23 | 48 | 47 |
| 1143 | 2019-2-16 | 5 | 2 | 1 | 19 | 37 | 40 |
| 1144 | 2019-2-17 | 9 | 2 | 2 | 19 | 34 | 30 |

| | | | | | | | |
|---|---|---|---|---|---|---|---|
| 1145 | 2019-2-18 | 30 | 7 | 6 | 14 | 27 | 23 |
| 1146 | 2019-2-19 | 14 | 3 | 3 | 18 | 32 | 24 |
| 1147 | 2019-2-20 | 28 | 13 | 7 | 21 | 37 | 25 |
| 1148 | 2019-2-21 | 26 | 9 | 8 | 29 | 49 | 29 |
| 1149 | 2019-2-22 | 19 | 4 | 3 | 36 | 62 | 26 |
| 1150 | 2019-2-23 | 4 | 1 | 0 | 36 | 63 | 25 |
| 1151 | 2019-2-24 | 12 | 6 | 3 | 28 | 51 | 23 |
| 1152 | 2019-2-25 | 36 | 10 | 7 | 23 | 41 | 29 |
| 1153 | 2019-2-26 | 15 | 3 | 3 | 11 | 24 | 18 |
| 1154 | 2019-2-27 | 22 | 6 | 6 | 12 | 26 | 18 |
| 1155 | 2019-2-28 | 24 | 5 | 8 | 12 | 25 | 14 |
| 1156 | 2019-3-1 | 19 | 9 | 6 | 17 | 37 | 32 |
| 1157 | 2019-3-2 | 8 | 3 | 3 | 18 | 35 | 33 |
| 1158 | 2019-3-3 | 8 | 3 | 1 | 26 | 52 | 45 |
| 1159 | 2019-3-4 | 46 | 12 | 17 | 39 | 77 | 66 |
| 1160 | 2019-3-5 | 19 | 7 | 7 | 32 | 59 | 32 |
| 1161 | 2019-3-6 | 22 | 9 | 5 | 20 | 35 | 20 |
| 1162 | 2019-3-7 | 16 | 9 | 3 | 23 | 40 | 20 |
| 1163 | 2019-3-8 | 12 | 3 | 5 | 22 | 39 | 21 |
| 1164 | 2019-3-9 | 6 | 3 | 0 | 22 | 37 | 15 |
| 1165 | 2019-3-10 | 6 | 4 | 1 | 29 | 54 | 23 |
| 1166 | 2019-3-11 | 40 | 11 | 11 | 32 | 50 | 21 |
| 1167 | 2019-3-12 | 25 | 12 | 9 | 37 | 64 | 23 |
| 1168 | 2019-3-13 | 21 | 12 | 3 | 37 | 61 | 24 |
| 1169 | 2019-3-14 | 19 | 7 | 7 | 46 | 81 | 27 |
| 1170 | 2019-3-15 | 13 | 6 | 4 | 51 | 89 | 30 |
| 1171 | 2019-3-16 | 7 | 5 | 1 | 35 | 77 | 43 |
| 1172 | 2019-3-17 | 15 | 8 | 2 | 32 | 65 | 30 |
| 1173 | 2019-3-18 | 40 | 18 | 9 | 31 | 57 | 22 |
| 1174 | 2019-3-19 | 29 | 4 | 9 | 25 | 52 | 20 |
| 1175 | 2019-3-20 | 16 | 7 | 5 | 26 | 50 | 36 |
| 1176 | 2019-3-21 | 17 | 8 | 3 | 18 | 38 | 23 |

| | | | | | | | |
|---|---|---|---|---|---|---|---|
| 1177 | 2019-3-22 | 14 | 3 | 5 | 23 | 42 | 25 |
| 1178 | 2019-3-23 | 7 | 5 | 1 | 33 | 57 | 20 |
| 1179 | 2019-3-24 | 8 | 4 | 2 | 27 | 48 | 26 |
| 1180 | 2019-3-25 | 27 | 8 | 15 | 35 | 69 | 35 |
| 1181 | 2019-3-26 | 18 | 10 | 4 | 34 | 60 | 28 |
| 1182 | 2019-3-27 | 19 | 9 | 4 | 47 | 83 | 18 |
| 1183 | 2019-3-28 | 14 | 5 | 4 | 34 | 55 | 39 |
| 1184 | 2019-3-29 | 10 | 2 | 4 | 35 | 56 | 31 |
| 1185 | 2019-3-30 | 10 | 5 | 1 | 25 | 54 | 19 |
| 1186 | 2019-3-31 | 11 | 4 | 3 | 45 | 74 | 20 |
| 1187 | 2019-4-1 | 23 | 11 | 4 | 59 | 88 | 22 |
| 1188 | 2019-4-2 | 23 | 5 | 4 | 40 | 67 | 28 |
| 1189 | 2019-4-3 | 13 | 6 | 2 | 26 | 51 | 32 |
| 1190 | 2019-4-4 | 12 | 4 | 3 | 29 | 47 | 30 |
| 1191 | 2019-4-5 | 5 | 3 | 1 | 24 | 53 | 25 |
| 1192 | 2019-4-6 | 10 | 5 | 4 | 25 | 44 | 25 |
| 1193 | 2019-4-7 | 6 | 2 | 2 | 30 | 63 | 35 |
| 1194 | 2019-4-8 | 32 | 6 | 17 | 25 | 49 | 28 |
| 1195 | 2019-4-9 | 26 | 15 | 4 | 18 | 38 | 20 |
| 1196 | 2019-4-10 | 16 | 12 | 2 | 18 | 38 | 13 |
| 1197 | 2019-4-11 | 20 | 8 | 6 | 21 | 42 | 19 |
| 1198 | 2019-4-12 | 13 | 5 | 3 | 21 | 44 | 20 |
| 1199 | 2019-4-13 | 6 | 3 | 2 | 20 | 46 | 18 |
| 1200 | 2019-4-14 | 6 | 4 | 2 | 25 | 55 | 20 |
| 1201 | 2019-4-15 | 40 | 12 | 12 | 22 | 44 | 12 |
| 1202 | 2019-4-16 | 23 | 12 | 4 | 24 | 53 | 24 |
| 1203 | 2019-4-17 | 17 | 6 | 4 | 28 | 57 | 28 |
| 1204 | 2019-4-18 | 22 | 6 | 6 | 21 | 46 | 24 |
| 1205 | 2019-4-19 | 12 | 4 | 4 | 18 | 41 | 26 |
| 1206 | 2019-4-20 | 6 | 4 | 0 | 17 | 37 | 22 |
| 1207 | 2019-4-21 | 4 | 3 | 0 | 36 | 62 | 15 |
| 1208 | 2019-4-22 | 25 | 8 | 6 | 37 | 68 | 21 |

| | | | | | | | |
|---|---|---|---|---|---|---|---|
| 1209 | 2019-4-23 | 12 | 3 | 3 | 29 | 54 | 18 |
| 1210 | 2019-4-24 | 16 | 10 | 4 | 28 | 53 | 30 |
| 1211 | 2019-4-25 | 16 | 6 | 5 | 28 | 61 | 32 |
| 1212 | 2019-4-26 | 16 | 4 | 3 | 28 | 56 | 25 |
| 1213 | 2019-4-27 | 3 | 1 | 2 | 28 | 59 | 22 |
| 1214 | 2019-4-28 | 10 | 2 | 2 | 47 | 73 | 21 |
| 1215 | 2019-4-29 | 13 | 6 | 5 | 42 | 70 | 23 |
| 1216 | 2019-4-30 | 7 | 3 | 0 | 33 | 66 | 33 |
| 1217 | 2019-5-1 | 2 | 0 | 1 | 21 | 42 | 39 |
| 1218 | 2019-5-2 | 8 | 5 | 1 | 28 | 53 | 24 |
| 1219 | 2019-5-3 | 4 | 1 | 1 | 34 | 64 | 21 |
| 1220 | 2019-5-4 | 9 | 5 | 2 | 22 | 47 | 20 |
| 1221 | 2019-5-5 | 30 | 14 | 5 | 20 | 39 | 18 |
| 1222 | 2019-5-6 | 27 | 11 | 8 | 16 | 33 | 15 |
| 1223 | 2019-5-7 | 24 | 9 | 6 | 17 | 41 | 16 |
| 1224 | 2019-5-8 | 20 | 9 | 3 | 28 | 56 | 15 |
| 1225 | 2019-5-9 | 18 | 8 | 4 | 40 | 80 | 17 |
| 1226 | 2019-5-10 | 11 | 5 | 3 | 35 | 80 | 28 |
| 1227 | 2019-5-11 | 4 | 1 | 1 | 45 | 90 | 24 |
| 1228 | 2019-5-12 | 2 | 1 | 1 | 41 | 87 | 24 |
| 1229 | 2019-5-13 | 22 | 12 | 2 | 34 | 74 | 27 |
| 1230 | 2019-5-14 | 12 | 4 | 5 | 27 | 55 | 19 |
| 1231 | 2019-5-15 | 15 | 4 | 4 | 31 | 57 | 13 |
| 1232 | 2019-5-16 | 15 | 6 | 4 | 21 | 43 | 13 |
| 1233 | 2019-5-17 | 11 | 3 | 4 | 19 | 44 | 12 |
| 1234 | 2019-5-18 | 5 | 1 | 3 | 20 | 43 | 13 |
| 1235 | 2019-5-19 | 4 | 1 | 2 | 22 | 48 | 15 |
| 1236 | 2019-5-20 | 21 | 11 | 2 | 25 | 56 | 18 |
| 1237 | 2019-5-21 | 11 | 5 | 3 | 25 | 54 | 14 |
| 1238 | 2019-5-22 | 10 | 4 | 4 | 21 | 55 | 21 |
| 1239 | 2019-5-23 | 14 | 4 | 5 | 34 | 62 | 35 |
| 1240 | 2019-5-24 | 20 | 9 | 5 | 34 | 57 | 14 |

| | | | | | | | |
|---|---|---|---|---|---|---|---|
| 1241 | 2019-5-25 | 10 | 5 | 2 | 20 | 37 | 12 |
| 1242 | 2019-5-26 | 3 | 2 | 1 | 20 | 35 | 21 |
| 1243 | 2019-5-27 | 15 | 5 | 6 | 19 | 40 | 24 |
| 1244 | 2019-5-28 | 25 | 12 | 6 | 30 | 48 | 33 |
| 1245 | 2019-5-29 | 17 | 7 | 6 | 31 | 49 | 31 |
| 1246 | 2019-5-30 | 16 | 6 | 5 | 24 | 48 | 16 |
| 1247 | 2019-5-31 | 12 | 2 | 2 | 16 | 35 | 16 |
| 1248 | 2019-6-1 | 7 | 1 | 2 | 17 | 35 | 13 |
| 1249 | 2019-6-2 | 6 | 4 | 0 | 22 | 43 | 38 |
| 1250 | 2019-6-3 | 25 | 12 | 7 | 27 | 60 | 30 |
| 1251 | 2019-6-4 | 15 | 4 | 4 | 18 | 36 | 21 |
| 1252 | 2019-6-5 | 17 | 10 | 1 | 16 | 34 | 16 |
| 1253 | 2019-6-6 | 12 | 6 | 2 | 15 | 30 | 14 |
| 1254 | 2019-6-7 | 5 | 3 | 1 | 12 | 27 | 20 |
| 1255 | 2019-6-8 | 8 | 5 | 2 | 8 | 21 | 14 |
| 1256 | 2019-6-9 | 4 | 2 | 0 | 9 | 21 | 15 |
| 1257 | 2019-6-10 | 34 | 10 | 5 | 12 | 28 | 28 |
| 1258 | 2019-6-11 | 21 | 12 | 5 | 11 | 27 | 14 |
| 1259 | 2019-6-12 | 16 | 5 | 2 | 10 | 23 | 16 |
| 1260 | 2019-6-13 | 13 | 6 | 3 | 19 | 40 | 25 |
| 1261 | 2019-6-14 | 14 | 7 | 6 | 35 | 62 | 47 |
| 1262 | 2019-6-15 | 9 | 4 | 4 | 23 | 42 | 38 |
| 1263 | 2019-6-16 | 10 | 2 | 5 | 10 | 26 | 16 |
| 1264 | 2019-6-17 | 29 | 14 | 8 | 20 | 40 | 28 |
| 1265 | 2019-6-18 | 25 | 13 | 3 | 16 | 34 | 16 |
| 1266 | 2019-6-19 | 13 | 7 | 2 | 11 | 26 | 15 |
| 1267 | 2019-6-20 | 12 | 6 | 5 | 14 | 33 | 16 |
| 1268 | 2019-6-21 | 22 | 13 | 2 | 7 | 19 | 13 |
| 1269 | 2019-6-22 | 8 | 3 | 1 | 7 | 20 | 13 |
| 1270 | 2019-6-23 | 12 | 5 | 2 | 9 | 27 | 17 |
| 1271 | 2019-6-24 | 24 | 15 | 5 | 26 | 42 | 39 |
| 1272 | 2019-6-25 | 15 | 7 | 2 | 14 | 20 | 18 |

| | | | | | | | |
|---|---|---|---|---|---|---|---|
| 1273 | 2019-6-26 | 16 | 10 | 3 | 20 | 32 | 23 |
| 1274 | 2019-6-27 | 21 | 6 | 6 | 20 | 40 | 26 |
| 1275 | 2019-6-28 | 15 | 3 | 5 | 17 | 34 | 22 |
| 1276 | 2019-6-29 | 5 | 1 | 2 | 14 | 34 | 16 |
| 1277 | 2019-6-30 | 10 | 5 | 3 | 20 | 35 | 23 |
| 1278 | 2019-7-1 | 29 | 10 | 8 | 23 | 44 | 24 |
| 1279 | 2019-7-2 | 12 | 5 | 0 | 32 | 60 | 23 |
| 1280 | 2019-7-3 | 19 | 7 | 3 | 44 | 77 | 41 |
| 1281 | 2019-7-4 | 17 | 6 | 3 | 30 | 44 | 33 |
| 1282 | 2019-7-5 | 13 | 9 | 2 | 17 | 35 | 23 |
| 1283 | 2019-7-6 | 6 | 3 | 0 | 19 | 32 | 21 |
| 1284 | 2019-7-7 | 9 | 5 | 2 | 30 | 52 | 31 |
| 1285 | 2019-7-8 | 29 | 11 | 8 | 23 | 49 | 26 |
| 1286 | 2019-7-9 | 23 | 8 | 9 | 20 | 27 | 24 |
| 1287 | 2019-7-10 | 22 | 10 | 5 | 25 | 34 | 25 |
| 1288 | 2019-7-11 | 20 | 9 | 5 | 39 | 63 | 44 |
| 1289 | 2019-7-12 | 14 | 6 | 4 | 24 | 48 | 23 |
| 1290 | 2019-7-13 | 8 | 1 | 4 | 21 | 42 | 21 |
| 1291 | 2019-7-14 | 8 | 4 | 3 | 27 | 42 | 30 |
| 1292 | 2019-7-15 | 31 | 8 | 9 | 29 | 52 | 25 |
| 1293 | 2019-7-16 | 24 | 7 | 9 | 21 | 46 | 27 |
| 1294 | 2019-7-17 | 14 | 7 | 4 | 28 | 54 | 34 |
| 1295 | 2019-7-18 | 14 | 6 | 2 | 33 | 64 | 45 |
| 1296 | 2019-7-19 | 14 | 3 | 6 | 24 | 44 | 29 |
| 1297 | 2019-7-20 | 9 | 2 | 5 | 28 | 53 | 42 |
| 1298 | 2019-7-21 | 7 | 6 | 0 | 34 | 51 | 61 |
| 1299 | 2019-7-22 | 33 | 11 | 12 | 25 | 50 | 36 |
| 1300 | 2019-7-23 | 25 | 9 | 7 | 30 | 45 | 32 |
| 1301 | 2019-7-24 | 14 | 8 | 4 | 33 | 53 | 38 |
| 1302 | 2019-7-25 | 14 | 4 | 4 | 20 | 36 | 26 |
| 1303 | 2019-7-26 | 12 | 6 | 4 | 25 | 41 | 33 |
| 1304 | 2019-7-27 | 4 | 1 | 2 | 22 | 43 | 29 |

| | | | | | | | |
|---|---|---|---|---|---|---|---|
| 1305 | 2019-7-28 | 8 | 4 | 3 | 25 | 47 | 44 |
| 1306 | 2019-7-29 | 29 | 12 | 4 | 24 | 47 | 39 |
| 1307 | 2019-7-30 | 42 | 17 | 16 | 26 | 47 | 30 |
| 1308 | 2019-7-31 | 17 | 7 | 6 | 25 | 43 | 24 |
| 1309 | 2019-8-1 | 14 | 4 | 3 | 24 | 36 | 22 |
| 1310 | 2019-8-2 | 10 | 3 | 5 | 41 | 70 | 31 |
| 1311 | 2019-8-3 | 7 | 6 | 0 | 41 | 62 | 39 |
| 1312 | 2019-8-4 | 6 | 2 | 1 | 36 | 62 | 34 |
| 1313 | 2019-8-5 | 36 | 18 | 8 | 29 | 45 | 38 |
| 1314 | 2019-8-6 | 24 | 14 | 8 | 33 | 60 | 37 |
| 1315 | 2019-8-7 | 21 | 9 | 3 | 26 | 44 | 26 |
| 1316 | 2019-8-8 | 19 | 4 | 9 | 68 | 95 | 80 |
| 1317 | 2019-8-9 | 8 | 1 | 2 | 27 | 43 | 24 |
| 1318 | 2019-8-10 | 6 | 2 | 3 | 24 | 41 | 26 |
| 1319 | 2019-8-11 | 6 | 1 | 0 | 32 | 64 | 31 |
| 1320 | 2019-8-12 | 29 | 14 | 6 | 41 | 77 | 37 |
| 1321 | 2019-8-13 | 16 | 4 | 6 | 36 | 71 | 35 |
| 1322 | 2019-8-14 | 21 | 7 | 6 | 30 | 55 | 28 |
| 1323 | 2019-8-15 | 21 | 9 | 4 | 19 | 41 | 26 |
| 1324 | 2019-8-16 | 16 | 12 | 1 | 34 | 66 | 33 |
| 1325 | 2019-8-17 | 12 | 7 | 1 | 51 | 79 | 38 |
| 1326 | 2019-8-18 | 12 | 4 | 3 | 50 | 72 | 44 |
| 1327 | 2019-8-19 | 31 | 15 | 2 | 32 | 58 | 36 |
| 1328 | 2019-8-20 | 24 | 7 | 10 | 33 | 60 | 38 |
| 1329 | 2019-8-21 | 21 | 5 | 9 | 42 | 66 | 39 |
| 1330 | 2019-8-22 | 21 | 3 | 9 | 36 | 55 | 28 |
| 1331 | 2019-8-23 | 6 | 4 | 2 | 39 | 53 | 63 |
| 1332 | 2019-8-24 | 6 | 3 | 2 | 42 | 56 | 40 |
| 1333 | 2019-8-25 | 9 | 6 | 1 | 47 | 69 | 36 |
| 1334 | 2019-8-26 | 27 | 8 | 9 | 56 | 78 | 39 |
| 1335 | 2019-8-27 | 17 | 7 | 6 | 54 | 78 | 39 |
| 1336 | 2019-8-28 | 11 | 4 | 2 | 38 | 51 | 29 |

| | | | | | | | |
|---|---|---|---|---|---|---|---|
| 1337 | 2019-8-29 | 11 | 3 | 4 | 29 | 47 | 32 |
| 1338 | 2019-8-30 | 11 | 3 | 7 | 23 | 44 | 27 |
| 1339 | 2019-8-31 | 4 | 0 | 2 | 31 | 56 | 25 |
| 1340 | 2019-9-1 | 6 | 2 | 2 | 31 | 47 | 28 |
| 1341 | 2019-9-2 | 22 | 7 | 4 | 26 | 39 | 16 |
| 1342 | 2019-9-3 | 30 | 10 | 9 | 30 | 44 | 36 |
| 1343 | 2019-9-4 | 22 | 13 | 1 | 38 | 59 | 32 |
| 1344 | 2019-9-5 | 21 | 7 | 8 | 38 | 55 | 43 |
| 1345 | 2019-9-6 | 17 | 10 | 5 | 18 | 29 | 18 |
| 1346 | 2019-9-7 | 6 | 3 | 3 | 26 | 49 | 30 |
| 1347 | 2019-9-8 | 6 | 3 | 1 | 27 | 59 | 31 |
| 1348 | 2019-9-9 | 30 | 12 | 8 | 25 | 48 | 35 |
| 1349 | 2019-9-10 | 27 | 10 | 7 | 25 | 44 | 38 |
| 1350 | 2019-9-11 | 11 | 2 | 7 | 21 | 37 | 27 |
| 1351 | 2019-9-12 | 6 | 2 | 2 | 18 | 38 | 36 |
| 1352 | 2019-9-13 | 7 | 3 | 1 | 20 | 42 | 45 |
| 1353 | 2019-9-14 | 6 | 2 | 3 | 18 | 39 | 27 |
| 1354 | 2019-9-15 | 8 | 5 | 1 | 24 | 41 | 26 |
| 1355 | 2019-9-16 | 38 | 13 | 9 | 26 | 50 | 36 |
| 1356 | 2019-9-17 | 29 | 12 | 7 | 26 | 46 | 29 |
| 1357 | 2019-9-18 | 19 | 8 | 5 | 28 | 49 | 26 |
| 1358 | 2019-9-19 | 26 | 11 | 8 | 30 | 50 | 25 |
| 1359 | 2019-9-20 | 12 | 4 | 4 | 17 | 37 | 23 |
| 1360 | 2019-9-21 | 7 | 5 | 2 | 14 | 25 | 16 |
| 1361 | 2019-9-22 | 9 | 4 | 3 | 26 | 46 | 24 |
| 1362 | 2019-9-23 | 31 | 16 | 9 | 33 | 60 | 31 |
| 1363 | 2019-9-24 | 20 | 10 | 5 | 51 | 81 | 36 |
| 1364 | 2019-9-25 | 13 | 4 | 4 | 32 | 39 | 22 |
| 1365 | 2019-9-26 | 14 | 5 | 4 | 30 | 43 | 38 |
| 1366 | 2019-9-27 | 11 | 4 | 3 | 32 | 56 | 38 |
| 1367 | 2019-9-28 | 7 | 0 | 3 | 37 | 63 | 35 |
| 1368 | 2019-9-29 | 10 | 5 | 1 | 40 | 64 | 30 |

| | | | | | | | |
|---|---|---|---|---|---|---|---|
| 1369 | 2019-9-30 | 10 | 6 | 2 | 33 | 55 | 29 |
| 1370 | 2019-10-1 | 7 | 4 | 1 | 29 | 48 | 29 |
| 1371 | 2019-10-2 | 8 | 5 | 1 | 22 | 37 | 20 |
| 1372 | 2019-10-3 | 5 | 2 | 0 | 19 | 36 | 16 |
| 1373 | 2019-10-4 | 7 | 2 | 3 | 20 | 42 | 33 |
| 1374 | 2019-10-5 | 10 | 5 | 0 | 17 | 29 | 22 |
| 1375 | 2019-10-6 | 5 | 2 | 0 | 25 | 42 | 33 |
| 1376 | 2019-10-7 | 23 | 6 | 9 | 25 | 49 | 89 |
| 1377 | 2019-10-8 | 28 | 10 | 9 | 29 | 51 | 28 |
| 1378 | 2019-10-9 | 25 | 16 | 4 | 27 | 47 | 26 |
| 1379 | 2019-10-10 | 24 | 13 | 4 | 23 | 44 | 26 |
| 1380 | 2019-10-11 | 21 | 9 | 3 | 21 | 41 | 27 |
| 1381 | 2019-10-12 | 12 | 6 | 1 | 20 | 35 | 29 |
| 1382 | 2019-10-13 | 9 | 1 | 5 | 25 | 48 | 37 |
| 1383 | 2019-10-14 | 34 | 11 | 10 | 25 | 50 | 36 |
| 1384 | 2019-10-15 | 20 | 6 | 7 | 21 | 35 | 56 |
| 1385 | 2019-10-16 | 24 | 9 | 9 | 18 | 33 | 33 |
| 1386 | 2019-10-17 | 15 | 6 | 5 | 20 | 39 | 31 |
| 1387 | 2019-10-18 | 7 | 3 | 2 | 23 | 35 | 24 |
| 1388 | 2019-10-19 | 7 | 3 | 3 | 32 | 52 | 32 |
| 1389 | 2019-10-20 | 6 | 3 | 2 | 32 | 54 | 25 |
| 1390 | 2019-10-21 | 39 | 13 | 17 | 31 | 57 | 26 |
| 1391 | 2019-10-22 | 26 | 12 | 6 | 28 | 56 | 25 |
| 1392 | 2019-10-23 | 25 | 10 | 7 | 28 | 54 | 26 |
| 1393 | 2019-10-24 | 17 | 8 | 3 | 36 | 64 | 38 |
| 1394 | 2019-10-25 | 12 | 5 | 1 | 42 | 76 | 38 |
| 1395 | 2019-10-26 | 5 | 1 | 1 | 46 | 82 | 42 |
| 1396 | 2019-10-27 | 12 | 5 | 0 | 33 | 60 | 31 |
| 1397 | 2019-10-28 | 31 | 8 | 10 | 22 | 38 | 25 |
| 1398 | 2019-10-29 | 18 | 10 | 7 | 29 | 49 | 34 |
| 1399 | 2019-10-30 | 16 | 9 | 3 | 31 | 53 | 31 |
| 1400 | 2019-10-31 | 16 | 5 | 8 | 29 | 50 | 26 |

| | | | | | | | |
|---|---|---|---|---|---|---|---|
| 1401 | 2019-11-1 | 18 | 12 | 2 | 30 | 53 | 35 |
| 1402 | 2019-11-2 | 5 | 2 | 2 | 24 | 47 | 30 |
| 1403 | 2019-11-3 | 5 | 2 | 2 | 27 | 51 | 29 |
| 1404 | 2019-11-4 | 27 | 14 | 5 | 31 | 58 | 35 |
| 1405 | 2019-11-5 | 24 | 9 | 3 | 31 | 60 | 33 |
| 1406 | 2019-11-6 | 16 | 7 | 7 | 27 | 55 | 32 |
| 1407 | 2019-11-7 | 22 | 9 | 4 | 31 | 59 | 32 |
| 1408 | 2019-11-8 | 13 | 6 | 4 | 31 | 58 | 30 |
| 1409 | 2019-11-9 | 5 | 2 | 1 | 30 | 58 | 28 |
| 1410 | 2019-11-10 | 6 | 2 | 1 | 29 | 59 | 25 |
| 1411 | 2019-11-11 | 40 | 12 | 10 | 30 | 58 | 50 |
| 1412 | 2019-11-12 | 23 | 10 | 3 | 27 | 53 | 27 |
| 1413 | 2019-11-13 | 12 | 7 | 2 | 25 | 53 | 29 |
| 1414 | 2019-11-14 | 20 | 7 | 9 | 31 | 59 | 38 |
| 1415 | 2019-11-15 | 6 | 4 | 2 | 44 | 76 | 29 |
| 1416 | 2019-11-16 | 7 | 2 | 1 | 35 | 68 | 29 |
| 1417 | 2019-11-17 | 7 | 4 | 2 | 34 | 69 | 35 |
| 1418 | 2019-11-18 | 25 | 12 | 5 | 22 | 51 | 38 |
| 1419 | 2019-11-19 | 24 | 12 | 8 | 28 | 55 | 47 |
| 1420 | 2019-11-20 | 9 | 4 | 4 | 26 | 57 | 30 |
| 1421 | 2019-11-21 | 7 | 1 | 5 | 30 | 61 | 34 |
| 1422 | 2019-11-22 | 13 | 6 | 4 | 30 | 59 | 32 |
| 1423 | 2019-11-23 | 10 | 8 | 0 | 32 | 62 | 35 |
| 1424 | 2019-11-24 | 4 | 3 | 1 | 34 | 61 | 27 |
| 1425 | 2019-11-25 | 24 | 9 | 5 | 28 | 51 | 38 |
| 1426 | 2019-11-26 | 17 | 5 | 7 | 37 | 72 | 38 |
| 1427 | 2019-11-27 | 16 | 7 | 6 | 36 | 67 | 35 |
| 1428 | 2019-11-28 | 16 | 5 | 2 | 38 | 69 | 40 |
| 1429 | 2019-11-29 | 16 | 7 | 6 | 37 | 68 | 62 |
| 1430 | 2019-11-30 | 3 | 3 | 0 | 33 | 66 | 80 |
| 1431 | 2019-12-1 | 9 | 5 | 2 | 30 | 61 | 63 |
| 1432 | 2019-12-2 | 29 | 8 | 12 | 29 | 55 | 29 |

| | | | | | | | |
|---|---|---|---|---|---|---|---|
| 1433 | 2019-12-3 | 23 | 9 | 7 | 35 | 54 | 32 |
| 1434 | 2019-12-4 | 12 | 9 | 1 | 24 | 34 | 24 |
| 1435 | 2019-12-5 | 8 | 4 | 1 | 23 | 32 | 29 |
| 1436 | 2019-12-6 | 13 | 4 | 5 | 33 | 60 | 28 |
| 1437 | 2019-12-7 | 4 | 1 | 2 | 43 | 69 | 34 |
| 1438 | 2019-12-8 | 8 | 3 | 3 | 47 | 73 | 40 |
| 1439 | 2019-12-9 | 20 | 9 | 6 | 45 | 70 | 42 |
| 1440 | 2019-12-10 | 17 | 6 | 7 | 50 | 79 | 45 |
| 1441 | 2019-12-11 | 21 | 12 | 3 | 61 | 95 | 63 |
| 1442 | 2019-12-12 | 18 | 8 | 4 | 51 | 80 | 38 |
| 1443 | 2019-12-13 | 10 | 2 | 3 | 52 | 81 | 46 |
| 1444 | 2019-12-14 | 13 | 1 | 7 | 53 | 88 | 49 |
| 1445 | 2019-12-15 | 8 | 2 | 5 | 49 | 90 | 61 |
| 1446 | 2019-12-16 | 25 | 9 | 8 | 41 | 84 | 54 |
| 1447 | 2019-12-17 | 17 | 8 | 5 | 44 | 85 | 57 |
| 1448 | 2019-12-18 | 17 | 5 | 5 | 42 | 77 | 51 |
| 1449 | 2019-12-19 | 13 | 6 | 4 | 43 | 83 | 41 |
| 1450 | 2019-12-20 | 14 | 6 | 4 | 43 | 80 | 36 |
| 1451 | 2019-12-21 | 9 | 1 | 4 | 43 | 82 | 46 |
| 1452 | 2019-12-22 | 7 | 2 | 4 | 31 | 72 | 50 |
| 1453 | 2019-12-23 | 31 | 8 | 13 | 29 | 63 | 39 |
| 1454 | 2019-12-24 | 27 | 9 | 10 | 33 | 71 | 46 |
| 1455 | 2019-12-25 | 13 | 8 | 2 | 33 | 74 | 60 |
| 1456 | 2019-12-26 | 22 | 10 | 9 | 36 | 73 | 36 |
| 1457 | 2019-12-27 | 13 | 4 | 6 | 42 | 63 | 28 |
| 1458 | 2019-12-28 | 7 | 2 | 2 | 26 | 38 | 25 |
| 1459 | 2019-12-29 | 9 | 4 | 2 | 33 | 54 | 27 |
| 1460 | 2019-12-30 | 24 | 12 | 4 | 37 | 64 | 27 |
| 1461 | 2019-12-31 | 13 | 6 | 4 | 42 | 71 | 28 |
| 1462 | 2020-1-1 | 6 | 4 | 1 | 44 | 69 | 23 |
| 1463 | 2020-1-2 | 29 | 6 | 9 | 42 | 76 | 34 |
| 1464 | 2020-1-3 | 12 | 5 | 3 | 64 | 102 | 41 |

| | | | | | | | |
|---|---|---|---|---|---|---|---|
| 1465 | 2020-1-4 | 12 | 2 | 7 | 57 | 84 | 34 |
| 1466 | 2020-1-5 | 12 | 3 | 5 | 52 | 69 | 25 |
| 1467 | 2020-1-6 | 29 | 11 | 10 | 29 | 46 | 18 |
| 1468 | 2020-1-7 | 18 | 7 | 3 | 26 | 49 | 28 |
| 1469 | 2020-1-8 | 18 | 2 | 8 | 43 | 69 | 28 |
| 1470 | 2020-1-9 | 20 | 8 | 5 | 35 | 63 | 38 |
| 1471 | 2020-1-10 | 10 | 3 | 2 | 24 | 40 | 26 |
| 1472 | 2020-1-11 | 5 | 2 | 2 | 30 | 51 | 27 |
| 1473 | 2020-1-12 | 7 | 2 | 3 | 38 | 64 | 30 |
| 1474 | 2020-1-13 | 31 | 7 | 12 | 54 | 78 | 36 |
| 1475 | 2020-1-14 | 12 | 3 | 5 | 64 | 91 | 37 |
| 1476 | 2020-1-15 | 8 | 1 | 3 | 43 | 69 | 34 |
| 1477 | 2020-1-16 | 20 | 14 | 2 | 33 | 56 | 23 |
| 1478 | 2020-1-17 | 15 | 3 | 4 | 59 | 88 | 37 |
| 1479 | 2020-1-18 | 11 | 3 | 4 | 56 | 85 | 40 |
| 1480 | 2020-1-19 | 12 | 2 | 3 | 45 | 71 | 36 |
| 1481 | 2020-1-20 | 17 | 10 | 3 | 32 | 52 | 29 |
| 1482 | 2020-1-21 | 9 | 4 | 2 | 29 | 44 | 21 |
| 1483 | 2020-1-22 | 4 | 3 | 0 | 21 | 35 | 18 |
| 1484 | 2020-1-23 | 3 | 1 | 1 | 20 | 31 | 20 |
| 1485 | 2020-1-24 | 5 | 3 | 2 | 25 | 36 | 32 |
| 1486 | 2020-1-25 | 6 | 4 | 2 | 16 | 23 | 17 |
| 1487 | 2020-1-26 | 7 | 4 | 0 | 22 | 29 | 35 |
| 1488 | 2020-1-27 | 6 | 2 | 0 | 31 | 41 | 30 |
| 1489 | 2020-1-28 | 11 | 4 | 4 | 30 | 43 | 27 |
| 1490 | 2020-1-29 | 20 | 8 | 7 | 27 | 36 | 26 |
| 1491 | 2020-1-30 | 7 | 3 | 0 | 32 | 44 | 28 |
| 1492 | 2020-1-31 | 4 | 2 | 1 | 29 | 43 | 22 |
| 1493 | 2020-2-1 | 2 | 1 | 1 | 22 | 31 | 20 |
| 1494 | 2020-2-2 | 4 | 2 | 1 | 23 | 32 | 27 |
| 1495 | 2020-2-3 | 2 | 2 | 0 | 22 | 33 | 23 |
| 1496 | 2020-2-4 | 5 | 3 | 1 | 29 | 40 | 26 |

| | | | | | | | |
|---|---|---|---|---|---|---|---|
| 1497 | 2020-2-5 | 10 | 5 | 4 | 28 | 42 | 28 |
| 1498 | 2020-2-6 | 6 | 2 | 1 | 19 | 29 | 17 |
| 1499 | 2020-2-7 | 1 | 0 | 1 | 38 | 53 | 33 |
| 1500 | 2020-2-8 | 2 | 1 | 0 | 41 | 58 | 35 |
| 1501 | 2020-2-9 | 0 | 0 | 0 | 46 | 65 | 51 |
| 1502 | 2020-2-10 | 8 | 5 | 3 | 47 | 57 | 50 |
| 1503 | 2020-2-11 | 6 | 1 | 3 | 22 | 34 | 24 |
| 1504 | 2020-2-12 | 4 | 1 | 1 | 23 | 38 | 26 |
| 1505 | 2020-2-13 | 8 | 2 | 1 | 30 | 49 | 31 |
| 1506 | 2020-2-14 | 6 | 2 | 2 | 31 | 45 | 28 |
| 1507 | 2020-2-15 | 4 | 2 | 0 | 30 | 46 | 45 |
| 1508 | 2020-2-16 | 6 | 3 | 2 | 27 | 39 | 40 |
| 1509 | 2020-2-17 | 9 | 2 | 4 | 47 | 68 | 57 |
| 1510 | 2020-2-18 | 12 | 6 | 3 | 46 | 61 | 52 |
| 1511 | 2020-2-19 | 15 | 7 | 3 | 42 | 55 | 22 |
| 1512 | 2020-2-20 | 8 | 4 | 3 | 39 | 57 | 30 |
| 1513 | 2020-2-21 | 9 | 0 | 2 | 38 | 61 | 37 |
| 1514 | 2020-2-22 | 5 | 0 | 3 | 44 | 69 | 36 |
| 1515 | 2020-2-23 | 3 | 1 | 0 | 47 | 71 | 34 |
| 1516 | 2020-2-24 | 14 | 3 | 5 | 33 | 45 | 15 |
| 1517 | 2020-2-25 | 22 | 7 | 8 | 26 | 40 | 15 |
| 1518 | 2020-2-26 | 3 | 1 | 1 | 29 | 49 | 21 |
| 1519 | 2020-2-27 | 17 | 4 | 7 | 43 | 63 | 20 |
| 1520 | 2020-2-28 | 10 | 4 | 2 | 18 | 25 | 15 |
| 1521 | 2020-2-29 | 8 | 5 | 2 | 21 | 36 | 20 |
| 1522 | 2020-3-1 | 8 | 2 | 0 | 27 | 47 | 25 |
| 1523 | 2020-3-2 | 20 | 10 | 6 | 19 | 32 | 21 |
| 1524 | 2020-3-3 | 19 | 11 | 4 | 25 | 41 | 27 |
| 1525 | 2020-3-4 | 13 | 4 | 3 | 24 | 39 | 22 |
| 1526 | 2020-3-5 | 14 | 6 | 3 | 29 | 51 | 23 |
| 1527 | 2020-3-6 | 10 | 4 | 5 | 24 | 39 | 15 |
| 1528 | 2020-3-7 | 6 | 2 | 1 | 21 | 35 | 17 |

| | | | | | | | |
|---|---|---|---|---|---|---|---|
| 1529 | 2020-3-8 | 6 | 3 | 3 | 14 | 28 | 13 |
| 1530 | 2020-3-9 | 23 | 10 | 4 | 15 | 29 | 19 |
| 1531 | 2020-3-10 | 23 | 9 | 6 | 25 | 44 | 21 |
| 1532 | 2020-3-11 | 11 | 6 | 1 | 29 | 54 | 32 |
| 1533 | 2020-3-12 | 12 | 4 | 5 | 21 | 37 | 17 |
| 1534 | 2020-3-13 | 6 | 1 | 3 | 20 | 36 | 21 |
| 1535 | 2020-3-14 | 5 | 1 | 1 | 31 | 60 | 29 |
| 1536 | 2020-3-15 | 3 | 2 | 0 | 30 | 63 | 40 |
| 1537 | 2020-3-16 | 32 | 6 | 14 | 31 | 56 | 22 |
| 1538 | 2020-3-17 | 14 | 7 | 2 | 33 | 54 | 21 |
| 1539 | 2020-3-18 | 15 | 4 | 1 | 31 | 53 | 17 |
| 1540 | 2020-3-19 | 8 | 4 | 1 | 30 | 56 | 18 |
| 1541 | 2020-3-20 | 14 | 7 | 3 | 39 | 70 | 25 |
| 1542 | 2020-3-21 | 12 | 7 | 2 | 31 | 48 | 14 |
| 1543 | 2020-3-22 | 9 | 5 | 1 | 47 | 73 | 26 |
| 1544 | 2020-3-23 | 30 | 6 | 7 | 42 | 68 | 24 |
| 1545 | 2020-3-24 | 17 | 6 | 5 | 33 | 55 | 22 |
| 1546 | 2020-3-25 | 16 | 4 | 2 | 22 | 38 | 14 |
| 1547 | 2020-3-26 | 13 | 6 | 3 | 22 | 42 | 17 |
| 1548 | 2020-3-27 | 13 | 7 | 1 | 26 | 46 | 16 |
| 1549 | 2020-3-28 | 13 | 3 | 5 | 34 | 54 | 15 |
| 1550 | 2020-3-29 | 5 | 3 | 1 | 34 | 59 | 13 |
| 1551 | 2020-3-30 | 32 | 6 | 10 | 27 | 50 | 15 |
| 1552 | 2020-3-31 | 20 | 8 | 5 | 31 | 53 | 19 |
| 1553 | 2020-4-1 | 24 | 7 | 8 | 49 | 74 | 16 |
| 1554 | 2020-4-2 | 15 | 5 | 2 | 72 | 98 | 15 |
| 1555 | 2020-4-3 | 9 | 2 | 5 | 57 | 80 | 17 |
| 1556 | 2020-4-4 | 7 | 5 | 0 | 47 | 68 | 14 |
| 1557 | 2020-4-5 | 5 | 2 | 0 | 37 | 58 | 20 |
| 1558 | 2020-4-6 | 5 | 2 | 1 | 31 | 52 | 14 |
| 1559 | 2020-4-7 | 37 | 16 | 8 | 34 | 54 | 15 |
| 1560 | 2020-4-8 | 16 | 5 | 4 | 33 | 55 | 20 |

| | | | | | | | |
|---|---|---|---|---|---|---|---|
| 1561 | 2020-4-9 | 18 | 10 | 5 | 31 | 55 | 25 |
| 1562 | 2020-4-10 | 18 | 5 | 5 | 30 | 54 | 12 |
| 1563 | 2020-4-11 | 5 | 2 | 0 | 29 | 52 | 25 |
| 1564 | 2020-4-12 | 5 | 2 | 2 | 28 | 53 | 22 |
| 1565 | 2020-4-13 | 25 | 9 | 9 | 32 | 51 | 27 |
| 1566 | 2020-4-14 | 19 | 10 | 1 | 28 | 51 | 44 |
| 1567 | 2020-4-15 | 14 | 8 | 4 | 38 | 70 | 38 |
| 1568 | 2020-4-16 | 8 | 3 | 1 | 36 | 64 | 18 |
| 1569 | 2020-4-17 | 14 | 5 | 2 | 29 | 55 | 14 |
| 1570 | 2020-4-18 | 8 | 3 | 3 | 19 | 36 | 13 |
| 1571 | 2020-4-19 | 5 | 1 | 2 | 23 | 41 | 16 |
| 1572 | 2020-4-20 | 28 | 8 | 9 | 21 | 44 | 15 |
| 1573 | 2020-4-21 | 19 | 7 | 8 | 22 | 46 | 13 |
| 1574 | 2020-4-22 | 11 | 3 | 4 | 19 | 38 | 13 |
| 1575 | 2020-4-23 | 12 | 1 | 5 | 16 | 33 | 13 |
| 1576 | 2020-4-24 | 17 | 9 | 4 | 12 | 26 | 14 |
| 1577 | 2020-4-25 | 4 | 3 | 1 | 21 | 35 | 15 |
| 1578 | 2020-4-26 | 28 | 10 | 7 | 34 | 60 | 30 |
| 1579 | 2020-4-27 | 17 | 10 | 6 | 30 | 57 | 21 |
| 1580 | 2020-4-28 | 17 | 5 | 5 | 32 | 57 | 19 |
| 1581 | 2020-4-29 | 14 | 5 | 2 | 30 | 53 | 16 |
| 1582 | 2020-4-30 | 9 | 5 | 3 | 29 | 49 | 20 |
| 1583 | 2020-5-1 | 5 | 2 | 1 | 15 | 30 | 14 |
| 1584 | 2020-5-2 | 5 | 2 | 2 | 17 | 30 | 18 |
| 1585 | 2020-5-3 | 8 | 3 | 1 | 25 | 47 | 18 |
| 1586 | 2020-5-4 | 4 | 1 | 0 | 19 | 38 | 17 |
| 1587 | 2020-5-5 | 12 | 4 | 2 | 16 | 29 | 12 |
| 1588 | 2020-5-6 | 35 | 14 | 6 | 13 | 28 | 14 |
| 1589 | 2020-5-7 | 26 | 8 | 9 | 14 | 31 | 11 |
| 1590 | 2020-5-8 | 23 | 12 | 3 | 13 | 30 | 10 |
| 1591 | 2020-5-9 | 12 | 5 | 2 | 17 | 34 | 10 |
| 1592 | 2020-5-10 | 5 | 2 | 0 | 20 | 38 | 12 |

| | | | | | | | |
|---|---|---|---|---|---|---|---|
| 1593 | 2020-5-11 | 37 | 15 | 8 | 21 | 39 | 11 |
| 1594 | 2020-5-12 | 20 | 9 | 8 | 27 | 50 | 19 |
| 1595 | 2020-5-13 | 18 | 8 | 0 | 27 | 49 | 14 |
| 1596 | 2020-5-14 | 13 | 7 | 1 | 19 | 36 | 15 |
| 1597 | 2020-5-15 | 15 | 8 | 3 | 19 | 34 | 15 |
| 1598 | 2020-5-16 | 3 | 2 | 0 | 24 | 46 | 18 |
| 1599 | 2020-5-17 | 5 | 2 | 1 | 12 | 27 | 13 |
| 1600 | 2020-5-18 | 21 | 3 | 9 | 21 | 36 | 30 |
| 1601 | 2020-5-19 | 32 | 9 | 8 | 22 | 41 | 29 |
| 1602 | 2020-5-20 | 14 | 7 | 3 | 22 | 42 | 11 |
| 1603 | 2020-5-21 | 15 | 5 | 3 | 12 | 29 | 14 |
| 1604 | 2020-5-22 | 13 | 7 | 1 | 12 | 32 | 15 |
| 1605 | 2020-5-23 | 3 | 2 | 1 | 17 | 35 | 17 |
| 1606 | 2020-5-24 | 9 | 4 | 1 | 28 | 46 | 26 |
| 1607 | 2020-5-25 | 27 | 9 | 8 | 16 | 33 | 19 |
| 1608 | 2020-5-26 | 21 | 7 | 8 | 13 | 24 | 15 |
| 1609 | 2020-5-27 | 15 | 8 | 3 | 26 | 26 | 11 |
| 1610 | 2020-5-28 | 16 | 7 | 4 | 31 | 36 | 14 |
| 1611 | 2020-5-29 | 12 | 6 | 4 | 29 | 39 | 24 |
| 1612 | 2020-5-30 | 12 | 6 | 2 | 34 | 56 | 26 |
| 1613 | 2020-5-31 | 10 | 5 | 3 | 28 | 48 | 31 |
| 1614 | 2020-6-1 | 23 | 8 | 7 | 20 | 38 | 20 |
| 1615 | 2020-6-2 | 20 | 7 | 7 | 15 | 31 | 12 |
| 1616 | 2020-6-3 | 26 | 14 | 7 | 16 | 34 | 18 |
| 1617 | 2020-6-4 | 17 | 4 | 9 | 21 | 42 | 26 |
| 1618 | 2020-6-5 | 16 | 6 | 6 | 19 | 39 | 24 |
| 1619 | 2020-6-6 | 8 | 1 | 1 | 19 | 35 | 24 |
| 1620 | 2020-6-7 | 8 | 5 | 2 | 13 | 27 | 13 |
| 1621 | 2020-6-8 | 23 | 9 | 6 | 13 | 27 | 17 |
| 1622 | 2020-6-9 | 20 | 5 | 10 | 15 | 33 | 22 |
| 1623 | 2020-6-10 | 18 | 5 | 8 | 20 | 36 | 39 |
| 1624 | 2020-6-11 | 6 | 1 | 1 | 12 | 25 | 18 |

| | | | | | | | |
|---|---|---|---|---|---|---|---|
| 1625 | 2020-6-12 | 15 | 7 | 1 | 7 | 20 | 14 |
| 1626 | 2020-6-13 | 4 | 1 | 1 | 12 | 24 | 21 |
| 1627 | 2020-6-14 | 2 | 1 | 1 | 12 | 23 | 16 |
| 1628 | 2020-6-15 | 18 | 4 | 8 | 25 | 37 | 33 |
| 1629 | 2020-6-16 | 22 | 10 | 9 | 21 | 39 | 25 |
| 1630 | 2020-6-17 | 17 | 6 | 5 | 13 | 30 | 21 |
| 1631 | 2020-6-18 | 18 | 6 | 7 | 10 | 25 | 20 |
| 1632 | 2020-6-19 | 10 | 3 | 3 | 24 | 37 | 26 |
| 1633 | 2020-6-20 | 11 | 3 | 2 | 10 | 20 | 11 |
| 1634 | 2020-6-21 | 2 | 1 | 1 | 8 | 21 | 14 |
| 1635 | 2020-6-22 | 20 | 11 | 5 | 10 | 24 | 20 |
| 1636 | 2020-6-23 | 12 | 7 | 2 | 9 | 21 | 12 |
| 1637 | 2020-6-24 | 3 | 2 | 0 | 14 | 29 | 21 |
| 1638 | 2020-6-25 | 3 | 1 | 2 | 21 | 36 | 33 |
| 1639 | 2020-6-26 | 5 | 2 | 2 | 11 | 23 | 19 |
| 1640 | 2020-6-27 | 7 | 5 | 0 | 10 | 21 | 12 |
| 1641 | 2020-6-28 | 17 | 6 | 4 | 9 | 21 | 12 |
| 1642 | 2020-6-29 | 14 | 6 | 5 | 9 | 20 | 10 |
| 1643 | 2020-6-30 | 19 | 7 | 5 | 14 | 27 | 22 |
| 1644 | 2020-7-1 | 10 | 5 | 1 | 24 | 35 | 22 |
| 1645 | 2020-7-2 | 22 | 11 | 5 | 28 | 45 | 22 |
| 1646 | 2020-7-3 | 11 | 6 | 1 | 28 | 49 | 67 |
| 1647 | 2020-7-4 | 12 | 8 | 3 | 19 | 34 | 48 |
| 1648 | 2020-7-5 | 4 | 0 | 1 | 18 | 34 | 29 |
| 1649 | 2020-7-6 | 21 | 8 | 7 | 9 | 23 | 21 |
| 1650 | 2020-7-7 | 15 | 6 | 3 | 9 | 22 | 11 |
| 1651 | 2020-7-8 | 15 | 6 | 7 | 15 | 25 | 25 |
| 1652 | 2020-7-9 | 16 | 8 | 0 | 19 | 31 | 23 |
| 1653 | 2020-7-10 | 13 | 4 | 2 | 16 | 30 | 24 |
| 1654 | 2020-7-11 | 14 | 10 | 1 | 11 | 38 | 28 |
| 1655 | 2020-7-12 | 4 | 1 | 1 | 13 | 33 | 25 |
| 1656 | 2020-7-13 | 19 | 6 | 6 | 23 | 35 | 20 |

| | | | | | | | |
|---|---|---|---|---|---|---|---|
| 1657 | 2020-7-14 | 25 | 9 | 6 | 14 | 29 | 35 |
| 1658 | 2020-7-15 | 18 | 5 | 7 | 8 | 20 | 14 |
| 1659 | 2020-7-16 | 6 | 2 | 3 | 7 | 19 | 13 |
| 1660 | 2020-7-17 | 10 | 3 | 0 | 17 | 26 | 23 |
| 1661 | 2020-7-18 | 6 | 1 | 1 | 22 | 32 | 38 |
| 1662 | 2020-7-19 | 15 | 5 | 5 | 17 | 28 | 28 |
| 1663 | 2020-7-20 | 22 | 10 | 2 | 21 | 29 | 16 |
| 1664 | 2020-7-21 | 22 | 6 | 10 | 23 | 40 | 24 |
| 1665 | 2020-7-22 | 6 | 2 | 0 | 8 | 19 | 10 |
| 1666 | 2020-7-23 | 10 | 2 | 3 | 8 | 18 | 13 |
| 1667 | 2020-7-24 | 15 | 6 | 3 | 10 | 17 | 17 |
| 1668 | 2020-7-25 | 4 | 1 | 0 | 11 | 22 | 14 |
| 1669 | 2020-7-26 | 5 | 3 | 1 | 12 | 26 | 21 |
| 1670 | 2020-7-27 | 27 | 9 | 10 | 25 | 44 | 22 |
| 1671 | 2020-7-28 | 18 | 3 | 8 | 37 | 66 | 24 |
| 1672 | 2020-7-29 | 9 | 2 | 3 | 31 | 56 | 21 |
| 1673 | 2020-7-30 | 9 | 3 | 4 | 25 | 47 | 17 |
| 1674 | 2020-7-31 | 20 | 7 | 5 | 25 | 43 | 14 |
| 1675 | 2020-8-1 | 2 | 1 | 1 | 26 | 36 | 19 |
| 1676 | 2020-8-2 | 7 | 2 | 3 | 33 | 50 | 27 |
| 1677 | 2020-8-3 | 25 | 9 | 8 | 22 | 38 | 24 |
| 1678 | 2020-8-4 | 18 | 4 | 4 | 27 | 43 | 37 |
| 1679 | 2020-8-5 | 14 | 5 | 3 | 30 | 46 | 26 |
| 1680 | 2020-8-6 | 22 | 8 | 8 | 26 | 45 | 22 |
| 1681 | 2020-8-7 | 16 | 7 | 6 | 19 | 32 | 15 |
| 1682 | 2020-8-8 | 4 | 0 | 3 | 15 | 28 | 25 |
| 1683 | 2020-8-9 | 9 | 1 | 5 | 18 | 28 | 18 |
| 1684 | 2020-8-10 | 15 | 5 | 2 | 14 | 26 | 24 |
| 1685 | 2020-8-11 | 37 | 12 | 15 | 23 | 41 | 41 |
| 1686 | 2020-8-12 | 15 | 4 | 8 | 17 | 34 | 21 |
| 1687 | 2020-8-13 | 18 | 7 | 6 | 20 | 34 | 28 |
| 1688 | 2020-8-14 | 15 | 5 | 5 | 20 | 32 | 17 |

| | | | | | | | |
|---|---|---|---|---|---|---|---|
| 1689 | 2020-8-15 | 7 | 5 | 2 | 19 | 37 | 31 |
| 1690 | 2020-8-16 | 10 | 5 | 2 | 21 | 39 | 37 |
| 1691 | 2020-8-17 | 18 | 6 | 7 | 22 | 34 | 43 |
| 1692 | 2020-8-18 | 16 | 7 | 4 | 11 | 21 | 11 |
| 1693 | 2020-8-19 | 14 | 7 | 3 | 11 | 14 | 10 |
| 1694 | 2020-8-20 | 9 | 5 | 3 | 21 | 37 | 23 |
| 1695 | 2020-8-21 | 16 | 2 | 8 | 39 | 59 | 29 |
| 1696 | 2020-8-22 | 8 | 2 | 2 | 34 | 55 | 38 |
| 1697 | 2020-8-23 | 7 | 3 | 4 | 13 | 28 | 19 |
| 1698 | 2020-8-24 | 33 | 11 | 11 | 13 | 24 | 17 |
| 1699 | 2020-8-25 | 23 | 2 | 15 | 28 | 49 | 30 |
| 1700 | 2020-8-26 | 13 | 5 | 4 | 48 | 75 | 43 |
| 1701 | 2020-8-27 | 14 | 6 | 1 | 49 | 74 | 34 |
| 1702 | 2020-8-28 | 16 | 8 | 1 | 31 | 46 | 28 |
| 1703 | 2020-8-29 | 7 | 3 | 2 | 20 | 30 | 17 |
| 1704 | 2020-8-30 | 5 | 2 | 3 | 24 | 41 | 18 |
| 1705 | 2020-8-31 | 13 | 4 | 2 | 20 | 30 | 18 |
| 1706 | 2020-9-1 | 22 | 7 | 7 | 28 | 43 | 21 |
| 1707 | 2020-9-2 | 11 | 5 | 1 | 38 | 59 | 26 |
| 1708 | 2020-9-3 | 9 | 1 | 3 | 39 | 65 | 31 |
| 1709 | 2020-9-4 | 11 | 3 | 5 | 46 | 69 | 20 |
| 1710 | 2020-9-5 | 5 | 3 | 0 | 31 | 49 | 26 |
| 1711 | 2020-9-6 | 7 | 4 | 0 | 25 | 42 | 30 |
| 1712 | 2020-9-7 | 28 | 10 | 8 | 28 | 50 | 22 |
| 1713 | 2020-9-8 | 20 | 5 | 11 | 40 | 63 | 34 |
| 1714 | 2020-9-9 | 15 | 3 | 8 | 52 | 73 | 50 |
| 1715 | 2020-9-10 | 12 | 6 | 2 | 28 | 40 | 23 |
| 1716 | 2020-9-11 | 14 | 5 | 5 | 24 | 41 | 31 |
| 1717 | 2020-9-12 | 13 | 5 | 6 | 21 | 37 | 30 |
| 1718 | 2020-9-13 | 9 | 2 | 4 | 21 | 32 | 25 |
| 1719 | 2020-9-14 | 25 | 5 | 9 | 24 | 38 | 29 |
| 1720 | 2020-9-15 | 16 | 3 | 6 | 27 | 46 | 40 |

| | | | | | | | |
|---|---|---|---|---|---|---|---|
| 1721 | 2020-9-16 | 12 | 3 | 3 | 24 | 43 | 28 |
| 1722 | 2020-9-17 | 8 | 3 | 2 | 15 | 30 | 19 |
| 1723 | 2020-9-18 | 7 | 4 | 2 | 20 | 34 | 27 |
| 1724 | 2020-9-19 | 7 | 1 | 2 | 31 | 49 | 26 |
| 1725 | 2020-9-20 | 10 | 4 | 2 | 35 | 59 | 35 |
| 1726 | 2020-9-21 | 25 | 11 | 5 | 26 | 45 | 37 |
| 1727 | 2020-9-22 | 25 | 9 | 7 | 20 | 36 | 44 |
| 1728 | 2020-9-23 | 11 | 4 | 5 | 13 | 25 | 15 |
| 1729 | 2020-9-24 | 16 | 4 | 5 | 30 | 49 | 30 |
| 1730 | 2020-9-25 | 9 | 2 | 3 | 40 | 62 | 37 |
| 1731 | 2020-9-26 | 4 | 2 | 1 | 18 | 37 | 21 |
| 1732 | 2020-9-27 | 5 | 4 | 0 | 31 | 52 | 41 |
| 1733 | 2020-9-28 | 13 | 5 | 4 | 38 | 58 | 29 |
| 1734 | 2020-9-29 | 18 | 4 | 6 | 24 | 44 | 23 |
| 1735 | 2020-9-30 | 7 | 3 | 1 | 35 | 53 | 61 |
| 1736 | 2020-10-1 | 9 | 3 | 5 | 26 | 46 | 28 |
| 1737 | 2020-10-2 | 8 | 4 | 2 | 16 | 32 | 20 |
| 1738 | 2020-10-3 | 9 | 6 | 0 | 16 | 34 | 26 |
| 1739 | 2020-10-4 | 9 | 4 | 2 | 17 | 34 | 31 |
| 1740 | 2020-10-5 | 11 | 8 | 1 | 20 | 42 | 35 |
| 1741 | 2020-10-6 | 8 | 3 | 4 | 12 | 28 | 25 |
| 1742 | 2020-10-7 | 5 | 3 | 2 | 21 | 44 | 37 |
| 1743 | 2020-10-8 | 22 | 12 | 3 | 24 | 43 | 49 |
| 1744 | 2020-10-9 | 29 | 7 | 10 | 20 | 36 | 20 |
| 1745 | 2020-10-10 | 18 | 8 | 6 | 30 | 52 | 40 |
| 1746 | 2020-10-11 | 10 | 3 | 2 | 26 | 45 | 23 |
| 1747 | 2020-10-12 | 27 | 12 | 7 | 18 | 31 | 18 |
| 1748 | 2020-10-13 | 23 | 7 | 12 | 30 | 49 | 39 |
| 1749 | 2020-10-14 | 20 | 6 | 7 | 32 | 53 | 36 |
| 1750 | 2020-10-15 | 22 | 9 | 6 | 29 | 48 | 41 |
| 1751 | 2020-10-16 | 16 | 5 | 5 | 17 | 27 | 41 |
| 1752 | 2020-10-17 | 11 | 4 | 5 | 16 | 29 | 35 |

| | | | | | | | |
|---|---|---|---|---|---|---|---|
| 1753 | 2020-10-18 | 6 | 3 | 2 | 27 | 39 | 40 |
| 1754 | 2020-10-19 | 27 | 6 | 8 | 19 | 29 | 34 |
| 1755 | 2020-10-20 | 19 | 4 | 6 | 23 | 33 | 43 |
| 1756 | 2020-10-21 | 16 | 5 | 4 | 11 | 18 | 15 |
| 1757 | 2020-10-22 | 16 | 5 | 5 | 35 | 51 | 24 |
| 1758 | 2020-10-23 | 7 | 2 | 3 | 49 | 73 | 31 |
| 1759 | 2020-10-24 | 3 | 0 | 1 | 37 | 63 | 26 |
| 1760 | 2020-10-25 | 9 | 3 | 5 | 48 | 70 | 32 |
| 1761 | 2020-10-26 | 24 | 7 | 6 | 36 | 58 | 29 |
| 1762 | 2020-10-27 | 28 | 10 | 8 | 33 | 57 | 29 |
| 1763 | 2020-10-28 | 19 | 8 | 5 | 24 | 49 | 28 |
| 1764 | 2020-10-29 | 12 | 3 | 2 | 32 | 54 | 31 |
| 1765 | 2020-10-30 | 13 | 3 | 5 | 28 | 47 | 34 |
| 1766 | 2020-10-31 | 9 | 6 | 1 | 24 | 46 | 38 |
| 1767 | 2020-11-1 | 7 | 3 | 2 | 24 | 45 | 25 |
| 1768 | 2020-11-2 | 27 | 8 | 8 | 16 | 33 | 16 |
| 1769 | 2020-11-3 | 22 | 6 | 11 | 27 | 40 | 51 |
| 1770 | 2020-11-4 | 13 | 6 | 2 | 31 | 44 | 34 |
| 1771 | 2020-11-5 | 21 | 5 | 8 | 49 | 71 | 23 |
| 1772 | 2020-11-6 | 14 | 1 | 8 | 36 | 58 | 21 |
| 1773 | 2020-11-7 | 8 | 2 | 4 | 29 | 49 | 17 |
| 1774 | 2020-11-8 | 4 | 2 | 0 | 38 | 61 | 24 |
| 1775 | 2020-11-9 | 18 | 5 | 7 | 37 | 60 | 17 |
| 1776 | 2020-11-10 | 21 | 10 | 5 | 49 | 66 | 19 |
| 1777 | 2020-11-11 | 21 | 5 | 7 | 73 | 103 | 26 |
| 1778 | 2020-11-12 | 14 | 3 | 5 | 59 | 80 | 28 |
| 1779 | 2020-11-13 | 12 | 5 | 4 | 52 | 79 | 34 |
| 1780 | 2020-11-14 | 9 | 2 | 3 | 47 | 68 | 23 |
| 1781 | 2020-11-15 | 7 | 3 | 3 | 52 | 75 | 26 |
| 1782 | 2020-11-16 | 27 | 6 | 11 | 54 | 75 | 27 |
| 1783 | 2020-11-17 | 26 | 6 | 11 | 37 | 59 | 22 |
| 1784 | 2020-11-18 | 13 | 3 | 5 | 23 | 45 | 26 |

| | | | | | | | |
|---|---|---|---|---|---|---|---|
| 1785 | 2020-11-19 | 10 | 2 | 5 | 27 | 49 | 30 |
| 1786 | 2020-11-20 | 18 | 2 | 10 | 30 | 52 | 24 |
| 1787 | 2020-11-21 | 4 | 1 | 0 | 39 | 68 | 34 |
| 1788 | 2020-11-22 | 6 | 2 | 0 | 45 | 79 | 35 |
| 1789 | 2020-11-23 | 27 | 8 | 4 | 45 | 74 | 36 |
| 1790 | 2020-11-24 | 20 | 7 | 4 | 37 | 61 | 26 |
| 1791 | 2020-11-25 | 11 | 4 | 3 | 40 | 70 | 41 |
| 1792 | 2020-11-26 | 22 | 7 | 11 | 40 | 67 | 32 |
| 1793 | 2020-11-27 | 18 | 6 | 7 | 49 | 74 | 34 |
| 1794 | 2020-11-28 | 6 | 5 | 1 | 47 | 76 | 30 |
| 1795 | 2020-11-29 | 14 | 7 | 2 | 38 | 60 | 34 |
| 1796 | 2020-11-30 | 30 | 4 | 9 | 51 | 78 | 31 |
| 1797 | 2020-12-1 | 21 | 7 | 7 | 39 | 67 | 26 |
| 1798 | 2020-12-2 | 9 | 6 | 2 | 41 | 69 | 28 |
| 1799 | 2020-12-3 | 20 | 7 | 4 | 32 | 56 | 22 |
| 1800 | 2020-12-4 | 20 | 6 | 4 | 39 | 61 | 27 |
| 1801 | 2020-12-5 | 11 | 2 | 3 | 38 | 61 | 26 |
| 1802 | 2020-12-6 | 6 | 1 | 3 | 31 | 57 | 34 |
| 1803 | 2020-12-7 | 21 | 4 | 9 | 31 | 59 | 25 |
| 1804 | 2020-12-8 | 19 | 6 | 8 | 44 | 71 | 27 |
| 1805 | 2020-12-9 | 23 | 9 | 6 | 50 | 77 | 24 |
| 1806 | 2020-12-10 | 14 | 4 | 3 | 47 | 73 | 24 |
| 1807 | 2020-12-11 | 11 | 5 | 3 | 51 | 77 | 23 |
| 1808 | 2020-12-12 | 4 | 1 | 0 | 52 | 83 | 28 |
| 1809 | 2020-12-13 | 10 | 4 | 5 | 48 | 81 | 22 |
| 1810 | 2020-12-14 | 30 | 10 | 9 | 36 | 71 | 30 |
| 1811 | 2020-12-15 | 18 | 2 | 4 | 36 | 73 | 31 |
| 1812 | 2020-12-16 | 25 | 10 | 8 | 38 | 71 | 37 |
| 1813 | 2020-12-17 | 14 | 3 | 5 | 37 | 73 | 31 |
| 1814 | 2020-12-18 | 13 | 4 | 5 | 40 | 68 | 41 |
| 1815 | 2020-12-19 | 6 | 3 | 1 | 46 | 77 | 28 |
| 1816 | 2020-12-20 | 6 | 2 | 3 | 46 | 67 | 24 |

| | | | | | | | |
|---|---|---|---|---|---|---|---|
| 1817 | 2020-12-21 | 30 | 6 | 9 | 49 | 71 | 38 |
| 1818 | 2020-12-22 | 17 | 4 | 4 | 48 | 70 | 35 |
| 1819 | 2020-12-23 | 21 | 9 | 4 | 54 | 76 | 26 |
| 1820 | 2020-12-24 | 11 | 3 | 1 | 69 | 91 | 28 |
| 1821 | 2020-12-25 | 17 | 7 | 5 | 74 | 98 | 32 |
| 1822 | 2020-12-26 | 9 | 5 | 2 | 52 | 88 | 46 |
| 1823 | 2020-12-27 | 7 | 3 | 1 | 68 | 99 | 35 |
| 1824 | 2020-12-28 | 17 | 5 | 6 | 88 | 117 | 37 |
| 1825 | 2020-12-29 | 14 | 3 | 4 | 88 | 121 | 47 |
| 1826 | 2020-12-30 | 11 | 5 | 2 | 86 | 113 | 22 |
| 1827 | 2020-12-31 | 11 | 3 | 4 | 134 | 157 | 44 |
